# Conventional versus hypofractionated high-dose intensity-modulated radiotherapy for prostate cancer: 5-year outcomes of the randomised, non-inferiority, phase 3 CHHiP trial

**DOI:** 10.1016/S1470-2045(16)30102-4

**Published:** 2016-08

**Authors:** David Dearnaley, Isabel Syndikus, Helen Mossop, Vincent Khoo, Alison Birtle, David Bloomfield, John Graham, Peter Kirkbride, John Logue, Zafar Malik, Julian Money-Kyrle, Joe M O'Sullivan, Miguel Panades, Chris Parker, Helen Patterson, Christopher Scrase, John Staffurth, Andrew Stockdale, Jean Tremlett, Margaret Bidmead, Helen Mayles, Olivia Naismith, Chris South, Annie Gao, Clare Cruickshank, Shama Hassan, Julia Pugh, Clare Griffin, Emma Hall, CHHiP Investigators

**Affiliations:** a The Institute of Cancer Research, London, UK; b Royal Marsden NHS Foundation Trust, London, UK; c Clatterbridge Cancer Centre, Wirral, UK; d Rosemere Cancer Centre, Royal Preston Hospital, Preston, UK; e Brighton and Sussex University Hospitals, Brighton, UK; f Beacon Centre, Musgrove Park Hospital, Taunton, UK; g Sheffield Teaching Hospitals Foundation Trust, Sheffield, UK; h Christie Hospital, Manchester, UK; i Royal Surrey County Hospital, Guildford, UK; j Queen's University Belfast, Belfast, UK; k Lincoln County Hospital, Lincoln, UK; l Addenbrooke's Hospital, Cambridge, UK; m Ipswich Hospital, Ipswich, UK; n Cardiff University/Velindre Cancer Centre, Cardiff, UK; o University Hospital Coventry, Coventry, UK

## Abstract

**Background:**

Prostate cancer might have high radiation-fraction sensitivity that would give a therapeutic advantage to hypofractionated treatment. We present a pre-planned analysis of the efficacy and side-effects of a randomised trial comparing conventional and hypofractionated radiotherapy after 5 years follow-up.

**Methods:**

CHHiP is a randomised, phase 3, non-inferiority trial that recruited men with localised prostate cancer (pT1b–T3aN0M0). Patients were randomly assigned (1:1:1) to conventional (74 Gy delivered in 37 fractions over 7·4 weeks) or one of two hypofractionated schedules (60 Gy in 20 fractions over 4 weeks or 57 Gy in 19 fractions over 3·8 weeks) all delivered with intensity-modulated techniques. Most patients were given radiotherapy with 3–6 months of neoadjuvant and concurrent androgen suppression. Randomisation was by computer-generated random permuted blocks, stratified by National Comprehensive Cancer Network (NCCN) risk group and radiotherapy treatment centre, and treatment allocation was not masked. The primary endpoint was time to biochemical or clinical failure; the critical hazard ratio (HR) for non-inferiority was 1·208. Analysis was by intention to treat. Long-term follow-up continues. The CHHiP trial is registered as an International Standard Randomised Controlled Trial, number ISRCTN97182923.

**Findings:**

Between Oct 18, 2002, and June 17, 2011, 3216 men were enrolled from 71 centres and randomly assigned (74 Gy group, 1065 patients; 60 Gy group, 1074 patients; 57 Gy group, 1077 patients). Median follow-up was 62·4 months (IQR 53·9–77·0). The proportion of patients who were biochemical or clinical failure free at 5 years was 88·3% (95% CI 86·0–90·2) in the 74 Gy group, 90·6% (88·5–92·3) in the 60 Gy group, and 85·9% (83·4–88·0) in the 57 Gy group. 60 Gy was non-inferior to 74 Gy (HR 0·84 [90% CI 0·68–1·03], p_NI_=0·0018) but non-inferiority could not be claimed for 57 Gy compared with 74 Gy (HR 1·20 [0·99–1·46], p_NI_=0·48). Long-term side-effects were similar in the hypofractionated groups compared with the conventional group. There were no significant differences in either the proportion or cumulative incidence of side-effects 5 years after treatment using three clinician-reported as well as patient-reported outcome measures. The estimated cumulative 5 year incidence of Radiation Therapy Oncology Group (RTOG) grade 2 or worse bowel and bladder adverse events was 13·7% (111 events) and 9·1% (66 events) in the 74 Gy group, 11·9% (105 events) and 11·7% (88 events) in the 60 Gy group, 11·3% (95 events) and 6·6% (57 events) in the 57 Gy group, respectively. No treatment-related deaths were reported.

**Interpretation:**

Hypofractionated radiotherapy using 60 Gy in 20 fractions is non-inferior to conventional fractionation using 74 Gy in 37 fractions and is recommended as a new standard of care for external-beam radiotherapy of localised prostate cancer.

**Funding:**

Cancer Research UK, Department of Health, and the National Institute for Health Research Cancer Research Network.

## Introduction

Prostate cancer is the most common cancer in men in the UK, with 41 736 new cases in 2011.^1^ Since the introduction of prostate-specific antigen (PSA) testing, most men diagnosed have localised disease. Management options include external-beam radiotherapy, brachytherapy, radical prostatectomy, active surveillance (for men with low-risk disease), and watchful waiting (for those unsuitable for radical curative treatment), with management choices often affected by potential treatment-related toxic effects. Prostate cancer and its treatment are the leading cause of cancer years lived with disability.^2^

**Research in context**

**Evidence before this study**

We searched PubMed for articles published between Jan 1, 1990, and Oct 18, 2002, before trial commencement using the terms “radiotherapy AND prostate cancer AND (hypofractionation OR alpha/beta ratio)” and then updated results to Sept 8, 2015. Before the CHHiP trial began, reports based on retrospective series of patients suggested that the α/β ratio for prostate cancer might be low, but only two small randomised trials had tested hypofractionation compared with conventional fractionation, both using relatively low doses of radiotherapy, and neither trial was large enough to confirm or refute a benefit. Since CHHiP started, more recent results from a meta-analysis of five small trials testing hypofractionation and retrospective reviews of large patient databases have been done, suggesting that the best estimates for the α/β ratio are between 1·4 Gy and 1·9 Gy, although estimates up to 8·3 Gy have been calculated. However, these retrospective analyses and reviews have not changed clinical practice; hence the need for a large randomised controlled trial. Meta-analyses of studies of dose-escalated radiotherapy and neoadjuvant androgen deprivation show improved disease control compared with standard radiotherapy doses, but dose escalation increases bowel side-effects. However, conformal and intensity-modulated radiotherapy improves dose distributions of radiotherapy and conformal radiotherapy reduces side-effects.

**Added value of this study**

The CHHiP trial is, to our knowledge, the largest randomised treatment study undertaken in localised prostate cancer. We tested two experimental hypofractionated radiotherapy schedules using 3 Gy per fraction to total doses of 60 Gy and 57 Gy compared with standard fractionation using 2 Gy per fraction to a total dose of 74 Gy. We have shown that the hypofractionated schedule of 60 Gy in 20 fractions is non-inferior to a standard schedule of 74 Gy in 37 fractions for the endpoint of biochemical and clinical disease control. Overall treatment time was reduced from 7·4 weeks to 4 weeks. 57 Gy in 19 fractions could not be claimed to be non-inferior to the control 74 Gy group. The results give an estimate of 1·8 Gy for the α/β ratio for prostate cancer. Quality controlled IMRT techniques were used and the side-effect profiles were favourable and low in all three randomised groups.

**Interpretation**

The findings from this pre-planned analysis of the CHHiP trial show that the hypofractionated IMRT schedule giving 60 Gy in 3 Gy fractions in 4 weeks is both effective and safe and can be recommended as a new standard of care for patients with localised prostate cancer using the high-quality radiotherapy techniques described. The results are most robust for patients with intermediate-risk disease who received short-course androgen deprivation therapy.

External-beam radiotherapy is most appropriate for men with intermediate-risk or high-risk disease,^3^ and is associated with long-term disease control in most patients.^4^ About 15 800 men receive radical prostate radiotherapy in the UK every year (Ball C, National Clinical Analysis and Specialised Applications Team, The Clatterbridge Cancer Centre NHS Foundation Trust, personal communication). Several phase 3 randomised controlled trials have shown the benefit of dose escalation^5^, ^6^ and high-dose conformal radiotherapy with conventional 2 Gy daily fractions to a total dose of 74 Gy is the standard of care in the UK.^7^ However, a meta-analysis showed that high-dose radiotherapy (74–80 Gy) is associated with an increased risk (odds ratio 1·58) of late gastrointestinal toxicity of grade 2 or more compared with lower doses of radiotherapy (64–70·2 Gy).^8^ Therefore, it is important to use advanced radiotherapy techniques that are able to sculpt dose distributions to the prostate target and avoid the organs at risk.

Additionally, there has been interest in the fraction sensitivity of prostate cancer.^9^, ^10^, ^11^ The association between total isoeffective radiation dose and fraction size is described by a linear quadratic model which uses two constants: α and β. The ratio α/β is inversely related to the effect of changes in fraction size on normal and malignant tissues. The α/β ratio for most cancers and acute normal tissue reactions is believed to be high and about 10 Gy. However, for prostate cancer, a value as low as 1·5 Gy has been suggested, which is lower than the 3 Gy reported for the late reactions of most normal tissues (including rectum).^12^ These findings have potentially important therapeutic implications. Hypofractionated radiotherapy, giving fewer fractions each with a higher dose, might improve the therapeutic ratio, resource use, and patient convenience.

The main aims of the CHHiP trial (CRUK/06/016) were to compare the efficacy and toxicity of conventional and hypofractionated radiotherapy using high-quality radiation techniques.

## Methods

### Study design and participants

CHHiP is an international, multicentre, randomised, phase 3, non-inferiority trial comparing the conventionally fractionated schedule of 74 Gy in 37 fractions with two experimental hypofractionated schedules of 60 Gy in 20 fractions and 57 Gy in 19 fractions in men with localised prostate cancer. Safety of the 3 Gy (fraction) schedules was reported after a pre-planned analysis of the first 457 men recruited.^13^ Here, we report primary efficacy results and further comparative safety data.

Men older than 16 years who had histologically confirmed T1b–T3aN0M0 prostate cancer and a WHO performance status of 0 or 1 were eligible. Initially, men with a PSA concentration of less than 40 ng/mL and risk of lymph node involvement^14^ less than 30% were eligible. On Aug 1, 2006, after 454 patients had been recruited, these criteria were revised to reflect the developing consensus on use of long-term androgen deprivation in locally advanced disease. Thereafter, a PSA concentration less than 30 ng/mL and a risk of seminal vesicle involvement^15^ less than 30% were needed. Patients were ineligible if they had both T3 tumours and a Gleason score of 8 or higher, or a life expectancy of less than 10 years. Other exclusion criteria included previous pelvic radiotherapy or radical prostatectomy, previous androgen suppression, another active malignancy in the past 5 years (other than cutaneous basal-cell carcinoma), comorbid conditions precluding radical radiotherapy, hip prosthesis (criterion amended to bilateral hip prosthesis Jan 30, 2009), and full anticoagulation treatment (criterion removed July 1, 2009). Full details of trial design, eligibility, and treatment have been reported previously.^13^ The protocol is available in the appendix (pp 27–90).

The study was approved in the UK by the London Multi-centre Research Ethics Committee (04/MRE02/10) and by the institutional research board of each participating international site. The trial was sponsored by the Institute of Cancer Research and was done in accordance with the principles of good clinical practice. All patients provided written informed consent. The Institute of Cancer Research Clinical Trials and Statistics Unit (ICR-CTSU; London, UK) coordinated the study and carried out central statistical data monitoring and all analyses. The trial management group was overseen by an independent trial steering committee.

### Randomisation and masking

Men were registered into the trial before or after commencement of androgen deprivation therapy. Following registration, and within 4–6 weeks before radiotherapy, patients were randomly assigned (1:1:1) to receive conventional fractionation (control) or one of two hypofractionated schedules. Randomisation was via telephone to the ICR-CTSU. Computer-generated random permuted blocks of sizes six and nine were used, stratified by National Comprehensive Cancer Network (NCCN) risk-classification (low *vs* intermediate *vs* high)^3^ and radiotherapy treatment centre. It was not possible to mask patients or clinicians to treatment allocation.

### Procedures

Short-course androgen deprivation treatment was given for 3–6 months before and during radiotherapy; this was optional for patients with low-risk disease. Injections of a luteinising-hormone-releasing hormone (LHRH) analogue every month, combined with initial anti-androgen to reduce testosterone flare, or an anti-androgen alone, were allowed. Individuals assigned to the 74 Gy in 37 fractions control group received 2 Gy daily fractions (Monday to Friday treatment) for 7·4 weeks. Individuals in the experimental groups received hypofractionated treatment with 3 Gy daily fractions to a total dose of either 60 Gy in 20 fractions in 4·0 weeks (≥28 days) or 57 Gy in 19 fractions in 3·8 weeks (≥27 days). Biological doses in the hypofractionated schedules were calculated to be equivalent to those in the conventional schedule assuming α/β ratios of 2·4 Gy for the 60 Gy group and 1·4 Gy for the 57 Gy group. All treatment groups received intensity-modulated radiation techniques (IMRT). Treatment delays for toxic effects, and for technical reasons of up to 5 days, were permitted.

Planning of radiotherapy treatment for all three groups was done with forward or inverse three-dimensional methods about 12 weeks after the start of hormonal treatment. The complex forward-planned multisegment technique using an integrated simultaneous boost has been previously described^16^ using three treatment fields with a total of eight segments. Pelvic lymph nodes were not included in the target volumes. Mandatory dose constraints were defined for target coverage and avoidance of normal tissues including rectum, bowel, bladder, and femoral heads. Treatment plans were reviewed and dose reductions permitted to meet dose constraints. Treatment was delivered with 6–15 MV photons with multileaf collimators to shape beams. Portal imaging was used to verify treatment accuracy, which was to be within 3 mm and was taken at least three times during week 1 and at least weekly intervals thereafter. Use of image-guided techniques (IGRT) was permitted but not required. Details of target volumes, dose parameters, and constraints are given in the appendix (pp 2, 3, 15). The integral quality-assurance programme has previously been described.^13^

Staging investigations included PSA measurement, standard haematology and biochemistry, lymph node assessment by pelvic MRI or CT, and bone scans for patients at intermediate or high risk. Histology was locally assessed with diagnostic transrectal ultrasound-guided biopsies (or specimens from transurethral resection of the prostate) and reported with the Gleason system. PSA concentrations were recorded before commencement of androgen deprivation therapy and radiotherapy and subsequently at weeks 10, 18, and 26 after radiotherapy and then at 6-month intervals for 5 years and subsequently annually.

Baseline, pre-radiotherapy treatment, acute, and late toxicity data were collected using physician-completed and patient-reported outcome questionnaires. Instruments chosen reflected practice at the time of trial commencement, the desirability of assessing symptoms before radiotherapy to allow consideration of emergent events, and to facilitate comparison with other studies. Baseline bowel, bladder, and sexual function assessments were made before androgen deprivation therapy and radiotherapy and were graded using the Late Effects on Normal Tissues: Subjective/Objective/Management (LENT/SOM)^17^ and Royal Marsden Hospital (RMH)^18^ scoring systems and patient-reported outcome questionnaires. Acute toxicity data were collected for the first 2163 randomly assigned patients. When the sample size was increased (see Statistical analysis) it was felt that sufficient data had been collected on acute toxicity to allow robust conclusions to be drawn about comparisons between the three randomised groups. Reactions were graded every week during radiotherapy and at weeks 10, 12, and 18 from radiotherapy start date using the Radiation Therapy Oncology Group (RTOG) scoring system for acute toxicity.^19^ Late side-effects were then assessed beginning 26 weeks after the start of radiotherapy and every 6 months for 2 years and then yearly to 5 years, as previously described,^13^ using the RTOG grades for late side-effects,^19^ RMH, and LENT/SOM scoring systems. A quality-of-life substudy using patient-reported outcomes was included as previously described.^20^ From trial initiation to early 2009, the UCLA-PCI, including the Short Form 36 (SF-36), and the Functional Assessment of Cancer Therapy-Prostate (FACT-P) quality-of-life instruments were used. After a protocol amendment on March 12, 2009, the Expanded Prostate Cancer Index Composite (EPIC) and Short Form 12 (SF-12) instruments replaced UCLA-PCI, SF-36, and FACT-P due to EPIC becoming the patient-reported outcome measure of choice. EPIC-50 was used for bowel and urinary domains and EPIC-26 for sexual and hormonal domains. Patient-reported outcomes to 2 years after treatment have been reported.^20^

### Outcomes

The primary outcome measure was time to biochemical or clinical failure, defined as the time from randomisation to biochemical failure or prostate cancer recurrence. The initial definition of biochemical failure (PSA >2 ng/mL 6 months or more after the commencement of radiotherapy and a PSA rising by 50% or more from the nadir) was updated in 2007, and applied retrospectively, to reflect the Phoenix consensus guidelines as a PSA concentration greater than nadir plus 2 ng/mL.^21^ The nadir PSA was the lowest concentration recorded at any time after commencement of androgen deprivation therapy or radiotherapy. A consecutive confirmatory PSA concentration was required. Biochemical failure events were determined centrally from PSA concentrations and confirmed by the local investigator. Prostate cancer recurrence events were as reported by the investigator and included recommencement of androgen deprivation therapy, local recurrence, lymph node or pelvic recurrence, and distant metastases.

Secondary efficacy outcome measures were disease-free survival (time from randomisation to any prostate cancer-related event or death from any cause); overall survival (time from randomisation to death from any cause); development of metastases; and recommencement of hormonal treatment for disease recurrence. Cause of death was centrally reviewed by a panel of three trial investigators (DD, JG, IS), masked to treatment allocation.

Additional secondary endpoints were acute and late side-effects. Acute toxicity outcomes were summarised by reporting the peak and week 18 bowel and bladder side-effects. Clinician-reported late toxicity outcomes were the proportion of patients with a grade 2 or worse toxic effect at 2 and 5 years, and time to development of grade 1, grade 2, and grade 3 toxicity (assessed using each scoring method). Patient-reported outcomes included overall bowel, bladder, and sexual dysfunction bother reported as single items on the UCLA-PCI and EPIC-50 instruments.

### Statistical analysis

The trial was powered to assess non-inferiority in biochemical or clinical failure-free rate between the hypofractionated and conventional radiotherapy schedules. A three-arm design allowed estimation of isoeffective doses for both efficacy and complications. We assumed a 70% failure-free rate at 5 years in the control group and, with 2163 patients, initially wished to exclude a decrease of 6% in a hypofractionated group. Due to accrual exceeding expectations, a protocol amendment on Nov 23, 2009, increased the sample size to allow a smaller non-inferiority margin of 5%, corresponding to a critical hazard ratio (HR) of 1·208, to be used. This critical HR was used for all non-inferiority analyses. To conclude non-inferiority with 80% power (one-sided α=0·05), 3163 men (1054 per treatment group) were required. This analysis would require 349 events in the control group but, as agreed with the Independent Data Monitoring Committee, data could also be considered sufficiently mature for analysis after a median follow-up of 5 years. A small allowance (1·5%) for dropout or loss to follow-up was incorporated.

Analyses for all time-to-event endpoints were on an intention-to-treat basis. The primary outcome was also analysed in the per-protocol population, including all patients receiving at least one fraction of their allocated radiotherapy schedule. For time to biochemical or clinical failure, patients event free at the time of analysis were censored at their last known PSA assessment. For disease-free and overall survival, patients were censored at the date they were last known to be alive. For development of metastases and recommencement of hormonal treatment patients were censored at the date they were last seen or date of death.

Kaplan-Meier methods were used to estimate event rates. Estimates of treatment effect were made using unadjusted and also adjusted Cox regression models, with an HR less than 1 indicating a decreased risk of the event in the hypofractionated treatment group compared with control. Covariates included in adjusted Cox regression models were age (≤69 years *vs* >69 years), NCCN risk group (low *vs* intermediate *vs* high), Gleason score (≤6 *vs* >6), clinical stage, and pre-androgen deprivation therapy PSA (<10 ng/mL *vs* 10–20 ng/mL *vs* >20 ng/mL). Although the trial was not designed to directly compare the hypofractionated schedules, hypothesis generating comparisons have been made, with an HR less than 1 indicating a decreased risk of the event in the 60 Gy group compared with the 57 Gy group. For time to biochemical or clinical failure, HRs are provided with two-sided 90% CIs (equivalent to one-sided 95% CIs) in accordance with the one-sided non-inferiority design. p values to reject the null hypothesis of HR of 1·208 or greater (p_NI_) are reported. In all other instances, 95% CIs are reported. Comparisons were made between the control group and each hypofractionated group using the log-rank test, with a p value less than 0·05 indicating statistical significance.

Absolute treatment differences (δ) in time to biochemical or clinical failure have been calculated based on the Kaplan-Meier estimate of the failure-free rate in the control group and the HR. A competing risks analysis was done using the methods of Fine and Gray for the primary outcome measure, with death due to any cause as the competing event with consistent results (data not shown). Pre-planned subgroup analyses of the primary outcome by NCCN risk group were done in addition to multivariable analyses adjusting for risk group and prespecified clinically prognostic factors. Heterogeneity of the treatment effect was tested using χ^2^ tests for interaction. The α/β ratio for prostate cancer was estimated^10^ assuming a linear fit for data from the hypofractionated groups.

Acute toxicity analyses were done in the safety population, including all patients who received at least one fraction of radiotherapy. All available data were used irrespective of timing of assessment, with the exception of comparisons at 18 weeks, where a 2 week window either side of the expected date was used. Pairwise comparisons of the distribution of acute toxicity scores were compared using Mann-Whitney tests. For each late toxicity scale, the proportion of late grade 2 or worse toxicity at 2 years and 5 years is reported with exact binomial 95% CIs. Fisher's exact tests were used to compare each hypofractionated group with control. Time to first late adverse event was compared with the use of the Kaplan-Meier method. Patients not experiencing an event were censored at last known toxicity assessment. The proportion of small or worse patient-reported bother and time to first very small, small, moderate, or worse late bother score were analysed as for late toxicity endpoints. To make some allowance for multiple testing of toxicity and patient-reported endpoints, a p value of less than 0·01 was considered statistically significant.

For all time-to-event analyses the proportional hazards assumption of the Cox model was tested using Schoenfeld residuals and found to hold (appendix pp 25–26).

Analyses were based on a database snapshot taken on Sept 8, 2015, and were done using Stata version 13. The CHHiP trial is registered as an International Standard Randomised Controlled Trial, number ISRCTN97182923.

### Role of the funding source

The funding source provided peer-reviewed approval for the trial, but had no other role in study design, collection, analysis, interpretation of data, or writing of the report. The corresponding author had full access to all the data in the study and had final responsibility for the decision to submit for publication. HMo, CG, and EH also had full access to the data.

## Results

**Figure 1:**
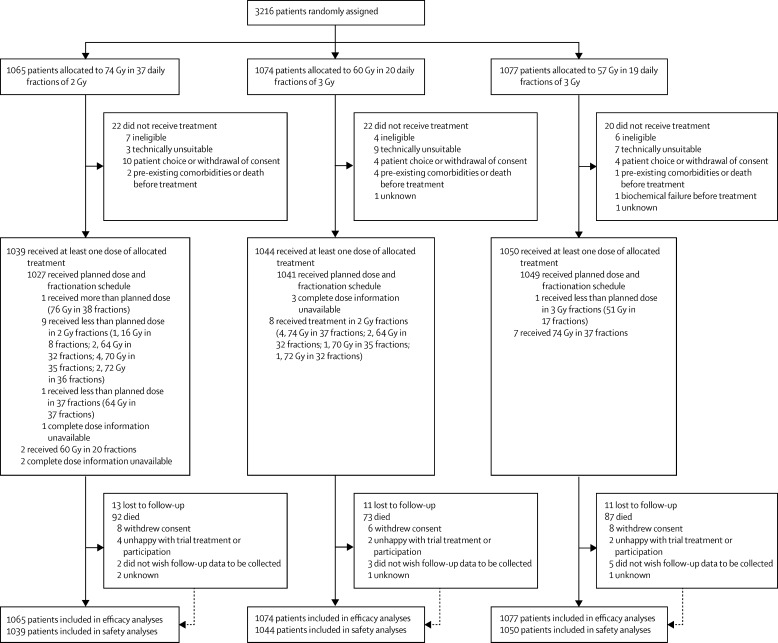
Trial profile

Between Oct 18, 2002, and June 17, 2011, 3216 men were recruited from 71 centres in the UK, Republic of Ireland, Switzerland, and New Zealand (appendix pp 1, 4–5). 1065 patients were assigned to the conventional 74 Gy schedule, 1074 to the 60 Gy schedule, and 1077 to the 57 Gy schedule; 64 patients received no radiotherapy (figure 1). Demographic and clinical characteristics were balanced across treatment groups (table). 3213 (97%) patients received concurrent androgen deprivation therapy, with 2700 (84%) patients receiving LHRH analogues and short-term anti-androgens. Most patients who did not receive androgen deprivation therapy had low-risk disease (73 [78%] of 93 patients). For patients who received androgen deprivation treatment, the median duration before commencement of radiotherapy was 16 weeks (IQR 15–20). Overall, 3152 (98%) patients received radiotherapy, and 3117 (97%) received the allocated dose and fractionation schedule (figure 1). Treatment delays of 1 week or more occurred in only 23 (1%) patients. At the time of analysis, median follow-up was 62·6 months (54·1–77·2) in the 74 Gy group, 62·2 months (53·9–77·2) in the 60 Gy group, and 62·4 months (53·7–76·6) in the 57 Gy group.

**Figure 2:**
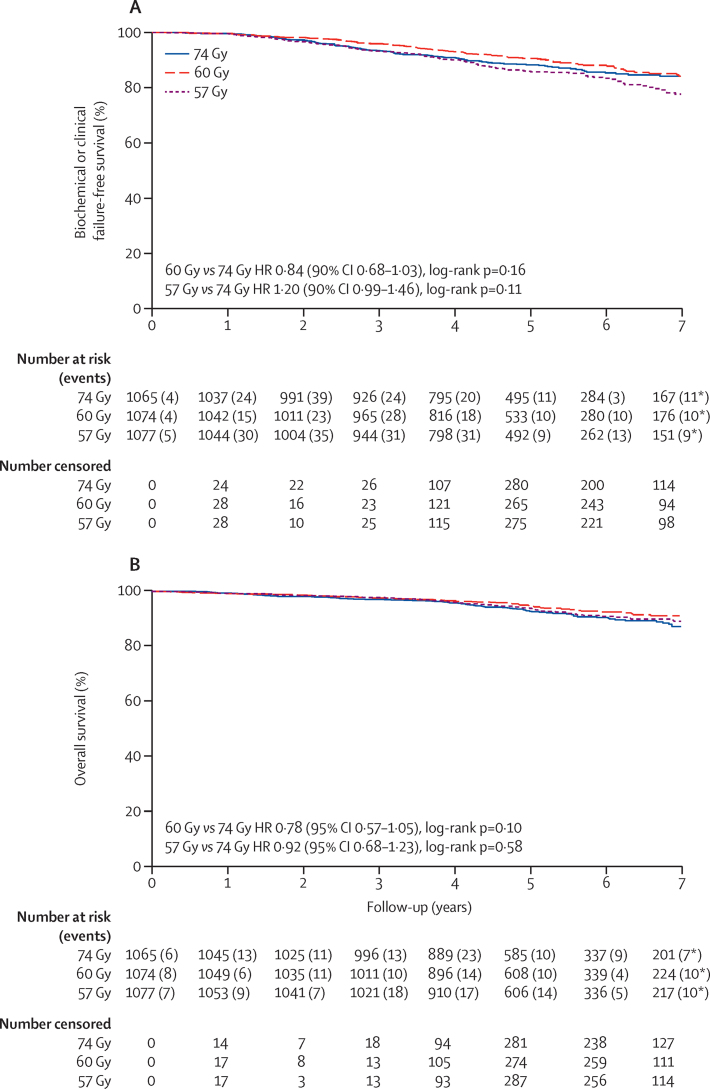
Biochemical or clinical failure-free survival (A) and overall survival (B) *Number of events reported after 7 years.

By 5 years, the number of patients with biochemical or clinical events were 111 of 1065 in the 74 Gy group, 88 of 1074 in the 60 Gy group, and 132 of 1077 in the 57 Gy group, respectively. 5-year biochemical or clinical failure-free rates were 88·3% (95% CI 86·0–90·2) in the 74 Gy group, 90·6% (88·5–92·3) in the 60 Gy group, and 85·9% (83·4–88·0) in the 57 Gy group (figure 2A, appendix p 6). With reference to the critical HR for non-inferiority, 60 Gy was non-inferior to 74 Gy with HR 0·84 (90% CI 0·68–1·03), p_NI_=0·0018. Since the upper limit of the 90% CI for the HR comparing 57 Gy with 74 Gy (HR 1·20 [0·99–1·46]) exceeds 1·208 (p_NI_=0·48), non-inferiority of the 57 Gy schedule relative to 74 Gy cannot be claimed. To facilitate comparison with other studies, 95% CIs were estimated as 0·65–1·07 for the HR in the 60 Gy group and 0·96–1·51 for the HR in the 57 Gy group. The estimated absolute difference in the proportion of patients in the hypofractionated groups free from biochemical or clinical failure compared with that in the control group at 5 years is δ=1·80% (90% CI −0·34 to 3·58) for 60 Gy versus 74 Gy and δ=–2·20% (–4·88 to 0·08) for 57 Gy versus 74 Gy. Analyses in the per-protocol population confirmed these results (60 Gy, HR 0·83 [90% CI 0·68–1·02], p_NI_=0·0015, δ=1·88% [90% CI −0·27 to 3·67]; 57 Gy, HR 1·17 [0·97 to 1·42], p_NI_=0·40, δ=–1·92% [–4·59 to 0·34]).

**Figure 3:**
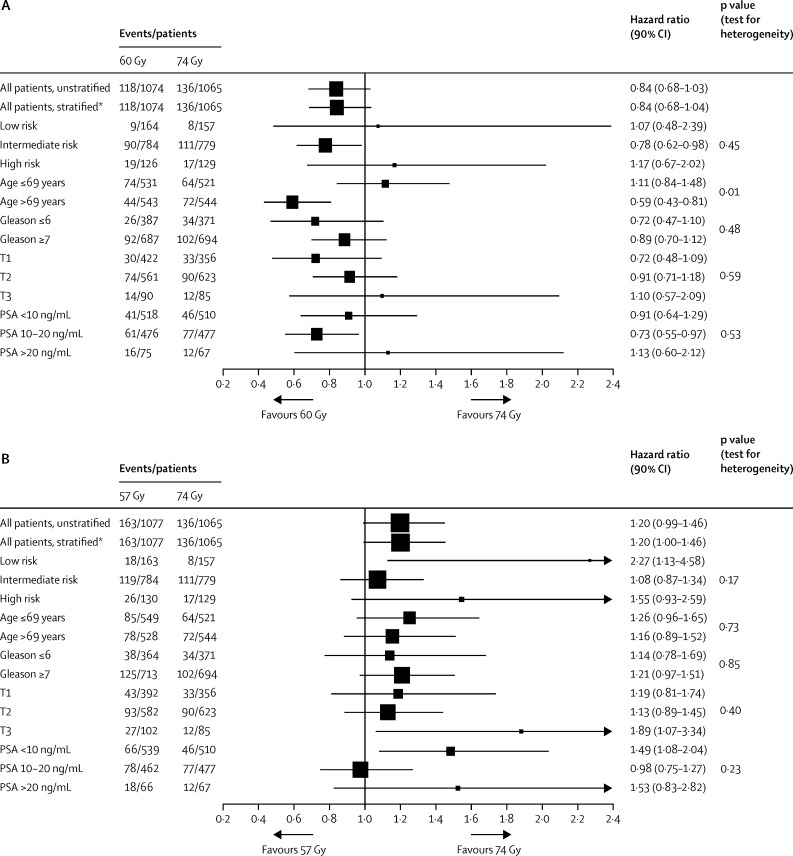
Univariable subgroup analyses of biochemical or clinical failure comparing 60 Gy (A) and 57 Gy (B) with conventional radiotherapy *Stratified by risk group; all other analyses are unstratified.

Estimates of the HR adjusted for age (≤69 years *vs* >69 years), NCCN risk group, Gleason score (≤6 *vs* ≥7), clinical stage, and pre-androgen deprivation therapy PSA (<10 ng/mL *vs* 10–20 ng/mL *vs* >20 ng/mL) also confirmed these results (60 Gy *vs* 74 Gy, HR 0·86 [90% CI 0·70–1·06], p=0·25; 57 Gy *vs* 74 Gy, HR 1·21 [90% CI 0·99–1·46], p=0·11; appendix p 7). Prespecified subgroup analyses of time to biochemical or clinical failure showed no significant interactions with treatment group, except for age, where older men (age >69 years) had a reduced biochemical or clinical failure rate with 60 Gy compared with 74 Gy, but younger men (age ≤69 years) showed no difference in biochemical or clinical failure rate between treatment groups; however, this difference was not seen for the 57 Gy group (figure 3). In an exploratory secondary analysis to compare 60 Gy in 20 fractions with 57 Gy in 19 fractions the HR was 0·70 (95% CI 0·55–0·88, log-rank p=0·0026; δ=4·07% [95% CI 1·56–6·10]; appendix p 6).

At 5 years, biochemical and clinical failure-free rates for the NCCN low-risk, intermediate-risk, and high-risk groups were: 96·7% (95% CI 92·3–98·6), 86·8% (84·0–89·1), and 86·5% (78·4–91·7) for the 74 Gy group; 96·6% (92·1–98·6), 90·2% (87·7–92·3), and 84·2% (75·7–90·0) for the 60 Gy group; and 90·9% (85·1–94·5), 86·0% (83·1–88·5), and 78·3% (69·2–85·0) for the 57 Gy group, respectively (appendix p 16).

92 (9%) deaths were reported in the 74 Gy group, 73 (7%) in the 60 Gy group, and 87 (8%) in the 57 Gy group. Of 252 deaths reported, 40 (16%) were prostate cancer related, 88 (35%) were due to a second malignancy, 111 (44%) were non-cancer causes, and 13 (5%) were of unknown cause. No significant differences in overall survival were observed between the control group and either of the hypofractionated groups (figure 2B; appendix p 6).

Progression events or death occurred in 209 (20%) of 1065 patients in the 74 Gy group, 179 (17%) of 1074 in the 60 Gy group, and 227 (21%) of 1077 in the 57 Gy group. Disease-free survival at 5 years was 82·3% (95% CI 79·6–84·6) in the 74 Gy group, 85·3% (82·8–87·5) in the 60 Gy group, and 80·1% (77·3–82·6) in the 57 Gy group. Compared with 74 Gy, the HR for disease-free survival was 0·83 (95% CI 0·68–1·01) in the 60 Gy group and 1·08 (0·90–1·31) in the 57 Gy group. 103 (3%) patients developed distant metastases: 32 (3%) in the 74 Gy group, 29 (3%) in the 60 Gy group, and 42 (4%) in the 57 Gy group. Androgen deprivation therapy was recommenced in 80 (8%) patients in the 74 Gy group, 70 (7%) in the 60 Gy group, and 89 (8%) in the 57 Gy group. Time to recommencement of androgen deprivation therapy and development of distant metastases were not significantly different between either of the hypofractionated schedules and the 74 Gy schedule (appendix pp 6, 17).

**Figure 4:**
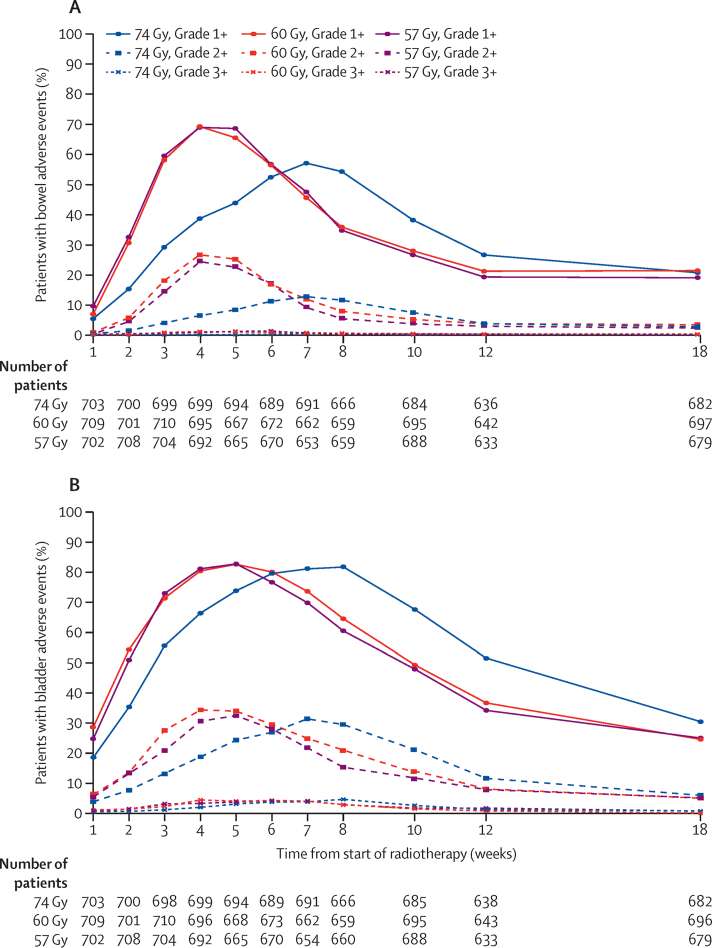
Acute RTOG toxicity by timepoint and randomised treatment group (A) Prevalence of bowel toxicity and (B) prevalence of bladder toxicity. RTOG=Radiation Therapy Oncology Group. Grade 1+=grade 1 or worse adverse event. Grade 2+=grade 2 or worse adverse event. Grade 3+=grade 3 or worse adverse event.

Acute RTOG bowel and bladder symptoms peaked sooner in the hypofractionated schedules than in the control, at 4–5 weeks compared with 7–8 weeks (figure 4, appendix p 18). There was significantly more acute bowel toxicity at the peak in the hypofractionated schedules compared with the control; the proportion of patients reporting RTOG grade 2 or worse bowel toxicity was 176 (25%) of 715 patients with available assessments in the 74 Gy group, 277 (38%) of 720 in the 60 Gy group (*vs* 74 Gy, p<0·0001), and 270 (38%) of 713 in the 57 Gy group (*vs* 74 Gy, p<0·0001; figure 4A). However, the distribution of bladder toxicity by grade was similar across all groups; the proportion of patients with available assessments reporting RTOG grade 2 or worse bladder toxicity was 331 (46%) of 715 in the 74 Gy group compared with 356 (49%) of 720 in the 60 Gy group (p=0·34), and 327 (46%) of 713 in the 57 Gy group (p=0·90; figure 4B). By 18 weeks, both bowel and bladder toxicity by RTOG assessment were similar between treatment groups (figure 4). Of 592 patients treated with 74 Gy with available assessments, 15 (3%) and 34 (6%) reported RTOG grade 2 or worse bowel and bladder toxicity, respectively. Corresponding proportions in the 60 Gy group (607 patients treated with available assessments) were 20 (3%) bowel (p=0·38) and 30 (5%) bladder (p=1·00), and in the 57 Gy group (508 patients treated with available assessments) were 15 (3%) bowel (p=0·60) and 30 (5%) bladder (p=0·10).

**Figure 5:**
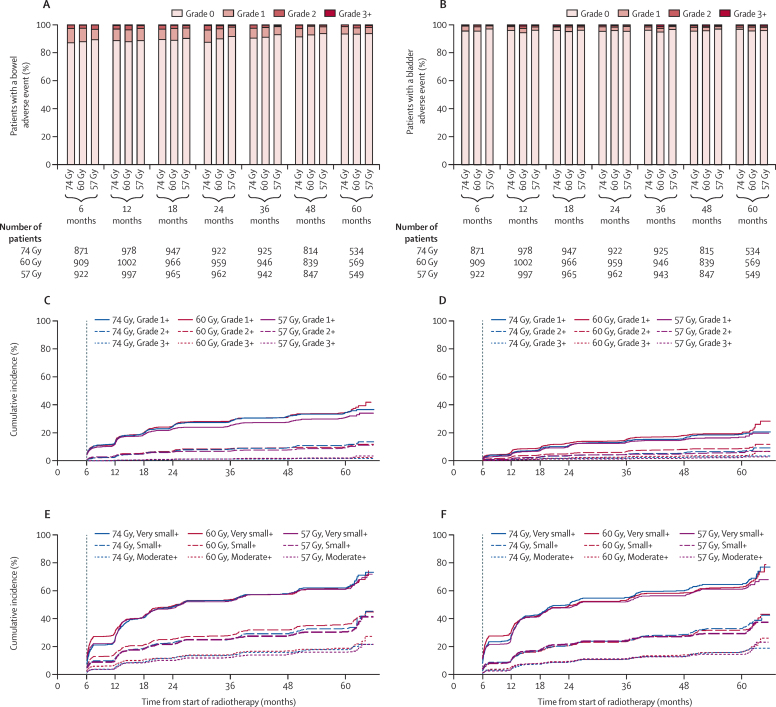
Late bowel and bladder toxicity by timepoint, assessment, and randomised treatment group Grade distribution of (A) bowel adverse events and (B) bladder adverse events measured with RTOG. Cumulative incidence of (C) bowel adverse events measured with RTOG and (E) bowel symptom scores measured with UCLA PCI/EPIC. Cumulative incidence of (D) bladder adverse events measured with RTOG and (F) bladder symptom scores measured with UCLA PCI/EPIC. Late toxicity data have been included in analyses if they were reported within 6 weeks of the 6 month visit, within 3 months of the 12–24 month visit, and within 6 months of the 36–60 month visit. For UCLA/EPIC, before androgen deprivation therapy data were included if they were reported within 3 months before starting androgen deprivation therapy and within 1 month after starting androgen deprivation therapy. Before radiotherapy data are included if they were reported within 3 months before radiotherapy and no more than 7 days after starting radiotherapy. Time-to-event analyses use all data reported from 6 weeks before the 6 month visit onwards. RTOG=Radiation Therapy Oncology Group scale. UCLA PCI=UCLA Prostate Cancer Index. EPIC=Expanded Prostate Cancer Index Composite. Grade 1+=grade 1 or worse adverse event. Grade 2+=grade 2 or worse adverse event. Grade 3+=grade 3 or worse adverse event. Very small+=score of very small, small, moderate, or big bother. Small+=score of small, moderate, or big bother. Moderate+=score of moderate or worse bother.

All radiotherapy schedules showed a low frequency of late bowel and bladder side-effects (figure 5, appendix pp 19–22). 2 years from the start of treatment fewer assessable patients treated with 57 Gy reported RTOG grade 2 or worse bowel symptoms as compared with 74 Gy control (74 Gy, 35 [4%] of 922 *vs* 57 Gy, 17 [2%] of 962; p=0·0075); however, no difference was observed between the 60 Gy group (28 [3%] of 959) and the 74 Gy group (p=0·31); this was evident across all clinician-reported toxicity scales (RTOG, RMH, and LENT-SOM; figure 5A, appendix pp 8, 19–20). The proportion of RTOG grade 2 or worse bladder symptoms at 2 years was similar across all treatment groups (74 Gy, 13 [1%] of 922; 60 Gy, 16 [2%] of 959 [*vs* 74 Gy, p=0·71]; 57 Gy, 11 [1%] of 962 [*vs* 74 Gy, p=0·68]), with no significant difference observed between control and either hypofractionated group on any scale (figure 5B, appendix pp 9, 21–22). Sexual dysfunction was common at baseline and increased with androgen deprivation therapy; although this partially recovered after radiotherapy it remained higher than baseline in all groups (appendix pp 10, 13, 23–24). The proportion of LENT-SOM grade 2 or worse sexual symptoms at 2 years was similar in each treatment group (74 Gy, 550 [67%] of 826 assessable patients; 60 Gy, 562 [65%] of 864 [*vs* 74 Gy, p=0·54]; 57 Gy, 552 [64%] of 859, [*vs* 74 Gy, p=0·33).

At 5 years post-radiotherapy, the frequency of grade 2 or worse bowel, bladder, and sexual toxicity across clinician-reported toxicity scales was similar across fractionation schedules (appendix pp 8–10). We identified no significant differences in the incidence of late grade 1 or worse, grade 2 or worse, or grade 3 or worse bowel, bladder, or sexual symptoms in either hypofractionated group compared with control at any timepoint using any clinician-reported toxicity scale (appendix pp 8–13). Estimated cumulative incidences of grade 2 or worse bowel toxicity at 5 years measured with the RTOG scale were 13·7% (111 events) for the 74 Gy group, 11·9% (105 events) for the 60 Gy group (HR compared with 74 Gy 0·94 [95% CI 0·72–1·23], p=0·65) and 11·3% (95 events) for the 57 Gy group (HR compared with 74 Gy 0·84 [0·64–1·11], p=0·22). Estimated cumulative incidences of grade 2 or worse bladder toxicity at 5 years measured with the RTOG scale were 9·1% (66 events) for the 74 Gy group, 11·7% (88 events) for the 60 Gy group (HR compared with 74 Gy 1·34 [95% CI 0·98–1·85], p=0·07) and 6·6% (57 events) for the 57 Gy group (HR 0·85 [0·60–1·21], p=0·37; figure 5, appendix p 9). There was a slightly higher frequency of grade 2 or worse bowel and bladder side-effects in the 60 Gy group compared with 57 Gy at 2 and 5 years on most clinician-reported scales (appendix pp 8–9). Cumulative incidence of LENT-SOM grade 2 or worse bowel side-effects was higher in the 60 Gy group compared with the 57 Gy group (HR 1·39 [95% CI 1·14–1·70], p=0·0010; appendix p 8), but this difference was not seen with other toxicity scales. Cumulative incidence of grade 2 or worse RTOG bladder toxicity was also greater in the 60 Gy group versus the 57 Gy group (HR 1·58 [1·13–2·20], p=0·0073; figure 5, appendix p 9), although this was not seen with other toxicity scales. More serious grade 3 or worse toxicities were rare in all groups (figure 5). At 5 years, the proportions of patients with RTOG grade 3 or worse bowel events were none of 534 patients in the 74 Gy group, two (<1%) of 569 patients in the 60 Gy group, and three of 549 (<1%) in the 57 Gy group; RTOG grade 3 or worse bladder events occurred in two (<1%) of 534 patients in the 74 Gy group, four (<1%) of 569 patients in the 60 Gy group, and five (1%) of 549 patients in the 57 Gy group. The estimated cumulative incidence of grade 3 or worse bowel and bladder adverse events at 5 years was 2% (17 events) and 3% (27 events) in the 74 Gy group, 3% (20 events) and 6% (38 events) in the 60 Gy group, and 4% (23 events) and 3% (21 events) in the 57 Gy group, respectively (figure 5C, D). No treatment-related deaths were reported.

Patient-reported outcomes showed no significant differences in the proportion of small or worse bowel, bladder, or sexual bother at 2 or 5 years (figure 5, appendix pp 8–13). At 5 years, the proportion of assessable patients reporting small or worse bowel bother was 49 (14%) of 341 in the 74 Gy group, 57 (15%) of 375 in the 60 Gy group, and 59 (15%) of 387 in the 57 Gy group. The corresponding figures for bladder bother were 56 (17%) of 333, 63 (17%) of 371, and 60 (16%) of 376, respectively, and for sexual bother were 187 (52%) of 357, 184 (52%) of 357 and 195 (53%) of 370, respectively. The cumulative incidence of late small or worse bother in each hypofractionated schedule was not significantly different from control for bowel, bladder, and sexual symptoms, and there were no significant differences between the hypofractionated groups (figure 5E, F, appendix pp 8–10).

## Discussion

In this pre-planned analysis with a median follow-up of over 5 years, we found that the 60 Gy hypofractionated schedule is non-inferior to the 74 Gy conventionally fractionated schedule in terms of time to biochemical or clinical failure for patients with localised prostate cancer. Evaluation of the lower 57 Gy hypofractionated schedule was inconclusive: it cannot be stated to be non-inferior to the 74 Gy control group but it was inferior to the 60 Gy group. Notably, the proportion of patients free from biochemical or clinical failure at 5 years in all treatment groups (88·3% for the 74 Gy group, 90·6% for the 60 Gy group, and 85·9% for the 57 Gy group) were considerably higher than the 71% reported for the 74 Gy group in the national MRC RT01 trial.^6^ Recent meta-analyses^5^, ^22^ identified five relatively small phase 3 trials comparing modest hypofractionation with conventional 1·8–2·0 Gy fractions. A total of 1825 patients were included, although 1153 received relatively low doses of radiotherapy (≤67 Gy in the control groups). There were no consistent differences between randomised groups, and the investigators concluded that larger trials were required to establish the non-inferiority of hypofractionation for clinical effectiveness. In the CHHiP trial, we estimate the α/β ratio to be 1·8 Gy, which is in keeping with previous reports^23^ and recent large series and meta-analyses which have suggested the α/β ratio to be between 1·4 Gy and 1·93 Gy.^24^, ^25^, ^26^, ^27^ Our estimate does not account for any time factor related to overall treatment duration and the potential effect of accelerated repopulation.^26^ The improvement in 5-year disease control for a 3 Gy dose difference between the 57 Gy and 60 Gy groups is in keeping with the review of the six randomised controlled dose-escalation trials previously reported^5^, ^6^ and a recent meta-analysis of biologically equivalent dose escalation.^28^

Five other contemporary phase 3 studies have reported side-effects related to hypofractionated radiotherapy^29^, ^30^, ^31^, ^32^, ^33^ (appendix p 14). We initially reported no differences between the three randomised groups in the frequency of side-effects in the first 457 patients recruited to the CHHiP trial with 2 years follow-up.^13^, ^20^ Analysis of acute side-effects in the full CHHiP cohort has confirmed no difference in bladder side-effects except that the wave of toxicity occurs earlier in the hypofractionated groups. However, we have now documented that the peak acute bowel toxicity is greater in patients receiving a hypofractionated schedule, although it is noteworthy that only 2% of patients had grade 3 or worse toxic effects and only 1% had a prolongation of treatment time of more than 1 week, indicating the tolerability of the hypofractionated schedules. An increased acute gastrointestinal reaction in patients treated with hypofractionated radiotherapy has been noted by other investigators^29^, ^32^ but not by Norkus and colleagues,^33^ who treated patients using a 4 day per week schedule. The different treatment schedules and total dose given might account for these differences (appendix p 14). There were no significant differences in late bowel toxicity between the 74 Gy and 60 Gy groups in any of the domains at any timepoint. The 57 Gy group had less grade 1 or worse and grade 2 or worse LENT-SOM bowel and grade 2 or worse RTOG bladder side-effects than the 60 Gy group with outcomes on other toxicity scales supporting this effect, although they were not significant; there were no significant differences between the 57 Gy and 60 Gy groups in sexual function domains. The results are in accord with a recent meta-analysis indicating the general tolerability of hypofractionated radiotherapy,^5^, ^22^ although two studies have reported approximate doublings of grade 2 gastrointestinal^31^ or genitourinary^30^ side-effects compared with conventional 1·8–2·0 Gy fractions (appendix p 14).

Post-radiotherapy symptoms relate to treatment methods, total dose, patient factors, and fractionation schedules. The favourable results in the CHHiP study reflect the mandated radiotherapy technique using an integrated simultaneous boost with relatively narrow planning target volume margins^16^ and normal tissue dose constraints. The cumulative rate of RTOG grade 2 or worse gastrointestinal side-effects observed by 5 years was 14% compared with 33% for the 74 Gy group in the RT01 trial,^34^ which used conformal radiotherapy without specified dose constraints. We previously reported complementary findings from the CHHiP trial showing a greater than 50% reduction in bowel bother or distress compared with the RT01 trial using the UCLA-PCI instrument.^20^ Additionally, the apparently more favourable bladder toxicity results reported in the CHHiP trial compared with other studies might relate to lower total delivered dose^29^, ^30^ or amount of bladder and trigone included in the high-dose volume,^31^ which would be in keeping with our observation of lower side-effects in the 57 Gy compared with 60 Gy hypofractioned groups. This finding suggests that a steep dose–volume association might exist for late bladder complications using hypofractionated schedules.

Strengths of the CHHiP trial include its size and multicentre recruitment. Consistent and quality assured radiotherapy delivery in 40 centres demonstrates the generalisability of the radiotherapy techniques. The study design, using two experimental hypofractionated groups, enables clarity of clinical interpretation. Limitations are that results are primarily applicable to patients receiving short-course androgen deprivation therapy (97% of the men treated) and might not be generalisable for populations who do not receive androgen deprivation therapy, and are most robust for patients with intermediate-risk disease (73% of the men treated), although there was no heterogeneity of effect across all risk groups. We have found a low level of side-effects in all groups. However, further follow-up is required to assess 10-year and 15-year outcomes, and it is possible that differences might yet emerge. Because the hypofractionated and conventional radiotherapy were given over different lengths of time, the study does not address the issue of treatment duration and accelerated repopulation in prostate cancer and our estimate of the α/β ratio of 1·8 Gy does not include a time factor.

Other complementary phase 3 studies treating patients with prostate cancer with radiotherapy alone without androgen deprivation therapy and using hypofractionated radiotherapy with different treatment durations will clarify these issues. In the Netherlands, the HYPRO study (ISRCTN85133859)^32^ randomised 820 patients with intermediate-risk or high-risk disease to high-dose conventional radiotherapy (78 Gy in 39 fractions) or dose-escalated hypofractionated radiotherapy of 64·6 Gy in 19 fractions given over a period of 6–7 weeks with or without androgen deprivation therapy. In Canada, the PROFIT trial (ISRCTN43853433) has randomised 1204 men with intermediate-risk disease to receive either 60 Gy in 20 fractions over 4 weeks or 78 Gy in 39 fractions. For the low-risk patient group, the RTOG 0415 study recruited 1115 patients who were randomly assigned to receive 70 Gy in 28 fractions over 5·5 weeks or 73·8 Gy in 41 fractions over 8·1 weeks. More extreme forms of hypofractionated radiotherapy are now being studied. The HYPO trial (ISRCTN45905321) will shortly complete recruitment of 1200 men comparing 43·7 Gy in seven fractions over 15–19 days with 78 Gy in 39 fractions over 7·8 weeks and the PACE trial (ISRCTN17627211) compares 36·25 Gy in five fractions over 1–2 weeks with 78 Gy in 39 fractions over 7·8 weeks. Both trials use IGRT, which permits reduced target margins around the prostate, and might reduce treatment side-effects. Outcome results will not be available for several years.

Prostate cancer radiotherapy accounts for 27% of the workload of radiotherapy departments in the UK.^35^ Radical external-beam radiotherapy was given to 14 364 patients in 2014–15 in England and Wales involving 455 638 attendances. Before commencement of the CHHiP trial, 3 Gy fraction schedules were rarely used, but by 2014–15, after publication of initial safety results,^13^ 19% of patients received 3 Gy hypofractionated schedules (Ball C, personal communication). A uniform change to a 20-fraction schedule could reduce the number of treatment fractions and attendances by over 200 000 annually in the UK. The CHHiP trial results show that the combination of hypofractionation and high-quality radiotherapy techniques gives excellent tumour control, a low level of side-effects, and increased convenience for patients compared with a conventional fractionation schedule. High-dose modest hypofractionation using high-quality treatment methods such as those used in this trial should become a new standard of care for external-beam radiotherapy. The trial results might act as a benchmark for comparison with other treatment approaches, including radical prostatectomy, brachytherapy, and external beam radiation therapy without androgen deprivation therapy or using extreme hypofractionation schedules.

**This online publication has been corrected. The corrected version first appeared at thelancet.com/oncology on June 24, 2016**

## Figures and Tables

**Table:** Baseline demographics, clinical characteristics, and treatment details by randomised group

		**74 Gy in 37 fractions (N=1065)**	**60 Gy in 20 fractions (N=1074)**	**57 Gy in 19 fractions (N=1077)**
Age (years; range)		69 (48–85)	69 (48–84)	69 (44–83)
NCCN risk group				
	Low risk	157 (15%)	164 (15%)	163 (15%)
	Intermediate risk	779 (73%)	784 (73%)	784 (73%)
	High risk	129 (12%)	126 (12%)	130 (12%)
Gleason score				
	≤6	371 (35%)	387 (36%)	364 (34%)
	7	656 (62%)	658 (61%)	681 (63%)
	8	38 (4%)	29 (3%)	32 (3%)
Clinical T stage				
	T1a–T1b–T1c–T1x	356 (33%)	422 (39%)	392 (36%)
	T2a–T2b–T2c–T2x	623 (58%)	561 (52%)	582 (54%)
	T3a–T3x	85 (8%)	90 (8%)	102 (9%)
	Missing, unknown, or not done	1 (<1%)	1 (<1%)	1 (<1%)
Pre-androgen deprivation therapy PSA (ng/mL)				
	Median	10 (7–14)	10 (7–15)	10 (9–14)
	Mean	11 (5)	11 (6)	11 (5)
	<10	510 (48%)	518 (48%)	539 (50%)
	10–20	477 (45%)	476 (44%)	462 (43%)
	≥20	67 (6%)	75 (7%)	66 (6%)
Comorbidity				
	Diabetes	107 (10%)	115 (11%)	120 (11%)
	Hypertension	400 (38%)	441 (41%)	435 (40%)
	Inflammatory bowel disease	41 (4%)	39 (4%)	44 (4%)
	Previous pelvic surgery	86 (8%)	88 (8%)	78 (7%)
	Symptomatic haemorrhoids	68 (6%)	78 (7%)	63 (6%)
	Previous TURP	82 (8%)	88 (8%)	89 (8%)
Intended androgen deprivation therapy				
	LHRH plus short-term AA	881 (83%)	910 (85%)	909 (84%)
	150 mg bicalutamide	144 (14%)	133 (12%)	126 (12%)
	Other	6 (1%)	1 (<1%)	2 (<1%)
	None	29 (3%)	27 (3%)	34 (3%)
Median duration of androgen deprivation therapy (weeks)*		25 (21–28)	24 (19–27)	23 (20–27)
Median time from start of androgen deprivation therapy to radiotherapy (weeks)†		16 (14–20)	16 (14–19)	16 (15–20)
Median time from randomisation to start of radiotherapy (weeks)‡		8 (5–11)	8 (5–11)	7 (5–11)
Median duration of radiotherapy (days)		53 (51–55)	29 (28–29)	28 (27–28)
Radiotherapy planning				
	Forward planned	626 (59%)	624 (58%)	626 (58%)
	Inverse planned	304 (29%)	337 (31%)	322 (30%)
	Unavailable	135 (13%)	113 (11%)	129 (12%)
Radiotherapy delivery				
	No image guidance	563 (53%)	568 (53%)	563 (52%)
	Image-guided	312 (29%)	322 (30%)	326 (30%)
	Unavailable	190 (18%)	184 (17%)	188 (17%)

Data are n (%), mean (SD), or median (IQR), unless otherwise stated. NCCN=National Comprehensive Cancer Network. PSA=prostate-specific antigen. TURP=transurethral resection of the prostate. LHRH=luteinising-hormone-releasing hormone. AA=anti-androgen.

* Data presented for patients who received androgen deprivation therapy and a start and end date of treatment is known (n=950 in the 74 Gy group, n=966 in the 60 Gy group, and n=970 in the 57 Gy group).

† Data presented for patients who received androgen deprivation therapy and started radiotherapy (n=1008 in the 74 Gy group, n=1022 in the 60 Gy group, and n=1020 in the 57 Gy group).

‡ Data presented for patients who started radiotherapy (n=1043 in the 74 Gy group, n=1051 in the 60 Gy group, and n=1056 in the 57 Gy group).
