# Correction to *Lancet Oncol* 2016; 17:1055

**DOI:** 10.1016/S1470-2045(16)30273-X

**Published:** 2016-08

*Dearnaley D, Syndikus I, Mossop H, et al. Conventional versus hypofractionated high-dose intensity-modulated radiotherapy for prostate cancer: 5-year outcomes of the randomised, non-inferiority, phase 3 CHHiP trial.* Lancet Oncol *2016; **17:** 1047–60*—In figure 4B of this Article, the curves for 57 Gy grade 1+ and 60 Gy grade 1+ were incorrect. The figure has been updated. These corrections have been made to the online version as of June 24, 2016, and the printed version is correct.

