# Supplementary material for: Conventional versus hypofractionated high-dose intensity-modulated radiotherapy for prostate cancer: 5-year outcomes of the randomised, non-inferiority, phase 3 CHHiP trial
Source: Lancet Oncol. 2016 Aug;17(8):1047–60. doi: 10.1016/S1470-2045(16)30102-4 (PMC4961874; doi:10.1016/S1470-2045(16)30102-4)
Supplement: Supplementary appendix [file mmc1.pdf]

THE LANCET Oncology

Supplementary appendix

This appendix formed part of the original submission and has been peer reviewed. We post it as supplied by the authors.

Supplement to: Dearnaley D, Syndikus I, Mossop H, et al, on behalf of the CHHiP Investigators. Conventional versus hypofractionated high-dose intensity-modulated radiotherapy for prostate cancer: 5-year outcomes of the randomised, non-inferiority, phase 3 CHHiP trial. *Lancet Oncol* 2016; published online June 20. http://dx.doi.org/10.1016/S1470-2045(16)30102-4.

Web appendix

The CHHIP centres

Principal and main co-investigators according to centre (number of patients recruited in bold).
† = CHHIP Trial Management Group member

Addenbrooke's Hospital, **100**, Dr H Patterson†, Dr Y Rimmer; Alexandra Hospital, Redditch,**23,** Dr J Hamilton; Auckland Hospital, New Zealand, **10**, Dr C Jose; Ayr Hospital, **46**, Dr R Mahmood, Dr J Ansari, Dr H Glen; Basingstoke and North Hampshire Hospital, **22**, Dr R Shaffer; Beatson Oncology Centre, **62**, Dr J Graham, Dr M Russell, Dr J Wallace; Bedford Hospital, **61**, Dr R Thomas; Belfast City Hospital, **92**, Dr J O'Sullivan; Bradford Royal Infirmary, **14**, Dr A Henry; Bristol Haematology and Oncology Centre, **26**, Dr M Beresford, Dr A Bahl; Burnley General Hospital, **5**, Dr O Parikh; Charing Cross Hospital, **9**, Dr S Mangar; Cheltenham General Hospital, **5**, Dr P Jenkins; Christie Hospital, Manchester, **102**, Dr J Logue, Dr A Choudhury†; Clatterbridge Centre for Oncology, **177**, Dr I Syndikus†; Clayton Hospital, Wakefield, **8**, Dr A Henry; Countess of Chester Hospital, **33**, Dr A Ibrahim; Croydon University Hospital, 28, Dr R Huddart; Dorset County Hospital, **1**, Dr P Crellin; Eastbourne District General Hospital **29**, Dr F McKinna; Freeman Hospital, Newcastle, **29**, Dr I Pedley; Good Hope Hospital, **3**, Dr J Glaholm, Dr D Ford; Halton Hospital, Runcorn, **9**, Dr I Syndikus†; Hammersmith Hospital, **37**, Dr S Mangar; Heartlands Hospital, Birmingham, **24**, Dr A Zarkar; Hereford County Hospital, **19**,Dr A Cook; Ipswich Hospital, **153**, Dr C Scrase†; James Paget Hospital, Great Yarmouth,**8**, Dr R Wade; Kings Lynn and Wisbech NHS Hospital, **57**, Dr G Horan; Lincoln County Hospital, **155**, Dr M Panades; Maidstone Hospital, **6**, Dr S Beesley; Mount Vernon Hospital, **13**, Dr R Hughes; Musgrove Park Hospital, Taunton, **17**, Dr J Graham†; Norfolk and Norwich University Hospital, **58**, Dr R Wade; North Wales Cancer Treatment Centre, **1**, Dr Al-Samarraie; Dr M Latif, Northampton General Hospital, **54**, Dr C Elwell; Pinderfields Hospital, Wakefield, **6**, Dr C Coyle; Poole General Hospital, **6**, Dr J Davies; Princess Alexandra Hospital, Harlow, **5**, Dr N Gupta; Queen Elizabeth Hospital, Birmingham, **69**, Dr D Ford, Dr A Zarkar; Queen's Hospital, Romford, **11**, Dr S Gibbs; Royal Berkshire Hospital, **16**, Dr R Brown; Royal Blackburn Hospital, **11**, Dr O Parikh; Royal Bolton Hospital, **5**, Dr P Elliot; Royal Free Hospital, London, **8**, Dr M Vilarino-Varela; Royal Marsden Hospital, London, **159**, Dr V Khoo†; Royal Marsden Hospital, Sutton, **371**, Professor D Dearnaley†; Royal Oldham Hospital **2**, Dr J Livsey; Royal Preston Hospital, **107**, Dr A Birtle; Royal Surrey County Hospital, **100**, Dr J Money-Kyrle; Royal Sussex County Hospital, **57**, Dr D Bloomfield; Royal United Hospital, Bath, **11**, Dr H Newman, Dr M Beresford; Southport General Infirmary, **64**, Dr C Eswar, Dr A Siva; St Bartholomew's Hospital, London, **35**, Dr P Wells; St James's University Hospital, Leeds, **28**, Dr C Coyle, Dr A Henry; St. Luke's Hospital, Dublin, **10**, Dr P Thirion; St. Mary's Hospital, Paddington, London, **7**, Dr S Mangar; Torbay District General Hospital, **5**, Dr A Lydon; UniversitatsSpital Zurich, **7**, Dr D Zwahlen; University College Hospital, Galway, **10**, Dr J Martin; University Hospital Coventry, **70**, Dr A Stockdale; University Hospital of North Staffordshire, **32**, Dr F Adab; Velindre Hospital, Cardiff, **86**, Dr J Staffurth†; Warrington Hospital, **108**, Dr I Syndikus; West Suffolk Hospital, **59**, Dr C Woodward; Western General Hospital, Edinburgh, **2**, Dr D McLaren; Weston Park Hospital, Sheffield, **81**, Dr P Kirkbride, Dr C Ferguson; Whipps Cross University Hospital, London, **23**, Dr P Wells; Whiston Hospital, Merseyside, **71**, Dr Malik; Worcester Royal Infirmary, **19**, Dr A Stockdale; Worthing Hospital, **59**, Dr D Bloomfield,Dr A Nikapota.

Table 1: Summary of gross tumour volume (GTV), clinical target volume (CTV) and planning target volume (PTV) definitions and dose levels in each treatment group

Low risk of SV involvement[(1)]		Moderate risk of SV involvement[(1)]		Dose				Minimum iso-dose coverage
				74Gy Group	60Gy Group	57Gy Group	2Gy equivalent[+++]	
GTV1<br>CTV1<br>PTV1	P<br>P+base of SV+5mm<br>CTV1 +5mm	GTV1<br>CTV1<br>PTV1	P<br>P+SV+5mm<br>CTV1 +5mm	59.2	48.0	45.6	54Gy	76%
GTV2<br>CTV2<br>PTV2	P<br>P+5mm<br>CTV2 +5mm/0mm*	GTV2<br>CTV2<br>PTV2	P<br>P± base of SV[++] +5mm<br>CTV2 + 5mm/0mm*	71.0	57.6	54.7	70Gy	91%
GTV3<br>CTV3<br>PTV3	P<br>P+0mm<br>CTV3 +5mm/0mm[+]	GTV3<br>CTV3<br>PTV3	P<br>P+0mm<br>CTV3 + 5mm/0mm[+]	74.0	60.0	57.0	74Gy	95%

* 0mm posteriorly toward rectum unless moderate to large rectum then 5mm posteriorly towards rectum to be individualised for each CT image

[+] 0mm posteriorly towards rectum all patients

[++] Include base if T3B on MRI

[+++] Calculated for $\alpha/\beta = 3.0$ for 74Gy Group

P=Prostate; SV=seminal vesicles

1. Diaz A, Roach M 3rd, Marquez C, et al. Indications for and the significance of seminal vesicle irradiation during 3D conformal radiotherapy for localized prostate cancer. *International journal of radiation oncology, biology, physics* 1994; **30**(1): 323–329.

Table 2: Normal Tissue Dose Constraints

	Dose for 2Gy/f<br>Prescribed Dose	Dose<br>(%)	Max Vol<br>(% or cc)
Rectum	30*<br>40*<br>50<br>60<br>65<br>70<br>74	41<br>54<br>68<br>81<br>88<br>95<br>100	80%<br>70%<br>60%<br>50%<br>30%<br>15%<br>3%
Bladder	50<br>60<br>74	68<br>81<br>100	50%<br>25%<br>5%
Femoral Heads	50	68	50%
Bowel	50	68	17cc
Urethral Bulb	50*<br>60*	68<br>81	50%<br>10%

*Dose constraints optimal / non-mandatory

Table 3: Recruitment by centre

Centre	Patients
Royal Marsden Hospital, Sutton	371
Clatterbridge Centre for Oncology	177
Royal Marsden Hospital, London	159
Lincoln County Hospital	155
Ipswich Hospital	153
Warrington Hospital	108
Royal Preston Hospital	107
Christie Hospital, Manchester	102
Addenbrooke's Hospital	100
Royal Surrey County Hospital	100
Belfast City Hospital	92
Velindre Hospital, Cardiff	86
Weston Park Hospital, Sheffield	81
Whiston Hospital, Merseyside	71
University Hospital Coventry, Coventry	70
Queen Elizabeth Hospital, Birmingham	69
Southport General Infirmary	64
Beatson Oncology Centre	62
Bedford Hospital, North Wing	61
West Suffolk Hospital	59
Worthing Hospital	59
Norfolk and Norwich University Hospital	58
Kings Lynn and Wisbech NHS Hospital	57
Royal Sussex County Hospital	57
Northampton General Hospital	54
Ayr Hospital	46
Hammersmith Hospital	37
St Bartholomew's Hospital, London	35
Countess of Chester Hospital	33
University Hospital of North Staffordshire	32
Eastbourne District General Hospital	29
Freeman Hospital, Newcastle	29
Mayday University Hospital	28
St James's University Hospital, Leeds	28
Bristol Haematology and Oncology Centre	26
Heartlands Hospital, Birmingham	24
Alexandra Hospital, Redditch	23
Whipps Cross University Hospital, London	23
Basingstoke and North Hampshire Hospital	22
Hereford County Hospital	19
Worcester Royal Infirmary	19
Musgrove Park Hospital, Taunton	17

**Centre**	**Patients**
Royal Berkshire Hospital	16
Bradford Royal Infirmary	14
Mount Vernon Hospital	13
Queen's Hospital, Romford	11
Royal Blackburn Hospital	11
Royal United Hospital, Bath	11
Auckland Hospital, New Zealand	10
St. Luke's Hospital, Dublin	10
University College Hospital, Galway	10
Charing Cross Hospital	9
Halton Hospital, Runcorn	9
Clayton Hospital, Wakefield	8
James Paget Hospital, Great Yarmouth	8
Royal Free Hospital, London	8
St. Mary's Hospital, Paddington, London	7
UniversitatsSpital, Zurich	7
Maidstone District General Hospital	6
Pinderfields Hospital, Wakefield	6
Poole General Hospital	6
Burnley General Hospital	5
Cheltenham General Hospital	5
Princess Alexandra Hospital, Harlow	5
Royal Bolton Hospital	5
Torbay District General Hospital	5
Good Hope Hospital	3
Royal Oldham Hospital	2
Western General Hospital, Edinburgh	2
Dorset County Hospital	1
North Wales Cancer Treatment Centre	1

Table 4: Outcome measures by fractionation schedule

Endpoint	Number of events	Estimated proportion event-free by 2 years	Estimated proportion event-free by 5 years	Comparison to control			Comparison of hypofractionated schedules		
	n/patients; %	%; 95% CI	%; 95% CI	HR	95% CI	p-value*	HR	95% CI	p-value**
Biochemical/ clinical failure									
74Gy	136/1065 (12.8%)	97.3 (96.1, 98.1)	88.3 (86.0, 90.2)	1.00	—	—			
60Gy	118/1074 (11.0%)	98.2 (97.2, 98.8)	90.6 (88.5, 92.3)	0.84	0.65, 1.07	0.16	0.70	0.55, 0.88	0.0026
57Gy	163/1077 (15.1%)	96.7 (95.4, 97.6)	85.9 (83.4, 88.0)	1.20	0.96, 1.51	0.11	1.00	—	—
Overall survival									
74Gy	92/1065 (8.6%)	98.2 (97.2, 98.8)	92.8 (90.9, 94.3)	1.00	—	—			
60Gy	73/1074 (6.8%)	98.7 (97.8, 99.2)	94.7 (93.0, 96.0)	0.78	0.57, 1.05	0.10	0.85	0.62, 1.15	0.29
57Gy	87/1077 (8.1%)	98.5 (97.6, 99.1)	93.9 (92.1, 95.2)	0.92	0.68, 1.23	0.58	1.00	—	—
Recommencement of ADT									
74Gy	80/1065 (7.5%)	98.4 (97.4, 99.0)	93.5 (91.7, 94.9)	1.00	—	—			
60Gy	70/1074 (6.5%)	98.9 (98.1, 99.4)	95.3 (93.7, 96.5)	0.85	0.62, 1.17	0.32	0.79	0.57, 1.07	0.13
57Gy	89/1077 (8.3%)	98.7 (97.8, 99.2)	92.3 (90.4, 93.9)	1.08	0.80, 1.46	0.63	1.00	—	—
Development of metastases									
74Gy	32/1065 (3.0%)	99.4 (98.7, 99.7)	97.5 (96.3, 98.4)	1.00	—	—			
60Gy	29/1074 (2.7%)	99.4 (98.7, 99.7)	97.9 (96.7, 98.6)	0.89	0.54, 1.46	0.64	0.69	0.43, 1.11	0.12
57Gy	42/1077 (3.9%)	99.2 (98.5, 99.6)	97.4 (96.1, 98.2)	1.28	0.81, 2.03	0.29	1.00	—	—
Disease free survival									
74Gy	209/1065 (19.6%)	95.5 (94.1, 96.6)	82.3 (79.6, 84.6)	1.00	—	—			
60Gy	179/1074 (16.7%)	96.9 (95.6, 97.8)	85.3 (82.8, 87.5)	0.83	0.68, 1.01	0.065	0.76	0.63, 0.93	0.0067
57Gy	227/1077 (21.1%)	95.4 (93.9, 96.5)	80.1 (77.3, 82.6)	1.08	0.90, 1.31	0.40	1.00	—	—

ADT=Androgen deprivation therapy. *Assessed with log-rank test comparing each hypofractionated schedule to 74Gy. **Assessed with log-rank test comparing 60Gy to 57Gy.

Table 5: Multivariable analyses of biochemical/clinical failure adjusting for clinically prognostic factors

		Events/N	HR	95% CI	p-value
Treatment group	74Gy/37f	134/1053	1.00	—	—
	60Gy/20f	118/1068	0.86	0.67, 1.11*	0.25
	57Gy/19f	162/1066	1.21	0.96, 1.52**	0.11
Age	≤69	222/1589	1.00	—	—
	>69	192/1598	0.84	0.69, 1.03	0.087
Risk group	Low	34/481	1.00	—	—
	Intermediate	319/2327	1.06	0.69, 1.64	0.80
	High	61/379	1.24	0.72, 2.14	0.43
Gleason score	≤6	98/1114	1.00	—	—
	≥7	316/2073	1.92	1.46, 2.53	p<0.0001
T stage	T1	105/1165	1.00	—	—
	T2	256/1749	1.63	1.29, 2.06	p<0.0001
	T3	53/273	1.81	1.21, 2.70	0.0038
Pre-ADT PSA (ng/ml)	<10	153/1565	1.00	—	—
	≤10, >20	215/1414	1.57	1.27, 1.96	p<0.0001
	≥20	46/208	2.26	1.57, 3.25	p<0.0001

* 90% CI: 0.70, 1.06 ** 90% CI: 0.99, 1.46

ADT=Androgen deprivation therapy

Table 6a: Physician (grade 2 or worse) and patient reported (small or worse) late bowel side effects by fractionation schedule

	Cumulative number of events	Estimated proportion with event by 2 years	Estimated proportion with event by 5 years	Hazard ratio (95% CI), p-value*		Proportion with event at 2 years†			Proportion with event at 5 years		
	n/patients; %	%; 95% CI	%; 95% CI	Comparison to control	Comparison of hRT schedules	n/patients; %	Comparison to control	Comparison of hRT schedules	n/patients; %	Comparison to control	Comparison of hRT schedules
RTOG											
74Gy	111/1040 (10.7%)	8.0 (6.5, 9.9)	13.7 (10.8, 17.4)	1.00		35/922 (3.8%)			7/534 (1.3%)		
60Gy	105/1049 (10.0%)	8.6 (7.1, 10.5)	11.9 (9.6, 14.8)	0.94 (0.72, 1.23), p=0.65	1.12 (0.84, 1.47), p=0.44	28/959 (2.9%)	p=0.31	p=0.10	13/569 (2.3%)	p=0.26	p=0.84
57Gy	95/1057 (9.0%)	6.9 (5.5, 8.6)	11.3 (8.9, 14.3)	0.84 (0.64, 1.11), p=0.22	1.00	17/962 (1.8%)	**p=0.0075**		11/549 (2.0%)	p=0.48	
RMH											
74Gy	133/1040 (12.8%)	10.4 (8.7, 12.4)	15.9 (12.7, 19.8)	1.00		49/919 (5.3%)			12/524 (2.3%)		
60Gy	136/1049 (13.0%)	10.5 (8.7, 12.5)	15.3 (12.5, 18.8)	1.02 (0.80, 1.29), p=0.89	1.01 (0.80, 1.29), p=0.91	36/953 (3.8%)	p=0.12	p=0.11	18/563 (3.2%)	p=0.46	p=0.60
57Gy	135/1057 (12.8%)	9.6 (8.0, 11.6)	15.5 (12.7, 18.9)	1.00 (0.79, 1.27), p=0.98	1.00	23/953 (2.4%)	**p=0.0011**		14/536 (2.6%)	p=0.84	
LENT/SOM											
74Gy	210/1040 (20.2%)	16.0 (13.9, 18.4)	24.3 (20.2, 29.1)	1.00		69/893 (7.7%)			22/520 (4.2%)		
60Gy	228/1049 (21.7%)	17.6 (15.4, 20.1)	25.8 (22.6, 29.5)	1.09 (0.90, 1.31), p=0.37	1.39 (1.14, 1.70), **p=0.0010**	50/930 (5.4%)	p=0.046	p=0.39	29/555 (5.2%)	p=0.48	p=0.32
57Gy	170/1057 (16.1%)	12.9 (11.0, 15.1)	18.4 (15.6, 21.7)	0.78 (0.64, 0.96), p=0.016	1.00	41/930 (4.4%)	**p=0.0031**		21/534 (3.9%)	p=0.88	
EPIC/UCLA											
74Gy	202/677 (29.8%)	24.6 (21.4, 28.2)	45.2 (36.6, 54.8)	1.00		53/431 (12.3%)			49/341 (14.4%)		
60Gy	225/682 (33.0%)	27.1 (23.8, 30.)	44.7 (37.4, 52.6)	1.12 (0.92, 1.35), p=0.25	1.19 (0.98, 1.44), p=0.073	58/426 (13.6%)	p=0.57	p=0.77	57/375 (15.2%)	p=0.76	p=0.99
57Gy	202/693 (29.1%)	24.8 (21.7, 28.4)	41.2 (34.7, 48.4)	0.94 (0.77, 1.14), p=0.52	1.00	65/455 (14.3%)	0.38		59/387 (15.2%)	p=0.74	

*Assessed with a log-rank test. †Comparison assessed with a Fisher's exact test. Proportion with event at two years includes men with an assessment within 3 months of the expected 2 year visit. Proportion with event at five years includes men with an assessment within 6 months of the expected 5 year visit.

hRT=hypofractionated radiotherapy

Table 6b: Physician (grade 2 or worse) and patient reported (small or worse) late bladder side effects by fractionation schedule

	Cumulative number of events	Estimated proportion with event by 2 years	Estimated proportion with event by 5 years	Hazard ratio (95% CI), p-value*		Proportion with event at 2 years[†]			Proportion with event at 5 years		
	n/patients; %	%; 95% CI	%; 95% CI	Comparison to control	Comparison of hRT schedules	n/patients; %	Comparison to control	Comparison of hRT schedules	n/patients; %	Comparison to control	Comparison of hRT schedules
RTOG											
74Gy	66/1040 (6.4%)	3.9 (2.9, 5.3)	9.1 (6.5, 12.8)	1.00		13/922 (1.4%)			9/534 (1.7%)		
60Gy	88/1049 (8.4%)	5.7 (4.5, 7.3)	11.7 (8.4, 16.1)	1.34 (0.98, 1.85), p=0.070	1.58 (1.13, 2.20), **p=0.0073**	16/959 (1.7%)	p=0.71	p=0.34	10/569 (1.8%)	p=1.00	p=1.00
57Gy	57/1057 (5.4%)	4.1 (3.0, 5.4)	6.6 (4.9, 8.7)	0.85 (0.60, 1.21), p=0.37	1.00	11/962 (1.1%)	p=0.68		10/549 (1.8%)	p=1.00	
RMH											
74Gy	260/1040 (25.0%)	18.8 (16.5, 21.3)	31.0 (26.3, 36.3)	1.00		83/918 (9.0%)			41/522 (7.9%)		
60Gy	286/1049 (27.3%)	21.3 (18.9, 23.9)	34.1 (28.8, 40.0)	1.12 (0.94, 1.32), p=0.20	1.13 (0.95, 1.33), p=0.16	92/955 (9.6%)	p=0.69	**p=0.0036**	47/563 (8.3%)	p=0.82	p=1.00
57Gy	260/1057 (24.6%)	18.1 (15.9, 20.75	28.7 (25.5, 32.3)	0.99 (0.83, 1.18), p=0.91	1.00	57/954 (6.0%)	p=0.014		45/534 (8.4%)	p=0.74	
LENT/SOM											
74Gy	390/1040 (37.5%)	28.8 (26.2, 31.7)	49.1 (42.9, 55.8)	1.00		114/891 (12.8%)			70/518 (13.5%)		
60Gy	409/1049 (39.0%)	30.9 (28.2, 33.8)	49.9 (43.8, 56.4)	1.06 (0.93, 1.22), p=0.39	1.19 (1.03, 1.37), p=0.015	127/928 (13.7%)	p=0.58	p=0.065	73/555 (13.2%)	p=0.93	p=0.35
57Gy	359/1057 (34.0%)	26.3 (23.7, 29.1)	41.4 (36.9, 46.1)	0.89 (0.77, 1.03), p=0.12	1.00	100/926 (10.8%)	p=0.19		59/526 (11.2%)	p=0.30	
EPIC/UCLA											
74Gy	202/677 (29.8%)	23.9 (20.7, 27.5)	42.5 (34.6, 51.3)	1.00		50/425 (11.8%)			56/333 (16.8%)		
60Gy	200/682 (29.3%)	23.2 (20.1, 26.7)	43.2 (35.6, 51.6)	0.95 (0.78, 1.16), p=0.60	1.03 (0.85, 1.26), p=0.74	60/425 (14.1%)	p=0.31	p=0.93	63/371 (17.0%)	p=0.95	p=0.71
57Gy	196/693 (28.3%)	24.0 (20.8, 27.5)	37.4 (32.4, 42.9)	0.92 (0.76, 1.12), p=0.41	1.00	63/453 (13.9%)	p=0.34		60/376 (16.0%)	p=0.76	

*Assessed with a log-rank test. [†]Comparison assessed with a Fisher's exact test. Proportion with event at two years includes men with an assessment within 3 months of the expected 2 year visit. Proportion with event at five years includes men with an assessment within 6 months of the expected 5 year visit.

hRT=hypofractionated radiotherapy

Table 6c: Physician (grade 2 or worse) and patient reported (small or worse) late sexual side effects by fractionation schedule

	Cumulative number of events	Estimated proportion with event by 2 years	Estimated proportion with event by 5 years	Hazard ratio (95% CI), p-value*		Proportion with event at 2 years†			Proportion with event at 5 years		
	n/patients; %	%; 95% CI	%; 95% CI	Comparison to control	Comparison of hRT schedules	n/patients; %	Comparison to control	Comparison of hRT schedules	n/patients; %	Comparison to control	Comparison of hRT schedules
LENT/SOM											
74Gy	899/1040 (86.4%)	82.4 (80.0, 84.7)	93.3 (87.8, 96.9)	1.00		550/826 (66.6%)			305/454 (67.2%)		
60Gy	892/1049 (85.0%)	80.1 (77.6, 82.5)	89.3 (86.1, 92.1)	0.95 (0.87, 1.04), p=0.30	0.95 (0.87, 1.05), p=0.32	562/864 (65.0%)	p=0.54	p=0.76	311/499 (62.3%)	p=0.12	p=0.042
57Gy	914/1057 (86.5%)	81.4 (79.0, 83.8)	89.8 (87.3, 92.0)	1.00 (0.91, 1.09), p=0.96	1.00	552/859 (64.3%)	p=0.33		318/463 (68.7%)	p=0.67	
EPIC/UCLA											
74Gy	473/677 (69.9%)	67.4 (63.6, 71.1)	88.1 (80.8, 93.6)	1.00		207/413 (50.1%)			187/357 (51.5%)		
60Gy	489/682 (71.7%)	67.4 (63.7, 71.0)	84.1 (77.6, 89.5)	1.04 (0.91, 1.18), p=0.58	1.01 (0.89, 1.15), p=0.87	224/410 (54.6%)	p=0.20	0.97	184/357 (51.5%)	p=0.12	p=0.75
57Gy	490/693 (70.7%)	68.2 (64.5, 71.8)	83.4 (77.7, 88.3)	1.03 (0.91, 1.17), p=0.67	1.00	236/433 (54.5%)	p=0.20		195/370 (52.7%)	p=0.20	

*Assessed with a log-rank test. †Comparison assessed with a Fisher's exact test. Proportion with event at two years includes men with an assessment within 3 months of the expected 2 year visit. Proportion with event at five years includes men with an assessment within 6 months of the expected 5 year visit.

hRT=hypofractionated radiotherapy

Table 7a: Physician (grade 1 or worse) and patient reported (very small or worse) late bowel side effects by fractionation schedule

	Cumulative number of events	Estimated proportion with event by 2y	Estimated proportion with event by 5y	Hazard ratio (95% CI), p-value*		Proportion with event at 2y†			Proportion with event at 5y		
	n/patients; %	%; 95% CI	%; 95% CI	Comparison to control	Comparison of hRT schedules	n/patients; %	Comparison to control	Comparison of hRT schedules	n/patients; %	Comparison to control	Comparison of hRT schedules
RTOG											
74Gy	342/1040 (32.9%)	27.5 (24.8, 30.3)	36.8 (33.3, 40.6)	1.00		115/922 (12.5%)			35/534 (6.6%)		
60Gy	352/1049 (33.6%)	27.9 (25.3, 30.8)	41.9 (36.0, 48.4)	1.02 (0.88, 1.19), p=0.76	1.16 (0.997, 1.35), p=0.055	96/959 (10.0%)	p=0.093	p=0.24	38/569 (6.7%)	p=1.00	p=0.90
57Gy	313/1057 (29.6%)	24.2 (21.7, 26.9)	34.2 (30.3, 38.5)	0.88 (0.76, 1.03), p=0.11	1.00	81/962 (8.4%)	**p=0.0041**		35/549 (6.4%)	p=1.00	
RMH											
74Gy	497/1040 (47.8%)	40.0 (37.0, 43.0)	52.8 (49.2, 56.6)	1.00		211/919 (23.0%)			89/524 (17.0%)		
60Gy	520/1049 (49.6%)	41.8 (38.8, 44.8)	58.2 (53.3, 63.3)	1.06 (0.94, 1.20), p=0.32	1.13 (0.998, 1.28), p=0.053	203/953 (21.3%)	p=0.40	p=0.53	97/563 (17.2%)	p=0.94	p=0.52
57Gy	478/1057 (45.2%)	37.6 (34.7, 40.6)	51.0 (47.2, 55.0)	0.94 (0.83, 1.07), p=0.34	1.00	191/953 (20.0%)	p=0.13		84/536 (15.7%)	p=0.62	
LENT/SOM											
74Gy	513/1040 (49.3%)	41.7 (38.8, 44.8)	58.2 (52.6, 63.8)	1.00		216/893 (24.2%)			83/520 (16.0%)		
60Gy	548/1049 (52.2%)	44.7 (41.7, 47.8)	59.9 (55.0, 64.8)	1.10 (0.97, 1.24), p=0.12	1.21 (1.07, 1.37), **p=0.0023**	198/930 (21.3%)	p=0.15	p=0.42	91/555 (16.4%)	p=0.87	p=0.50
57Gy	490/1057 (46.4%)	38.8 (35.9, 41.8)	52.2 (47.8, 56.7)	0.91 (0.80, 1.03), p=0.12	1.00	183/930 (19.7%)	p=0.020		79/534 (14.8%)	p=0.61	
EPIC/UCLA											
74Gy	389/977 (57.5%)	52.6 (48.8, 56.6)	73.1 (66.5, 79.4)	1.00		150/431 (34.8%)			135/341		
60Gy	395/682 (57.9%)	53.0 (49.1, 56.9)	74.2 (65.2, 82.3)	1.02 (0.89, 1.17), p=0.77	1.03 (0.90, 1.18), p=0.69	153/426	p=0.73	p=0.82	141/375	p=0.59	p=0.35
57Gy	405/693 (58.4%)	52.0 (48.2, 55.9)	71.8 (65.0, 78.4)	0.99 (0.86, 1.14), p=0.86	1.00	160/455	p=0.91		133/387	p=0.15	

*Assessed with a log-rank test. †Comparison assessed with a Fisher's exact test. Proportion with event at two years includes men with an assessment within 3 months of the expected 2 year visit. Proportion with event at five years includes men with an assessment within 6 months of the expected 5 year visit.

hRT=hypofractionated radiotherapy

Table 7b: Physician (grade 1 or worse) and patient reported (very small or worse) late bladder side effects by fractionation schedule

	Cumulative number of events	Estimated proportion with event by 2y	Estimated proportion with event by 5y	Hazard ratio (95% CI), p-value*		Proportion with event at 2y†			Proportion with event at 5y		
	n/patients; %	%; 95% CI	%; 95% CI	Comparison to control	Comparison of hRT schedules	n/patients; %	Comparison to control	Comparison of hRT schedules	n/patients; %	Comparison to control	Comparison of hRT schedules
RTOG											
74Gy	182/1040 (17.5%)	12.6 (10.7, 14.7)	20.5 (17.5, 23.9)	1.00		43/922 (4.7%)			17/534 (3.2%)		
60Gy	201/1049 (19.2%)	13.7 (11.8, 16.0)	28.2 (22.2, 35.4)	1.11 (0.91, 1.36), p=0.30	1.23 (1.00, 1.51), p=0.049	40/959 (4.2%)	p=0.65	p=0.66	25/569 (3.2%)	p=0.35	p=0.88
57Gy	169/1057 (16.0%)	12.3 (10.4, 14.4)	19.6 (16.4, 23.3)	0.91 (0.74, 1.12), p=0.36	1.00	45/962 (4.7%)	p=1.00		23/549 (4.2%)	p=0.42	
RMH											
74Gy	773/1040 (74.3%)	65.2 (62.3, 68.1)	83.8 (79.1, 87.9)	1.00		366/918 (39.9%)			211/522 (40.4%)		
60Gy	758/1049 (72.3%)	64.8 (61.9, 67.7)	82.1 (76.7, 86.9)	0.98 (0.89, 1.09), p=0.74	0.997 (0.90, 1.10), p=0.95	386/955 (40.4%)	p=0.81	p=0.08	232/563 (41.2%)	p=0.81	p=0.81
57Gy	763/1057 (72.2%)	64.4 (61.5, 67.3)	80.5 (76.5, 84.1)	0.99 (0.89, 1.09), p=0.78	1.00	348/954 (36.5%)	p=0.14		224/534 (41.9%)	p=0.62	
LENT/SOM											
74Gy	671/1040 (64.5%)	53.7 (50.6, 56.7)	75.0 (70.1, 79.7)	1.00		242/891 (27.2%)			150/518 (29.0%)		
60Gy	668/1049 (63.7%)	53.2 (50.2, 56.2)	76.0 (69.5, 82.0)	0.99 (0.89, 1.10), p=0.82	1.06 (0.95, 1.18), p=0.29	262/928 (28.2%)	p=0.64	p=0.84	161/555 (29.0%)	p=1.00	p=0.95
57Gy	641/1057 (60.6%)	51.7 (48.7, 54.8)	68.4 (64.2, 72.6)	0.93 (0.84, 1.04), p=0.21	1.00	257/926 (27.8%)	p=0.79		151/526 (28.7%)	p=0.95	
EPIC/UCLA											
74Gy	404/977 (59.7%)	54.6 (50.8, 58.6)	76.8 (68.7, 84.1)	1.00		151/425			147/333		
60Gy	407/682 (59.7%)	52.4 (48.5, 56.3)	78.7 (69.9, 86.4)	0.98 (0.86, 1.13), p=0.78	1.07 (0.93, 1.23), p=0.34	170/425	p=0.18	p=0.13	150/371	p=0.32	p=0.82
57Gy	399/693 (57.6%)	51.9 (48.1, 55.8)	67.9 (62.7, 73.0)	0.92 (0.80, 1.05), p=0.23	1.00	159/453	p=0.89		149/376	p=0.22	

*Assessed with a log-rank test. †Comparison assessed with a Fisher's exact test. Proportion with event at two years includes men with an assessment within 3 months of the expected 2 year visit. Proportion with event at five years includes men with an assessment within 6 months of the expected 5 year visit.

hRT=hypofractionated radiotherapy

Table 7c: Physician (grade 1 or worse) and patient reported (very small or worse) late sexual side effects by fractionation schedule

	Cumulative number of events	Estimated proportion with event by 2y	Estimated proportion with event by 5y	Hazard ratio (95% CI), p-value*		Proportion with event at 2y†			Proportion with event at 5y		
	n/patients; %	%; 95% CI	%; 95% CI	Comparison to control	Comparison of hRT schedules	n/patients; %	Comparison to control	Comparison of hRT schedules	n/patients; %	Comparison to control	Comparison of hRT schedules
LENT/SOM											
74Gy	936/1040 (90.0%)	86.9 (84.7, 88.9)	94.3 (90.0, 97.1)	1.00		598/826 (72.4%)			329/454 (72.5%)		
60Gy	930/1049 (88.7%)	84.3 (82.1, 86.5)	93.7 (90.2, 96.2)	0.95 (0.87, 1.04), p=0.31	0.96 (0.88, 1.05), p=0.36	612/864 (70.8%)	p=0.48	p=0.92	345/499 (69.1%)	p=0.29	p=0.086
57Gy	951/1057 (90.0%)	86.2 (84.0, 88.2)	92.7 (90.7, 94.5)	0.99 (0.91, 1.09), p=0.89	1.00	606/859 (70.5%)	p=0.42		344/463 (72.3%)	p=0.55	
EPIC/UCLA											
74Gy	538/677 (79.5%)	79.7 (76.4, 82.8)	87.8 (84.6, 90.5)	1.00		287/413			245/325		
60Gy	542/682 (79.5%)	79.2 (75.9, 82.3)	90.6 (84.9, 94.8)	1.02 (0.90, 1.15), p=0.74	0.99 (0.88, 1.12), p=0.93	288/410	p=0.81	p=0.78	248/357	p=0.085	p=0.31
57Gy	566/693 (81.7%)	79.2 (76.0, 82.3)	91.4 (86.8, 94.8)	1.02 (0.91, 1.15), p=0.69	1.00	308/433	p=0.60		244/370	**p=0.0066**	

*Assessed with a log-rank test. †Comparison assessed with a Fisher's exact test. Proportion with event at two years includes men with an assessment within 3 months of the expected 2 year visit. Proportion with event at five years includes men with an assessment within 6 months of the expected 5 year visit.

hRT=hypofractionated radiotherapy

Table 8: Published contemporary randomised controlled trials of modest hypofractionation

AUTHOR	No.	Total Dose (Gy)	Fractions	Dose/Fraction (Gy)	BED			Acute Reactions RTOG G2+		Late Reactions RTOG G2+		Radiotherapy Technique	ADT
					1.8Gy(a)	3.0Gy(b)	10.0Gy(c)	GI	GU	GI	GU		
Arcangeli et al 2011[2]	85	80	40	2	170	133	96	21%	40%	14%	11%	CFRT	100%
	83	62	20	3.1	169	126	81	35%	47%	17%	16%	CFRT	100%
Pollack et al 2013[3]	151	76	38	2	162	127	91	-	47.70%	22.50%	13.40%	IMRT	47%
	152	70.2	26	2.7	177	133	89	-	44.90%	18.10%	21.50%	IMRT	45%
Hoffman et al 2014[4]	101	75.6	42	1.8	153	121	89	-	-	5.10%	16.50%	IMRT and IGRT	23%
	102	72	30	2.4	170	130	89	-	-	10.00%	15.80%	IMRT and IGRT	25%
Aluwini et al 2015[5]	410	78	39	2	166	130	94	31.20%	57.80%	-	-	CFRT	67%
	410	64.6+	19+	3.4+	189	138	87	42.0%*	60.50%	-	-	CFRT	67%
Norkus et al 2013[6]	57	76	38	2	162	127	91	40%	28%	-	-	CFRT	100%
	67	63++	20++	3.15++	175	129	83	39%	23%	-	-	CFRT	100%
CHHiP	1065	74	37	2	158	123	89	25%	46%	13.70%	9.20%	IMRT +/- IGRT	97%
	1074	60	20	3	162	120	78	38%**	49%	12.00%	11.70%	IMRT +/- IGRT	97%
	1077	57	19	3	154	114	74	38%**	46%	11.20%	6.60%	IMRT +/- IGRT	97%

Hypofractionated treatment groups are highlighted in yellow.

Treatment given with five fraction/week schedules except: + hypofractionated group treated with 3 fractions/week and ++ hypofractionated group treated with 4 fraction/week

(a) Estimated from CHHiP trial results; (b) Representative of late reacting tissue; (c) Representative of acute reaction tissue.

*p=0.0015 **p<0.001

GI – gastrointestinal, GU = genitourinary, CFRT = conformal radiotherapy, IMRT = intensity modulated radiotherapy, IGRT - image guided radiotherapy, BED = biologically equivalent dose, ADT-androgen deprivation therapy.

2. Arcangeli G, Fowler J, Gomellini S, et al. Acute and late toxicity in a randomized trial of conventional versus hypofractionated three-dimensional conformal radiotherapy for prostate cancer. *International journal of radiation oncology, biology, physics* 2011; **79**(4): 1013-21.
3. Pollack A, Walker G, Horwitz EM, et al. Randomized trial of hypofractionated external-beam radiotherapy for prostate cancer. *J Clin Oncol* 2013; **31**(31): 3860-8.
4. Hoffman KE, Voong KR, Pugh TJ, et al. Risk of late toxicity in men receiving dose-escalated hypofractionated intensity modulated prostate radiation therapy: results from a randomized trial. *International journal of radiation oncology, biology, physics* 2014; **88**(5): 1074-84.
5. Aluwini S, Pos F, Schimmel E, et al. Hypofractionated versus conventionally fractionated radiotherapy for patients with prostate cancer (HYPRO): acute toxicity results from a randomised non-inferiority phase 3 trial. *The Lancet Oncology* 2015; **16**(3): 274-83.
6. Norkus D, Karklelyte A, Engels B, et al. A randomized hypofractionation dose escalation trial for high risk prostate cancer patients: interim analysis of acute toxicity and quality of life in 124 patients. *Radiat Oncol* 2013; **8**: 206.

Figure 1: Outlining of target volumes

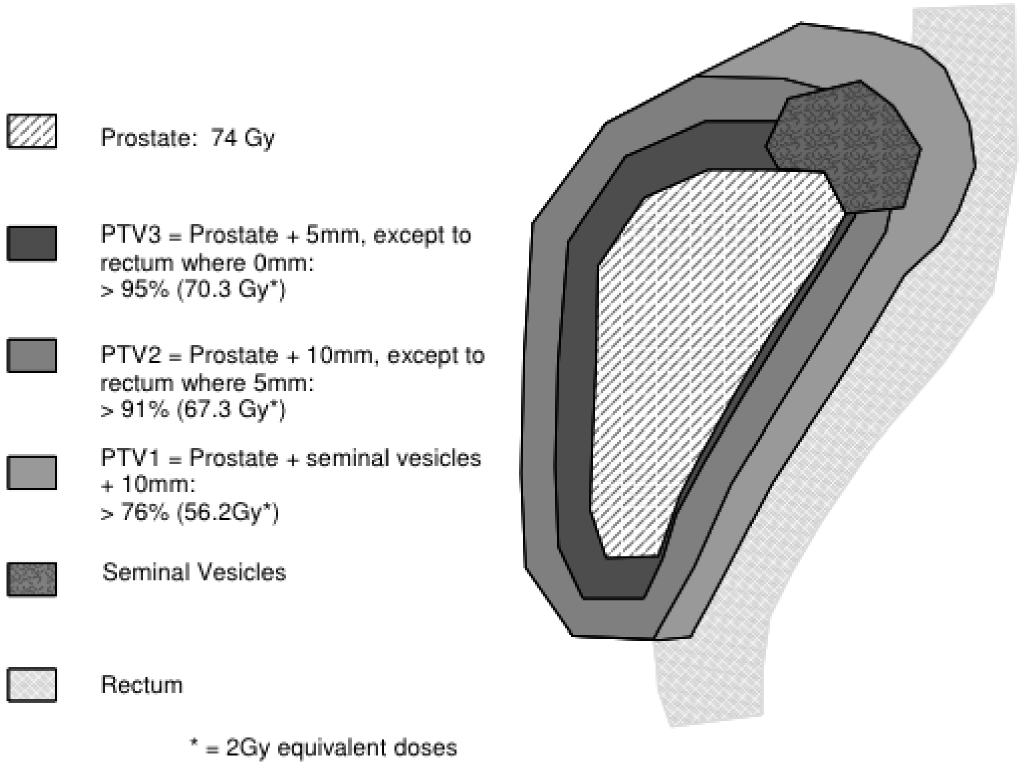

Treatment was planned and delivered using an integrated simultaneous-boost technique with target volumes designed to give the conventional 74Gy group: a dose of 59Gy (80%) to the prostate and base or all seminal vesicles, with a uniform 10mm margin; a dose of 71Gy (96%) to the prostate with a 10mm margin, except posteriorly where the margin was reduced to 5mm; and 74Gy (100%) to the prostate with a margin of 5mm (0mm posteriorly). Target volumes had to be covered by the 95% iso-dose. For the hypofractionated groups, similar proportions of the prescribed dose (i.e. 60Gy or 57Gy) were given to outer target volumes.

Figure 2: Freedom from biochemical/clinical failure in low (A), intermediate (B) and high (C) risk patients

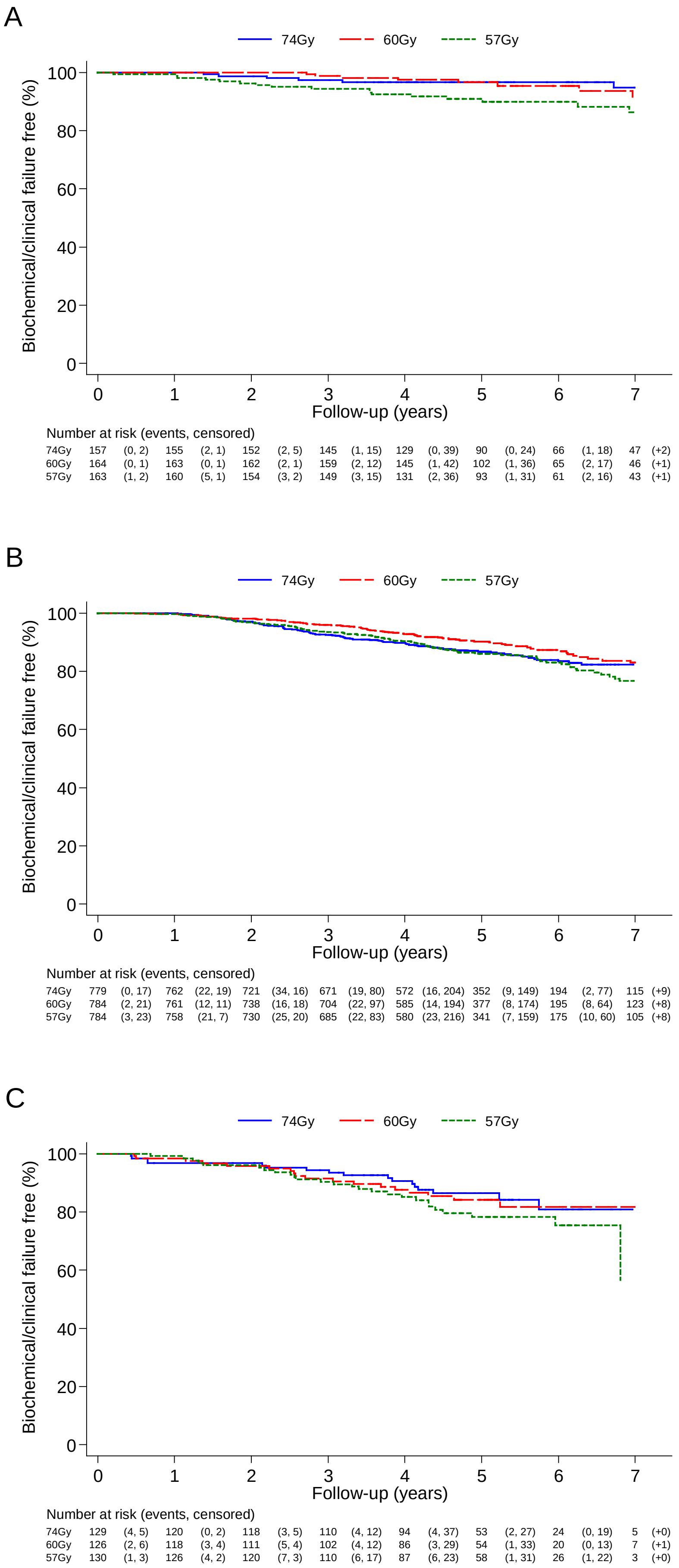

Figure 3: Recommencement of ADT (A) and development of metastases (B)

ADT=Androgen deprivation therapy

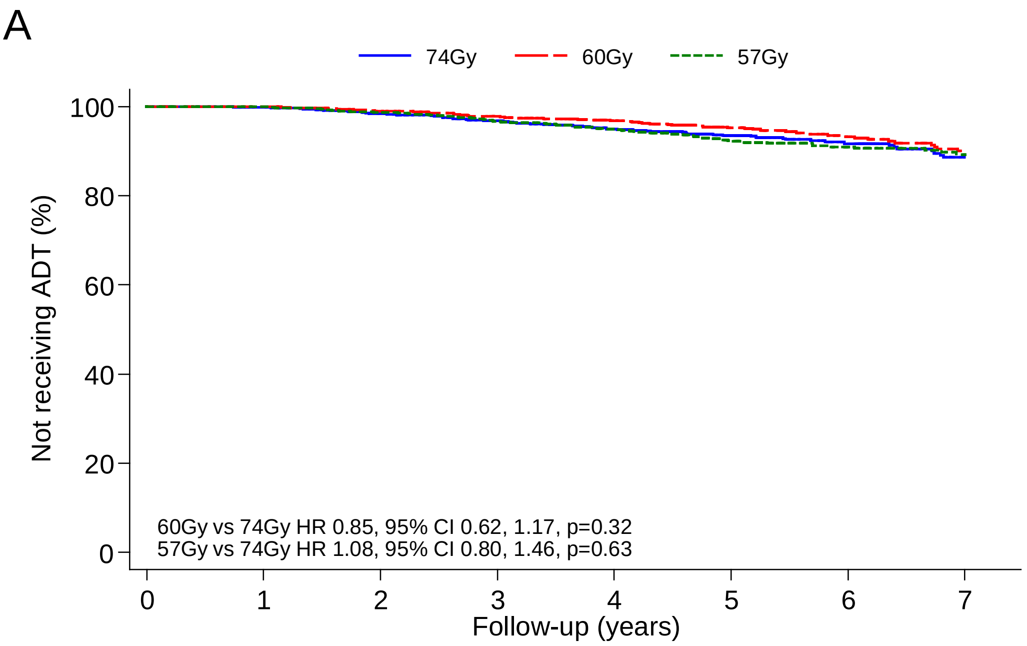

Number at risk (events, censored)

74Gy	1065	(2, 19)	1044	(15, 20)	1009	(15, 28)	966	(19, 104)	843	(10, 286)	547	(7, 230)	310	(7, 121)	182 (+5)
60Gy	1074	(1, 25)	1048	(10, 14)	1024	(12, 24)	988	(9, 113)	866	(12, 276)	578	(8, 260)	310	(8, 100)	202 (+10)
57Gy	1077	(1, 23)	1053	(13, 11)	1029	(21, 20)	988	(17, 108)	863	(19, 283)	561	(6, 244)	311	(4, 113)	194 (+8)

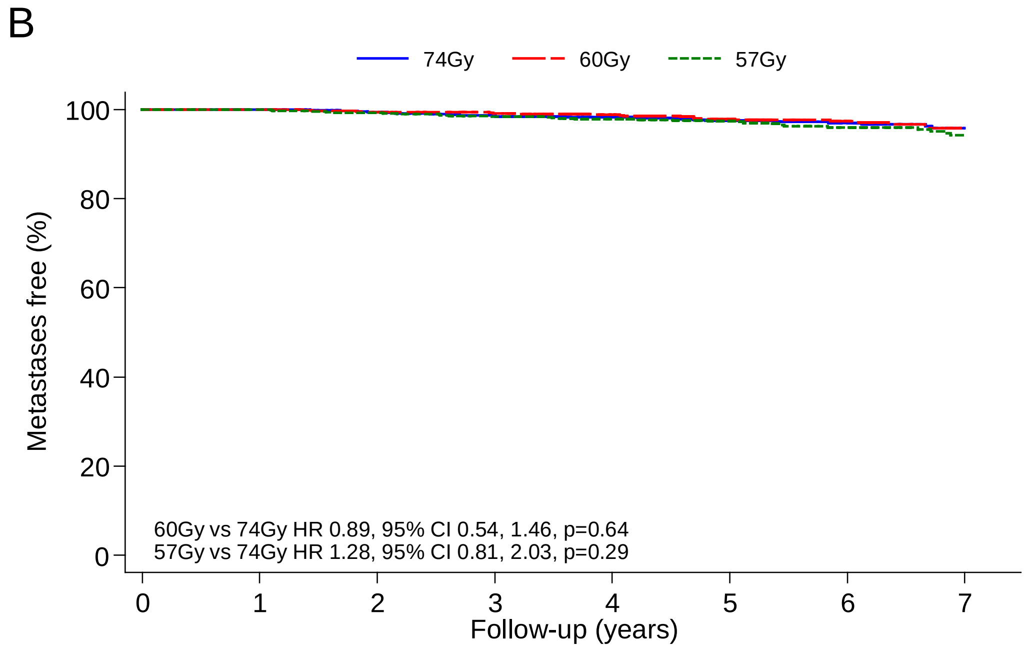

Number at risk (events, censored)

74Gy	1065	(1, 19)	1045	(5, 19)	1021	(9, 28)	984	(3, 104)	877	(5, 299)	573	(2, 242)	329	(3, 129)	197 (+4)
60Gy	1074	(1, 25)	1048	(5, 14)	1029	(3, 24)	1002	(3, 114)	885	(7, 281)	597	(2, 268)	327	(5, 107)	215 (+3)
57Gy	1077	(1, 23)	1053	(7, 11)	1035	(9, 20)	1006	(5, 108)	893	(4, 298)	591	(6, 260)	325	(4, 115)	206 (+6)

Figure 4: Acute RTOG toxicity by time-point and randomised treatment group

Distribution of bowel toxicity grades (A) and distribution of bladder toxicity grades (B)

RTOG = Radiation Therapy Oncology Group scale

G1+ = score of grade 1 or worse. G2+ = score of grade 2 or worse. G3+ = score of grade 3 or worse.

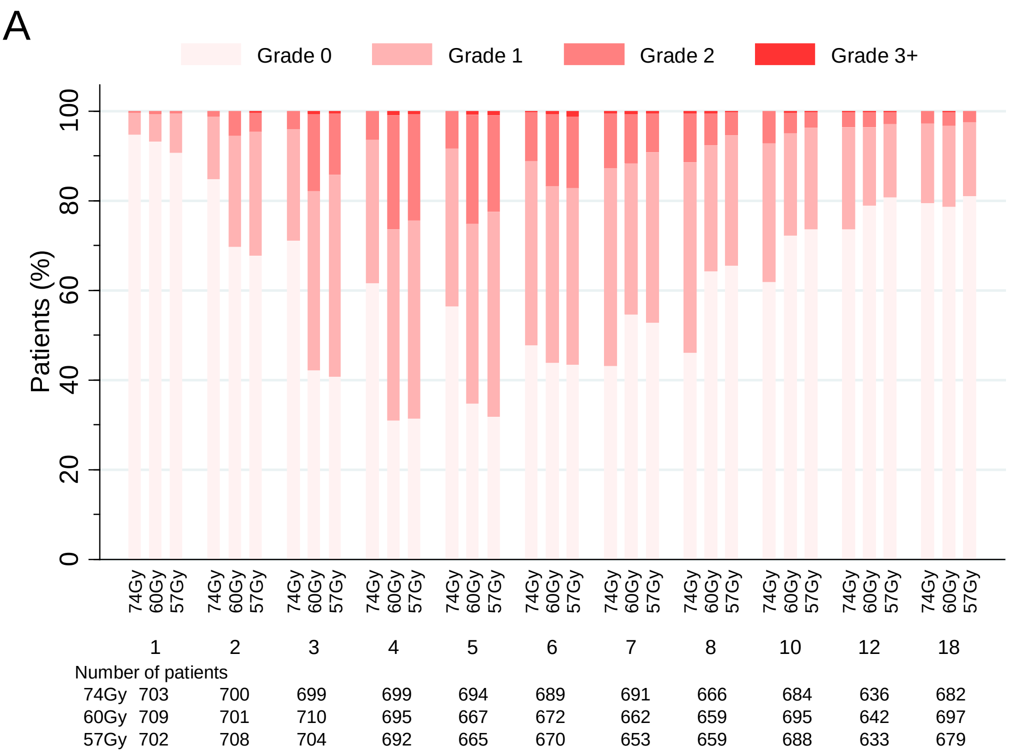

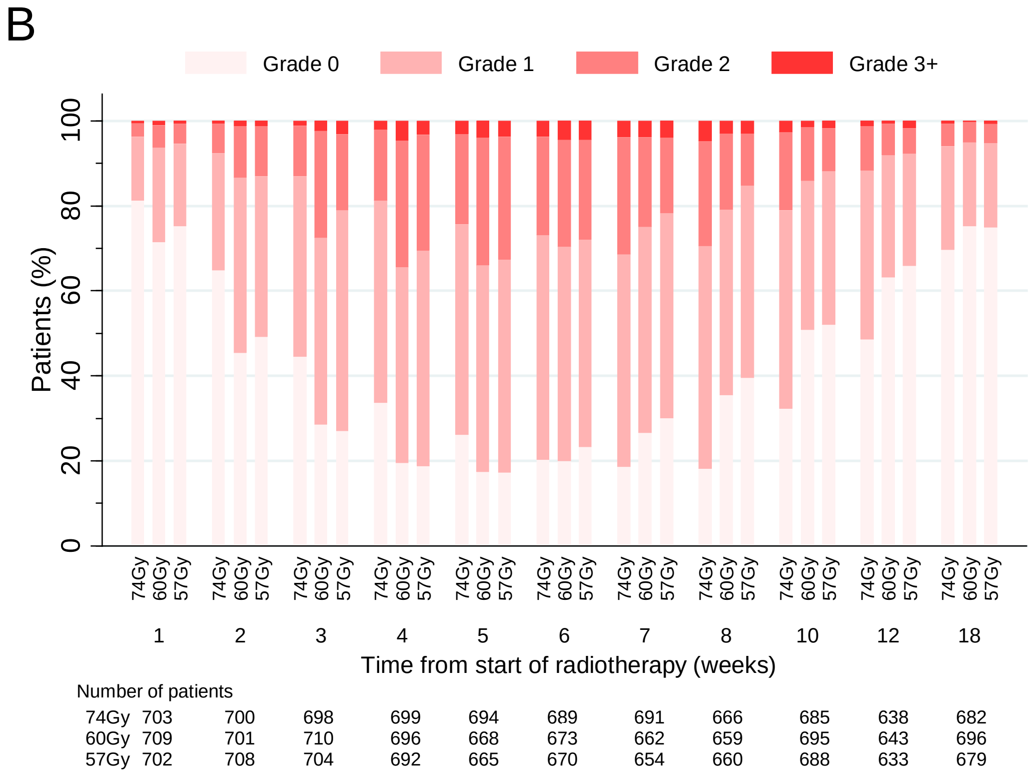

Figure 5: Late bowel toxicity by time-point, assessment and randomised treatment group

Distribution of bowel scores with RMH (A), LENT/SOM (C), UCLA PCI/EPIC (E). Cumulative proportion of bowel scores measured with RMH (B) and LENT/SOM (D).

RMH=Royal Marsden Hospital scale. LENT/SOM=Late Effects on Normal Tissue: Subjective/Objective/Management scale. UCLA PCI=UCLA Prostate cancer index; EPIC=Expanded Prostate Cancer Index Composite.

PA=before ADT. PR=before radiotherapy. ADT= androgen deprivation therapy. RT=radiotherapy. m=months

G1+ = score of grade 1 or worse. G2+ = score of grade 2 or worse. G3+ = score of grade 3 or worse.

Very small+ = score of very small, small, moderate or big bother. Small+ = score of small, moderate or big bother. Moderate+ = score of moderate or worse bother.

Late toxicity data has been included in analyses if was reported within 6 weeks of the 6 month visit, 3 months of the 12-24 month visit and within 6 months of 36-60 month visit. For LENT/SOM and UCLA/EPIC PA data is included if it was reported within 3 months prior to starting ADT and within 1 month after starting ADT. For RMH, all PA data has been used. PR data is included if it was reported within 3 months prior to RT and no more than 7 days after starting RT. Time-to-event analyses use all data reported from 6 weeks prior to the 6 month visit onwards.

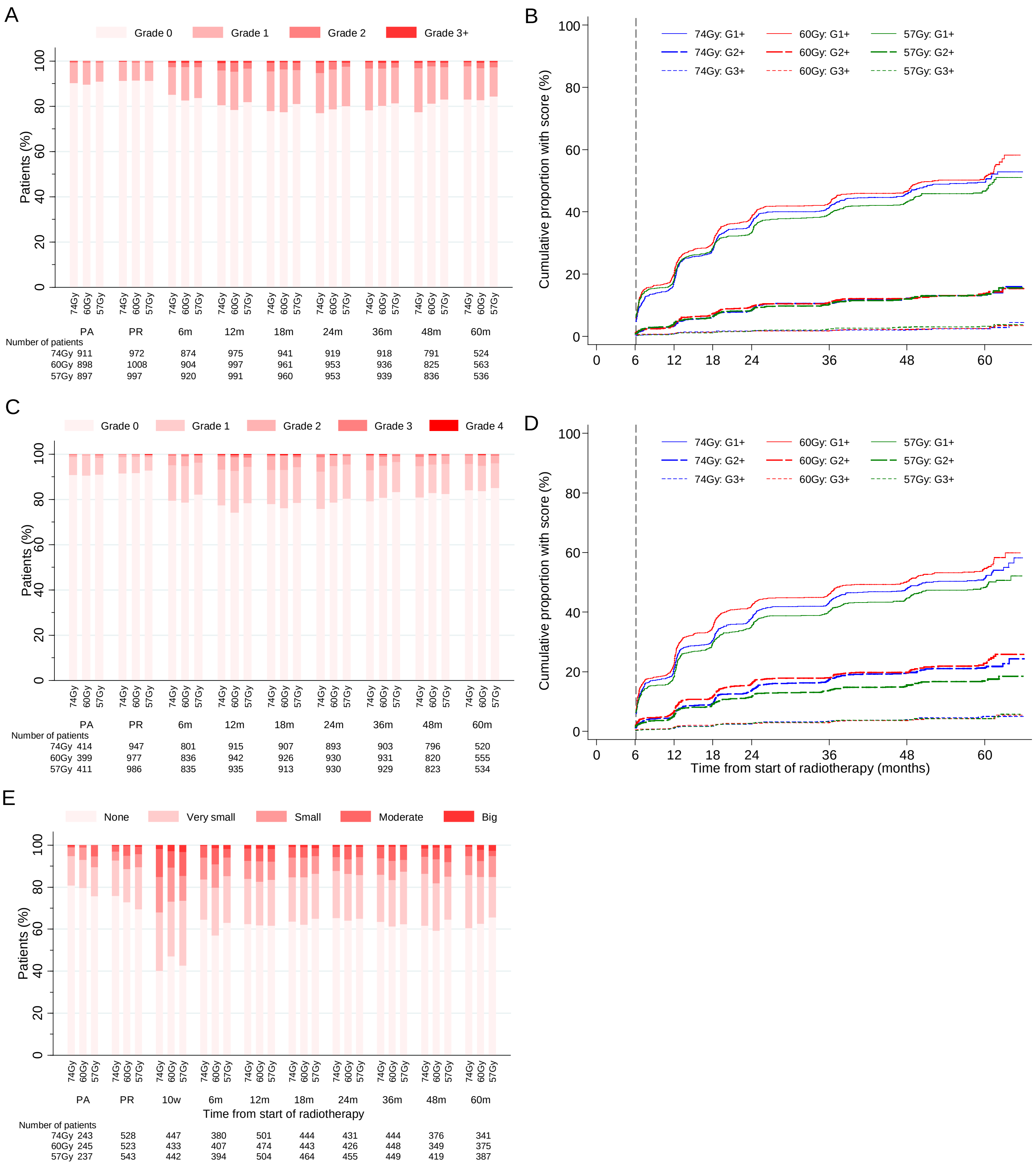
A
Grade 0
Grade 1
Grade 2
Grade 3+
Patients (%)
74Gy 60Gy 57Gy
PA PR 6m 12m 18m 24m 36m 48m 60m
Number of patients
74Gy 911 972 874 975 941 919 918 791 524
60Gy 898 1008 904 997 961 953 936 825 563
57Gy 897 997 920 991 960 953 939 836 536
B
74Gy: G1+
60Gy: G1+
57Gy: G1+
74Gy: G2+
60Gy: G2+
57Gy: G2+
74Gy: G3+
60Gy: G3+
57Gy: G3+
Cumulative proportion with score (%)
C
Grade 0
Grade 1
Grade 2
Grade 3
Grade 4
Patients (%)
PA PR 6m 12m 18m 24m 36m 48m 60m
Number of patients
74Gy 414 947 801 915 907 893 903 796 520
60Gy 399 977 836 942 926 930 931 820 555
57Gy 411 986 835 935 913 930 929 823 534
D
74Gy: G1+
60Gy: G1+
57Gy: G1+
74Gy: G2+
60Gy: G2+
57Gy: G2+
74Gy: G3+
60Gy: G3+
57Gy: G3+
Cumulative proportion with score (%)
Time from start of radiotherapy (months)
E
None
Very small
Small
Moderate
Big
Patients (%)
PA PR 10w 6m 12m 18m 24m 36m 48m 60m
Time from start of radiotherapy
Number of patients
74Gy 243 528 447 380 501 444 431 444 376 341
60Gy 245 523 433 407 474 443 426 448 349 375
57Gy 237 543 442 394 504 464 455 449 419 387

Figure 6: Late bladder toxicity by time-point, assessment and randomised treatment group

Distribution of bladder scores with RMH (A), LENT/SOM (C), UCLA PCI/EPIC (E). Cumulative proportion of bladder scores measured with RMH (B) and LENT/SOM (D).

RMH=Royal Marsden Hospital scale. LENT/SOM=Late Effects on Normal Tissue: Subjective/Objective/Management scale. UCLA PCI=UCLA Prostate cancer index; EPIC=Expanded Prostate Cancer Index Composite.

PA=before ADT. PR=before radiotherapy. ADT= androgen deprivation therapy. RT=radiotherapy. m=months

G1+ = score of grade 1 or worse. G2+ = score of grade 2 or worse. G3+ = score of grade 3 or worse.

Very small+ = score of very small, small, moderate or big bother. Small+ = score of small, moderate or big bother. Moderate+ = score of moderate or worse bother.

Late toxicity data has been included in analyses if was reported within 6 weeks of the 6 month visit, 3 months of the 12-24 month visit and within 6 months of 36-60 month visit. For LENT/SOM and UCLA/EPIC PA data is included if it was reported within 3 months prior to starting ADT and within 1 month after starting ADT. For RMH, all PA data has been used. PR data is included if it was reported within 3 months prior to RT and no more than 7 days after starting RT. Time-to-event analyses use all data reported from 6 weeks prior to the 6 month visit onwards.

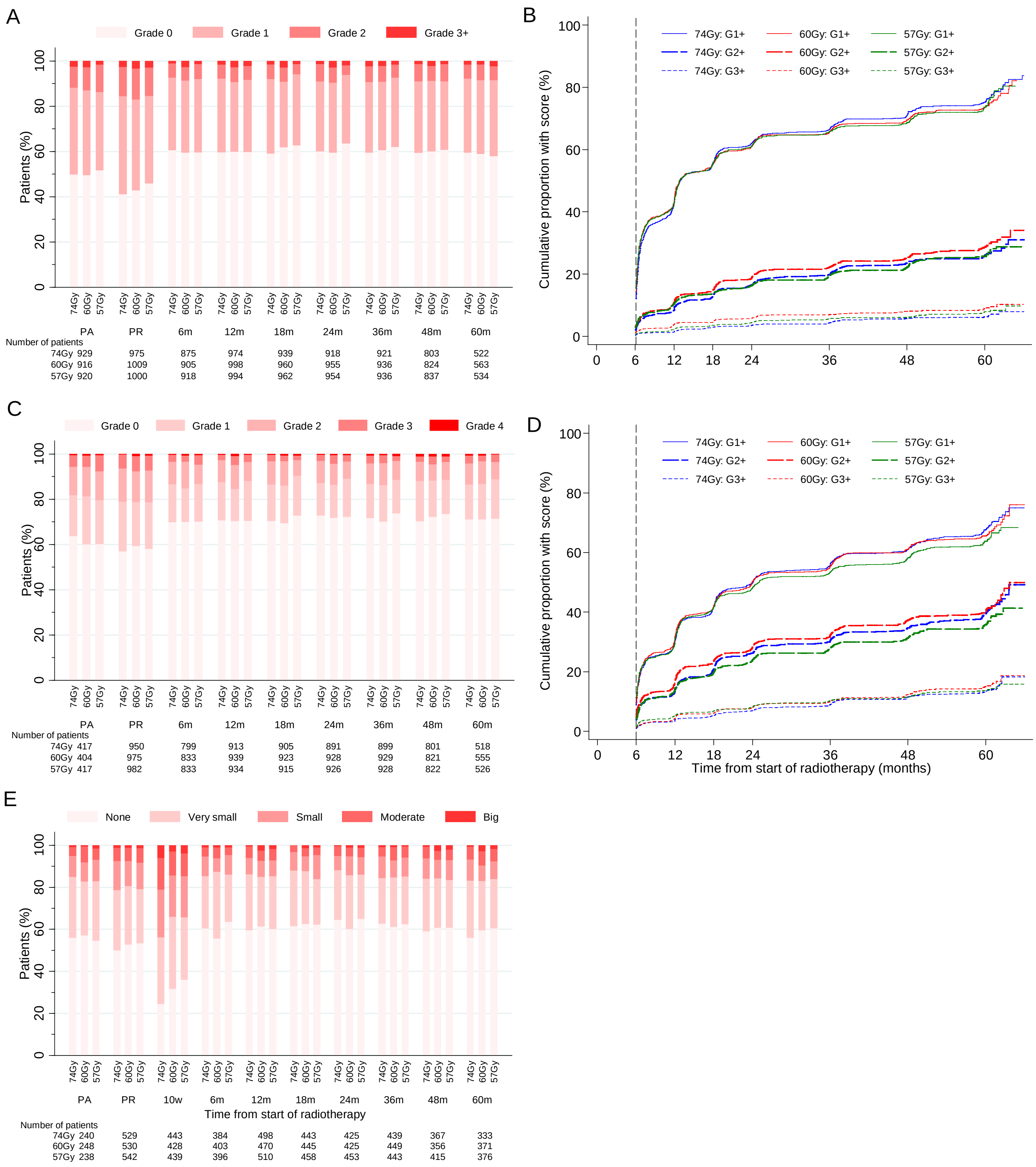

A
Grade 0
Grade 1
Grade 2
Grade 3+
Patients (%)
PA
PR
6m
12m
18m
24m
36m
48m
60m
Number of patients
74Gy 929 975 875 974 939 918 921 803 522
60Gy 916 1009 905 998 960 955 936 824 563
57Gy 920 1000 918 994 962 954 936 837 534
B
Cumulative proportion with score (%)
74Gy: G1+
60Gy: G1+
57Gy: G1+
74Gy: G2+
60Gy: G2+
57Gy: G2+
74Gy: G3+
60Gy: G3+
57Gy: G3+
C
Grade 0
Grade 1
Grade 2
Grade 3
Grade 4
Patients (%)
PA
PR
6m
12m
18m
24m
36m
48m
60m
Number of patients
74Gy 417 950 799 913 905 891 899 801 518
60Gy 404 975 833 939 923 928 929 821 555
57Gy 417 982 833 934 915 926 928 822 526
D
Cumulative proportion with score (%)
74Gy: G1+
60Gy: G1+
57Gy: G1+
74Gy: G2+
60Gy: G2+
57Gy: G2+
74Gy: G3+
60Gy: G3+
57Gy: G3+
Time from start of radiotherapy (months)
E
None
Very small
Small
Moderate
Big
Patients (%)
PA
PR
10w
6m
12m
18m
24m
36m
48m
60m
Time from start of radiotherapy
Number of patients
74Gy 240 529 443 384 498 443 425 439 367 333
60Gy 248 530 428 403 470 445 425 449 356 371
57Gy 238 542 439 396 510 458 453 443 415 376

Figure 7: Late sexual toxicity by time-point, assessment and randomised treatment group

Distribution of scores with LENT/SOM (A) and UCLA PCI/EPIC (C). Cumulative proportion measured with LENT/SOM (B) and UCLA PCI/EPIC (D).

LENT/SOM=Late Effects on Normal Tissue: Subjective/Objective/Management scale. PA=before ADT. PR=before radiotherapy. ADT=androgen deprivation therapy. RT=radiotherapy.

G1+ = score of grade 1 or worse. G2+ = score of grade 2 or worse. G3+ = score of grade 3 or worse. Very small+ = score of very small, small, moderate or big bother. Small+ = score of small, moderate or big bother. Moderate+ = score of moderate or worse bother.

Late toxicity data has been included in analyses if was reported within 6 weeks of the 6 month visit, 3 months of the 12-24 month visit and within 6 months of 36-60 month visit. PA data is included if it was reported within 3 months prior to starting ADT and within 1 month after starting ADT. PR data is included if it was reported within 3 months prior to RT and no more than 7 days after starting RT. Time-to-event analyses use all data reported from 6 weeks prior to the 6 month visit onwards.

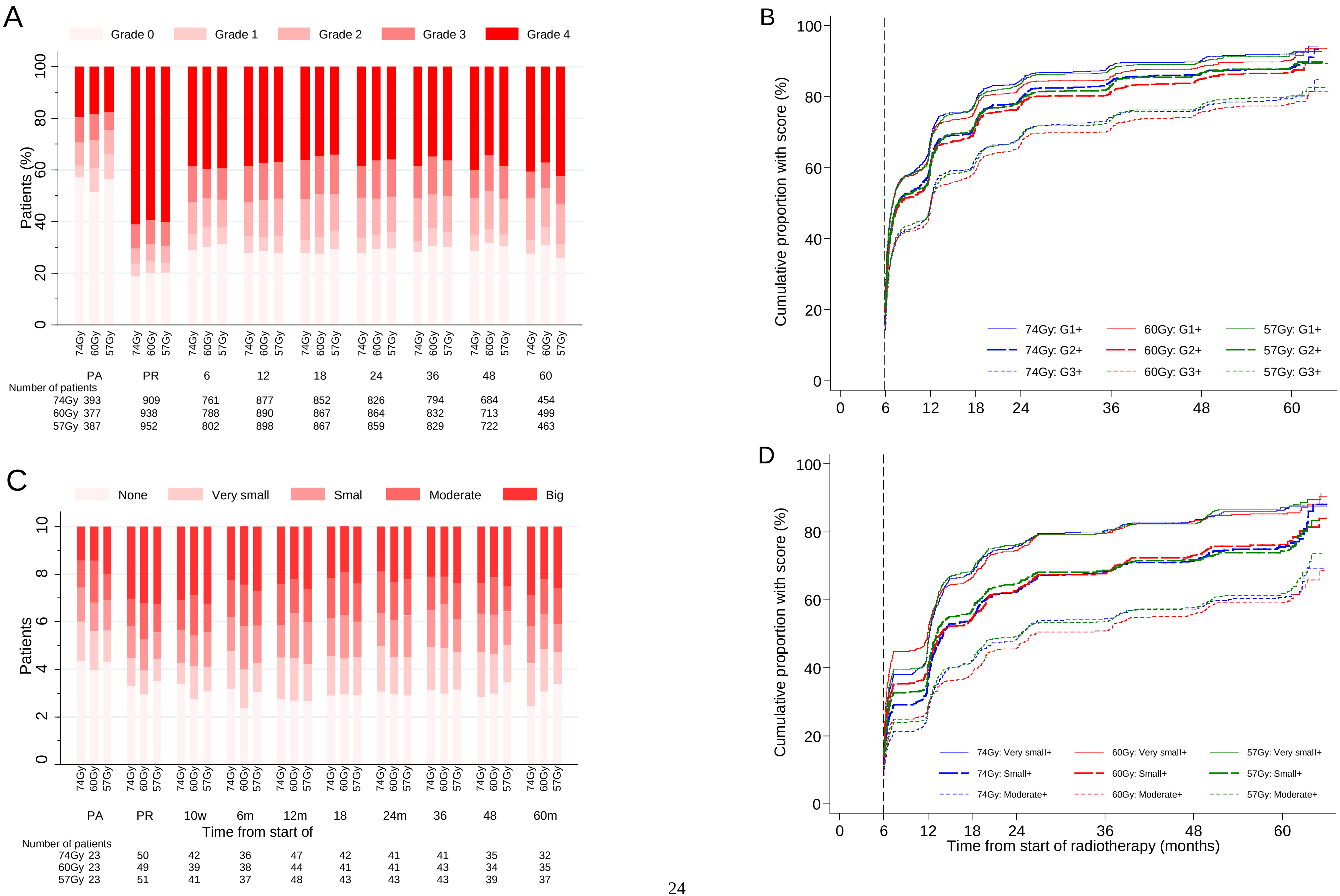
A
Grade 0
Grade 1
Grade 2
Grade 3
Grade 4
Patients (%)
0
20
40
60
80
100
74Gy 60Gy 57Gy
PA
PR
6
12
18
24
36
48
60
Number of patients
74Gy 393 909 761 877 852 826 794 684 454
60Gy 377 938 788 890 867 864 832 713 499
57Gy 387 952 802 898 867 859 829 722 463
B
Cumulative proportion with score (%)
74Gy: G1+
60Gy: G1+
57Gy: G1+
74Gy: G2+
60Gy: G2+
57Gy: G2+
74Gy: G3+
60Gy: G3+
57Gy: G3+
0
6
12
18
24
36
48
60
C
None
Very small
Smal
Moderate
Big
Patients
0
2
4
6
8
10
PA
PR
10w
6m
12m
18
24m
36
48
60m
Time from start of
Number of patients
74Gy 23 50 42 36 47 42 41 41 35 32
60Gy 23 49 39 38 44 41 41 43 34 35
57Gy 23 51 41 37 48 43 43 43 39 37
D
Cumulative proportion with score (%)
74Gy: Very small+
60Gy: Very small+
57Gy: Very small+
74Gy: Small+
60Gy: Small+
57Gy: Small+
74Gy: Moderate+
60Gy: Moderate+
57Gy: Moderate+
Time from start of radiotherapy (months)

Figure 8: Schoenfeld residuals

Biochemical/clinical failure-free survival: 60Gy vs 74Gy (A) and 57Gy vs 74Gy (B). Overall survival: 60Gy vs 74Gy (C) and 57Gy vs 74Gy (D). Recommencement of androgen deprivation therapy: 60Gy vs 74Gy (E) and 57Gy vs 74Gy (F). Development of distant metastases: 60Gy vs 74Gy (G) and 57Gy vs 74Gy (H). Disease-free survival: 60Gy vs 74Gy (I) and 57Gy vs 74Gy (J).

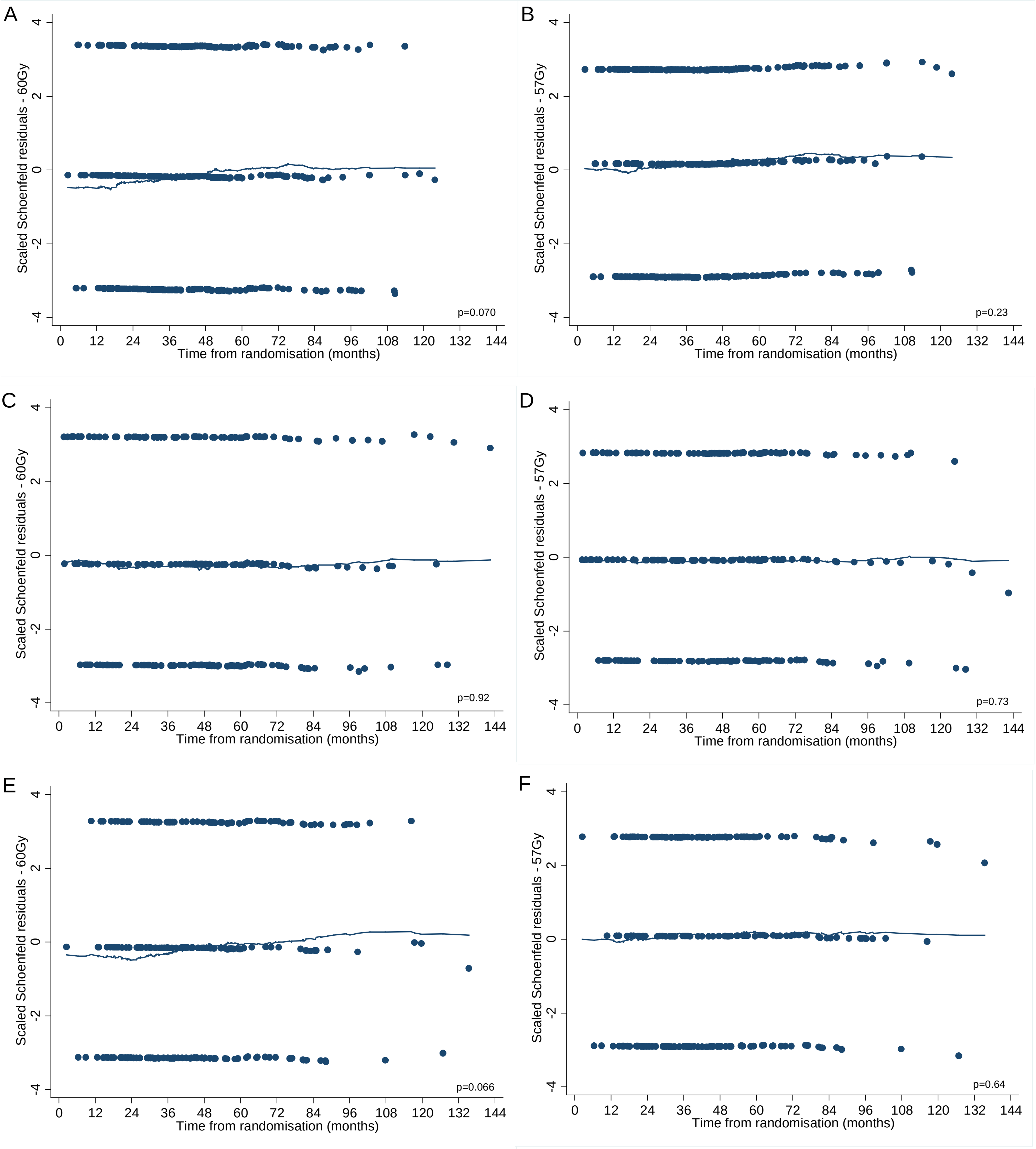

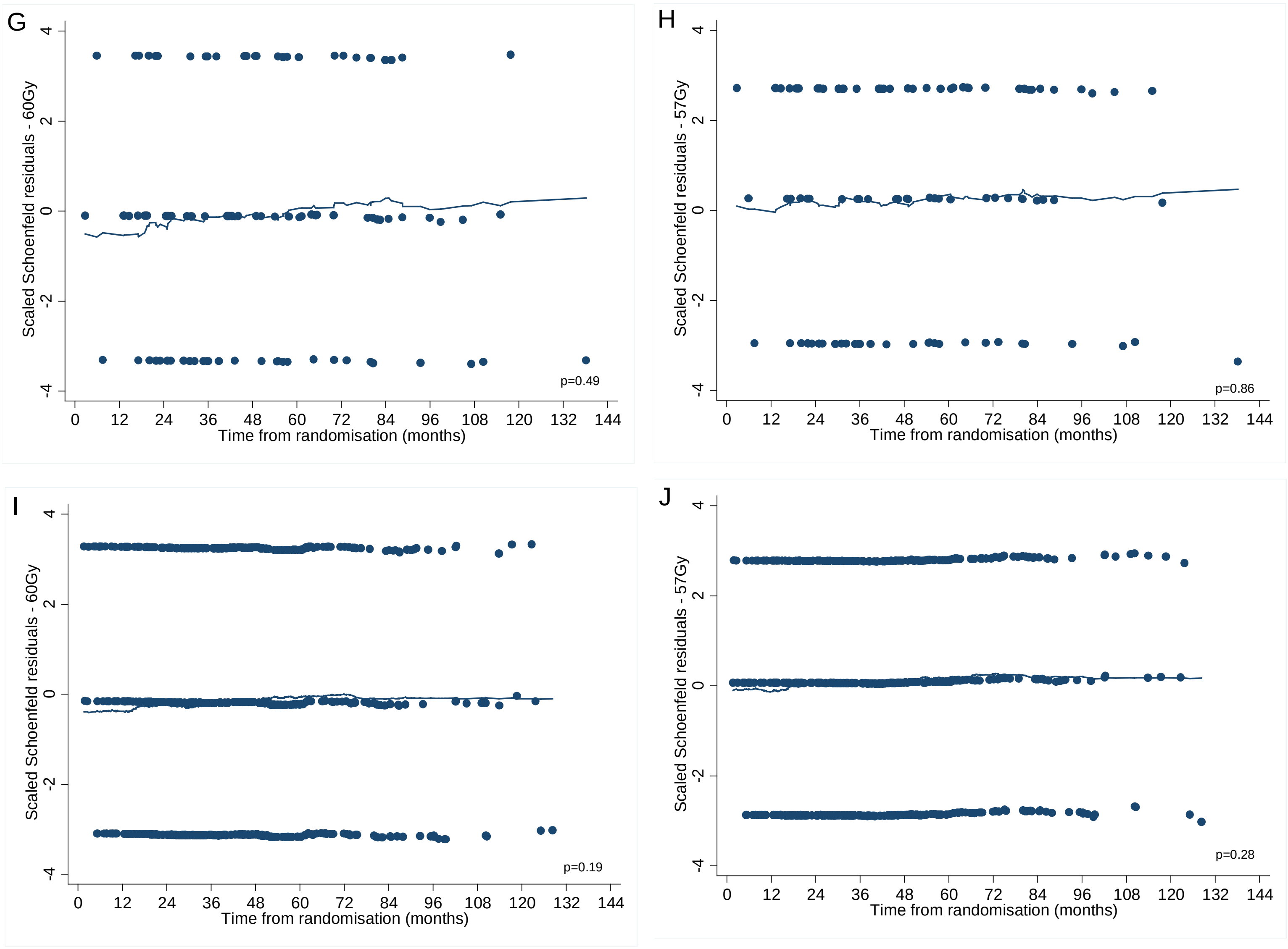
G
Scaled Schoenfeld residuals - 60Gy
p=0.49
Time from randomisation (months)
H
Scaled Schoenfeld residuals - 57Gy
p=0.86
Time from randomisation (months)
I
Scaled Schoenfeld residuals - 60Gy
p=0.19
Time from randomisation (months)
J
Scaled Schoenfeld residuals - 57Gy
p=0.28
Time from randomisation (months)

Conventional or Hypofractionated High Dose Intensity

Modulated Radiotherapy for Prostate Cancer

Protocol Version

9.2

Protocol Number:

ICR-CTSU/2006/10007

CONFIDENTIAL: Information and data included in this protocol are privileged or confidential and are the property of ICR-CTSU. No person is authorised to make this public without the written permission of ICR-CTSU. These restrictions on disclosure will apply equally to all future information supplied to you, which is indicated as privileged or confidential. However, this material may be disclosed to and used by your staff and associates as necessary to conduct the clinical study.

MREC No.: 04/MRE02/10 **ISRCTN No.: ISRCTN97182923**

General correspondence to:

ICR-CTSU*
Section of Clinical Trials
Sir Richard Doll Building
Cotswold Road
Sutton, Surrey,
SM2 5NG, UK
Tel: +44 (0) 20 8722 4183
Fax: +44 (0) 20 8770 7876
Email:CHHIP-icrctsu@icr.ac.uk

***Institute of Cancer Research**
- Clinical Trials & Statistics Unit*

Clinical correspondence to:

Professor David Dearnaley
Department of Academic Radiotherapy
The Royal Marsden Hospital
Downs Road
Sutton, Surrey, UK
SM2 5PT
Tel: +44 (0)20 8661 3271
Fax: +44 (0)20 8643 8809
Email: david.dearnaley@icr.ac.uk

This clinical trial protocol is intended to provide guidance and information only for the conduct of the CHHIP Trial in participating centres. It is not for use as a guide for the management of other patients outside of the trial.

If you have an urgent clinical query please contact:
Professor David Dearnaley on 020 8661 3271

The CHHIP trial has been scientifically approved by the Clinical Trials Awards and Advisory Committee (CTAAC) of Cancer Research UK and the Medical Research Council and is thus part of the NCRN/NCRI portfolio of prostate cancer trials.

CHHIP Trial - FINAL PROTOCOL VERSION 9.2: 10 December 2010

Approved by:

10/12/2010

Professor David Dearnaley
Chief Investigator

Date: 10/12/2010

INDEX

TRIAL SCHEMA 5
TRIAL SUMMARY 6
1. Background 7
1.1 Dose Escalation in Prostate Cancer 7
1.2 Hormone Treatment 8
1.3 Radiobiology of Prostate Cancer and Normal Tissue: Rationale for Hypofractionation 9
1.4 Rationale for Study Design 11
2. Objectives of the Study 13
2.1. To test the hypothesis that hypofractionated radiotherapy schedules for localised prostate cancer will improve the therapeutic ratio by either: 13
2.2. To limit acute and late gastro-intestinal and urological toxicity. 13
2.3. To evaluate different PSA related endpoints for local failure and distant metastases. 13
2.4. To extend the database of patients treated to escalated doses with dose volume histograms (DVHs) of normal tissues at risk and to relate these to common toxicity endpoints. 13
2.5. To develop a model to estimate normal tissue complication probability (NTCPs) of rectum and bladder for hypofractionated as well as conventional dose escalated radiotherapy schedules. 13
3. Trial Design 13
Figure 1 13
4. Patient Selection and Eligibility Criteria 14
5. Study Endpoints 15
5.1 Primary*:* 15
5.2 Secondary*:* 15
6. Randomisation, registration and treatment allocation 15
6.1 Registration & Randomisation 15
6.2 Allocation of treatment 15
7. Investigations and Assessment Procedures 16
7.1 Initial Assessment 16
7.2 During Hormone Therapy 17
7.3 During Radiotherapy 17
7.4 Post-radiotherapy treatment Follow-Up 17
7.5 Assessment of Disease Recurrence 17
7.6 Quality of Life Instruments 18
8. Treatment 19
8.1 Hormone Therapy 19
8.2 Radiotherapy Planning and Treatment 19
8.3 CT Planning for Radiotherapy 19
8.4 Target Volumes and Dose Assessment Points 20
Figure 2 22
Table 2 Summary of GTV, CTV and PTV Definitions and Dose Levels in Different Treatment Groups 23
Table 3 Outlining and Target Isodoses 24

Figure 3		25
8.5	Normal Tissue Contouring and Dose Volume Histograms	26
Table 4	Normal Tissue Dose Constraints	26
8.6	Radiotherapy Treatment Planning	27
8.7	Dose Constraints	28
8.8	Treatment Delivery	29
8.9	Verification and Accuracy	30
9.	Adverse Events (AE) / Serious Adverse Events (SAE)	31
9.1	Definition of an Adverse Event	31
9.2	Definition of Serious Adverse Events	31
9.3	Reporting of Adverse Events	32
9.4	Expedited reporting of SAEs	32
9.5	Reporting related and unexpected SAEs	33
9.6	SAE follow up	33
10	Statistical Considerations	33
10.1	Part I: Two Centres	33
10.2	Part II: Multicentre	34
10.3	Part III: Multicentre	35
Table 5		35
10.4	Part IV: Multicentre	37
11	Research Governance	37
11.1.1	Trial Administration and Logistics	37
11.1.2	Participating centres responsibilities	37
11.1.3	Quality Assurance of Radiotherapy Delivery	38
11.2	Investigator training	38
11.3	Case Report Forms	38
11.4	Protocol compliance/on site Monitoring	39
11.5	Trial Management	40
11.5.1	Trial Management Group	40
11.5.2	Trial Steering Committee	40
11.5.3	Data Monitoring Committee	41
11.6	End of Study	41
11.7	Archiving	41
11.8	Publishing policy	42
12	Confidentiality and Liability	42
12.1	Liability/Indemnity/Insurance	42
12.2	Patient Confidentiality	42
13	Ethical Considerations	43
14	Withdrawal of patients from study treatment	43
15	Financial Matters	44
16	Associated studies	44
16.1	Quality of Life (QOL)	44
16.2	Health Economics	46
16.3	Translational studies	46
17.	References	48
APPENDIX 1 – Image Guided Radiotherapy (IGRT) Sub-study		53

TRIAL SCHEMA

CHHIP TRIAL

Initial Assessment

- Biopsy-TURP/TRB
- Blood test-PSA, FBC, Biochemistry
- Local staging-rectal exam
- Lymph node staging-MRI/CT
- Metastases staging-Bone scan
- Pre-treatment symptom and Quality of Life assessments[+#]

↓

Hormone Treatment

Total 3-6 months only *+

↓

Randomisation

Group 1	Group 2	Group 3
74Gy/37#	60Gy/20#	57Gy/19#

↓

Pre-radiotherapy Assessment

- Rectal exam **
- Blood test
- Symptoms and QoL assessments[#]

↓

Radiotherapy

- Acute toxicity at Weeks 1-8, 10, 12 & 18[##]
- PSA at Weeks 10 & 18
- Rectal exam** at Week 18

↓

Post-radiotherapy

- PSA- 6 mth to 5 yrs 6 monthly intervals then annually
- Late side effect (RTOG/LS), rectal exam** & QoL from 6mth to 2 yrs 6 monthly intervals, Yrs 2-5 annually. [#]
- Disease recurrence assessment

↓

End points

- Acute side effects
- Late side effects
- Biochemical (PSA) recurrence
- Distant recurrence
- Cause specific survival
- Overall survival

* May be omitted for patients with good risk disease (PSA$\leq$ 10ng/ml <u>and</u> Gleason score $\leq$ 6 <u>and</u> T1c/T2a)

** Rectal examination may be omitted if the previous examination was normal <u>and</u> PSA $\leq$ 1.5ng/ml <u>and</u> no symptoms suggesting recurrence. [1]

\+ Patients who have already commenced hormonal therapy remain eligible but pre-hormone symptom scores and hormone measurement will be omitted

[#] The QoL sub-study has closed to patient recruitment. [##] For IGRT patients only.

TRIAL SUMMARY

Phase III randomised trials using conformal radiotherapy (CFRT) have shown that increasing radiation dose improves the control of localised prostate cancer and can be delivered safely without an increase in radiation related side effects. Recent studies on the radiobiology of prostate cancer have suggested that shorter courses of radiotherapy giving higher doses at each treatment (hypofractionated radiotherapy) may give improved cancer control for the same level of radiation related side effects. If this suggestion were to be confirmed, then treatments would become more convenient for patients for example 20 treatments over four weeks compared to 37 treatments over seven and a half weeks and radiotherapy resources would be better utilised. Intensity modulated radiotherapy (IMRT) techniques can now be designed which achieve a further improvement in conformality and normal tissue avoidance compared to CFRT. Suitable IMRT techniques will be used in this trial. The study will be undertaken in three stages. Part 1 is a randomised pilot study which will obtain preliminary data on side effects and has been undertaken in two centres (Royal Marsden Hospitals and Clatterbridge Cancer Centre); Part 2 has been expanded to include eleven centres and is powered to formally compare the side effects of the three treatment schedules; Part 3 has been approved as a national multi-centre study and will be powered to compare treatment efficacy. Part 1 of the study has been run by the Academic Unit of Radiotherapy at the RMH and is supported by the Unit's CR UK Programme Grant; Part 2 has been supported by the Dept of Health and Southern Prostate Cancer Collaborative to facilitate generalisibility of the hypofractionated and IMRT techniques. Part 3 is supported by CTAAC with quality assurance from the Dept of Health and NCRN.

1. Background

1.1 Dose Escalation in Prostate Cancer

Radiotherapy is one of the curative treatment options for localised prostate cancer [2, 3]. Considerable advances in radiation technology over the last decade have led to the development of conformal radiation treatments which more closely match the high dose volume to the tumour target whilst reducing the radiation to dose limiting normal tissues [4]. The potential advantages of these techniques is to enable a reduction in radiation related side effects as well as permitting the safe delivery of high doses of radiation which might improve treatment efficacy. Institutional experiences and results from phase I/II studies suggests that both these goals may be achievable [5-7] and that dose/response relationships exist for tumour control as well as dose/volume/complication relationships for the development of late normal tissue damage.

In a phase III randomised trial [8] we compared conventional and conformal radiotherapy (CFRT) at a standard dose of 64 Gy and showed a significant reduction in the dose limiting late side effect of proctitis using CFRT but no detriment in disease control. Three phase III trials using conformal photon beam treatment have reported gains in overall PSA control of between 6% and 12% using higher doses of radiation [9, 10, 72]. In the MD Anderson trial, which compared 70 and 78Gy, benefit (19% PSA control advantage) was restricted to men with a presenting PSA >10ng/ml [10]. The Royal Marsden Hospital (RMH) study compared 64 and 74Gy in combination with neoadjuvant androgen suppression [9]. The higher dose gave a 12% advantage in PSA control. Late morbidity was increased in high dose groups in both trials. In the recently reported Dutch multicentre trial in which 664 men were randomised to receive treatment with 78Gy or 68Gy, there was a 10% PSA control advantage for the higher dose, which was most clearly seen in men with intermediate risk disease (HR 0.6). Preliminary results using a proton beam boost (PROG 95-09) comparing doses of 70.2Gy equivalent and 79.2Gy equivalent suggests an 18% PSA control advantage in both low and intermediate risk groups [11]. These results build on the improvements in PSA control rates that have been previously reported in phase II studies in larger groups of men. [5-7, 12, 13]. For example, the Memorial Sloan Kettering Group have reported outcome from 1,100 men comparing doses in the range of 64 to 70Gy and 76 to 86Gy [14]. Using clinical stage, histological grade and

presenting PSA to define prognostic groups showed 5 year actuarial PSA control rates of 77% vs 90% (p= .05) for the most favourable group, 50% vs 70% (p= .001) for the intermediate group and 21% vs 47% (p= .002) for the unfavourable group treated to lower or higher doses respectively. A critical issue is whether or not PSA control will clearly relate to disease recurrence or to overall survival. A retrospective analysis from the Radiotherapy and Oncology Group (RTOG) suggests that dose escalation may indeed be related to improved survival. In their study, which included 1465 men treated in 4 protocols between 1975 and 1992, men with high grade cancers who received higher radiation doses (≥66Gy versus <66Gy) had a 20% lower risk of death from prostate cancer and a 27% reduction in overall mortality. This benefit was not seen in men with well or moderately differentiated cancers [15]. Over 3,000 men will be randomised in ongoing phase III studies of dose escalation in the UK (MRC RT01 trial), The Netherlands, France and N. America. The MRC RT01 trial completed recruitment of over 850 patients in December 2001 [51]. The remaining studies give doses of 68-73Gy in the control groups and 78-82Gy in the escalated dose group. As a result of the advantage demonstrated in the Royal Marsden/ICR trial and MD Anderson trials, 74Gy will become the standard dose for men treated in this study.

1.2 Hormone Treatment

An alternative strategy to improve the treatment results of radiation therapy is to use short or longer periods of adjuvant androgen suppression/blockade. Potential advantages of combined modality treatment include an additive or synergistic effect on tumour cell kill, a reduction in radiation target volume and reduction in the development of metastases. Phase III randomised trials have shown benefit for both short [16-19] and longer course hormonal therapy [20-23]. For short course (3-6 months) treatment long-term PSA control rates improve by 14-24% and with long term treatment (≥2 years) PSA control rates improved by 21% -31% with benefits in metastases-free, cause-specific and overall survival. All studies suggest improved outcome for long course treatment in men with locally advanced high grade cancer [23, 24]. Intermediate risk groups benefit from short course treatment but it is not yet certain if good prognosis patients benefit from adjuvant hormonal therapy in addition to high dose radiotherapy - NCRN approved EORTC Trial 22991 is addressing this question.

Several groups have measured the reduction in prostate and prostate target volume after initial hormone treatment which varied between 25-41% and showed a complementary increase in the sparing of rectum and bladder when initial hormone treatment was combined with CFRT [25-27].

1.3 Radiobiology of Prostate Cancer and Normal Tissue: Rationale for Hypofractionation

Recently there has been considerable discussion concerning the radiobiology of prostate cancer's response to irradiation [28-36]. In general, increased radiation fractionation provides an increasing therapeutic advantage between tumour control and late treatment related side effects, in that fractionation spares late responding normal tissues more than tumours because tumours normally respond as early responding tissue [37]. This sensitivity to change in fractionation is expressed mathematically in the linear-quadratic formalism and is quantified by the alpha-beta ratio [37]. In general, late responding normal tissues have a low alpha-beta ratio (usually taken as approximately 3 Gy) whereas early responding tissues responsible for acute radiation reactions and most cancers have a high alpha-beta ratio (usually 8-10 Gy). Fractionation spares tissues with a low alpha-beta ratio and radiotherapy schedules are designed so as to keep late radiation reactions at an acceptable level. For this reason, most cancers are treated with 1.8 - 2Gy daily fractions over a period of 6-8 weeks. However, studies deriving the alpha-beta ratio for prostate cancer from low dose rate brachytherapy treatments have suggested the alpha-beta ratio is 1.5 Gy (95% confidence intervals 0.8 - 2.2Gy) [38] and 1.49 Gy (95% confidence intervals 1.25 - 1.76) [29]. A further analysis using external beam radiotherapy with high dose brachytherapy estimated the alpha-beta ratio at 1.2Gy (95% confidence intervals .03-4.1Gy) [34]. If these estimates are accurate, they would predict that hypofractionated schedules for prostate cancer should produce tumour control and late treatment related sequelae that are at least as good or better than those currently achieved with currently standard schedules using 1.8-2.0Gy daily fractions. However, different assumptions in the models used for calculating the alpha-beta ratios can lead to estimates as high as 10Gy [28] and values of 8.5 Gy and 15.5 Gy have recently been derived by incorporating hypoxia into the modelling process [35].

Clinically hypofractionated external beam radiotherapy has been used for many years in the UK for a variety of malignancies, predominantly as a result of limited resources. In the past satisfactory results were claimed using a variety of hypofractionated treatment schedules for

prostate cancer varying from 50Gy in 20 fractions over 4 weeks [39], 50Gy in 16 fractions over 21 days [40] and 36Gy in 6 treatments over five weeks [41]. Many centres in the UK continue to use 4 week radiotherapy schedules using total doses of between 50 and 55Gy.

Contemporary reports of hypofractionated schedules are limited. A phase III trial in 936 men has compared 52.5Gy in 20 fractions with 66Gy in 33 fractions. Preliminary results appeared to show a 7% reduction in PSA control rate (49% vs. 56%) in the 20 fraction arm with hazard ratio for failure (short to long) of 1.20 (95% CI 1.0 to 1.44). Late toxicity was similar in the two arms (Grade 3/4 = 3%) [42]. A second, small, randomised controlled trial including 120 men compared a dose of 64Gy in 32 fractions with 55Gy in 20 fractions. After median follow up of 44 months, 4 year PSA control rates were similar (86.2% vs. 85.4% for hypo and standard fractionation respectively); there was a slight excess of rectal bleeding in the hypofractionated group [43]. Comparison of a large single institute series in which 705 men were treated to a dose of 50Gy in 16 fractions gave similar PSA control rates to schedules of 65-70Gy in 1.8-2.0Gy fractions with a low toxicity profile [44]. All of these studies are compatible with an α/β ratio for prostate cancer of ≤1.5 -3.0Gy [45]. Additionally, a preliminary report from the USA [46] suggested that a dose of 70Gy in 2.5Gy fractions was at least as effective as 78Gy in 2Gy fractions. Presently there are no long-term data using higher dose hypofractionated radiotherapy. Phase I studies using 3Gy fractions have recruited in Manchester (57Gy, 60Gy) [47], Toronto (up to 66Gy) (personal communication) and Japan (69Gy) [48].

The alpha-beta ratio for late reactions in normal tissues is usually taken as 3Gy for skin, mucosa and bowel. However, human data is quite imprecise. For example, the alpha-beta ratio for late telangiectasia following breast irradiation is 2.8 (95% confidence intervals 1.7-3.8), for breast fibrosis the alpha-beta ratio has been reported as 1.9 (95% confidence intervals 0.8-3.0), and for bowel stricture and perforation following pelvic treatment the alpha-beta ratio is probably between 2.2 Gy and 8Gy. [37, 49]. However, a recent review suggests that the alpha-beta ratio for radiation induced proctitis may be relatively high at 5.4Gy (±1.5Gy) [50]. There is little information concerning the effect of overall treatment time on the development of late radiation reactions and using schedules that are only modestly shortened it may be that no overall time factor is required for either tumours or late complications [30]. The situation is also uncertain for acute reactions. Although the overall reduced dose used in hypofractionated

schedules would be expected to lower side effects if the overall treatment time was kept constant (e.g. by treating 5 x fortnight), decrease in overall time (treatment acceleration) might increase side effects.

If the radiobiological predictions of a low alpha-beta ratio for prostate cancer are correct, such shortened schedules may be associated with improvements in tumour control for a given level of radiation related side effects. If this is the case, then such schedules should become the standard approach to treatment as they would be more convenient for patients and make better use of radiotherapy resources. To date, no Phase III study of dose escalated conformal or intensity modulated radiotherapy using hypofractionated schedules has been performed.

1.4 Rationale for Study Design

We want to test whether there is an improvement in the therapeutic ratio using an hypofractionated radiotherapy schedule in prostate cancer. The study design is based on the biological hypothesis that the α/β ratio for prostate cancer is low (<3Gy). Two different strategies can be used to select appropriate dose levels in the hypofractionated groups. The first would be to assume a low alpha-beta ratio of 1.5Gy and then to calculate the iso effective dose required using any hypofractionated schedule. It would be predicted that both late and acute reactions would be reduced (assuming alpha-beta ratio of 3 Gy and 10 Gy respectively). The risk with this approach is that the tumour will be undertreated if the alpha-beta ratio were to be higher. The second strategy would be to aim for an iso-effective dose for late normal tissue complications assuming an alpha-beta ratio of 3 Gy. If the alpha-beta ratio for tumour (e.g. 1.5Gy) is lower than that for normal tissues (e.g. alpha-beta ratio = 3Gy) then if treatment groups are iso-effective for late normal tissue damage the hypofractionated schedule would have a higher tumour control probability. A difficulty arises, however, because of the imprecisely known alpha-beta ratios for both tumour and late responding normal tissues.

We have favoured a mixed strategy as this acknowledges the uncertainties in alpha-beta ratio for both prostate cancer and normal tissues. As 4 week schedules have been used in the UK and are familiar to clinicians, and some pilot data is already available, we have chosen to compare 4 week schedules with the standard 7.5 week treatment. A 3 group randomisation is preferable so that two points on the experimental hypofractionated schedule dose complication

response curve can be observed which should allow extrapolation to an iso-effective dose for tumour control compared to conventional 2Gy fractionation schedules. Table 1 shows the predicted 2Gy equivalent doses for a range of alpha-beta ratios comparing conventional 74Gy (2Gy fractions), high dose hypofractionated schedules, as well as previously studied "standard" hypofractionated treatments. The calculated 2Gy equivalent doses using a 20 fraction schedule are 61.1Gy and 58.0Gy for alpha/beta ratio of 3.0 and 1.5Gy respectively. Practically 3Gy fractionation schedules are attractive, and schedules of 60Gy in 20 fractions, and 57Gy in 19 fractions will be compared with standard treatment of 74Gy in 37 fractions; the hypofractionated schedules would be iso-effective for alpha beta ratios of 2.5Gy and 1.5Gy respectively. Assuming the alpha-beta ratio for normal tissues is 3Gy and the alpha-beta ratio 1.5Gy for prostate cancer, the hypofractionated group would show a therapeutic gain in that tumour control would improve for an iso-effect on normal tissues. If the alpha-beta ratio for prostate cancer is over 3Gy, however, there will be a relative dose detriment for tumour control for both groups and if the alpha-beta ratio for late reacting normal tissues were under 1.5Gy then there would be an increase in normal tissue complication rate for both groups. However, both hypofractionated groups are treated to a total dose in excess of what has been given using conventional rather than conformal radiotherapy techniques (50 - 55Gy in 20 fractions or 50Gy in 16 fractions) and it is anticipated that IMRT techniques will make a significant reduction on normal tissue complication rates. As above, if acute side effects were found to be dose limiting in the pilot study, overall treatment time could then be lengthened.

Table 1 Fractionation Schedules in Prostate Radiotherapy

Dose (Gy)	Dose/F (Gy)	No. F	2Gy Equivalent Dose α/β Ratio					
			1.0	1.5	2.0	3.0	5.0	10.0
50	3.13	16	69	66	63	60	58	54
55	2.75	20	68	66	65	64	61	58
57	3.0	19	76	74	71	68	65	62
60	3.0	20	80	78	75	72	68	66
70	2.5	28	82	80	78	77	75	73
70.2	1.8	39	66	66	66	68	68	69
77.4	1.8	43	72	73	74	74	76	78

2. Objectives of the Study

2.1. To test the hypothesis that hypofractionated radiotherapy schedules for localised prostate cancer will improve the therapeutic ratio by either:

Improving tumour control or
Reducing normal tissue side effects

2.2. To limit acute and late gastro-intestinal and urological toxicity.

2.3. To evaluate different PSA related endpoints for local failure and distant metastases.

2.4. To extend the database of patients treated to escalated doses with dose volume histograms (DVHs) of normal tissues at risk and to relate these to common toxicity endpoints.

2.5. To develop a model to estimate normal tissue complication probability (NTCPs) of rectum and bladder for hypofractionated as well as conventional dose escalated radiotherapy schedules.

3. Trial Design

Patients will be randomised between conventional radiotherapy fractionation using a total dose of 74 Gy in 37 fractions over 7.4 weeks using conformal and intensity modulated radiotherapy techniques and the experimental groups of 57Gy in 19 fractions over 3.8 weeks and 60Gy in 20 fractions over 4 weeks (Figure 1).

Figure 1

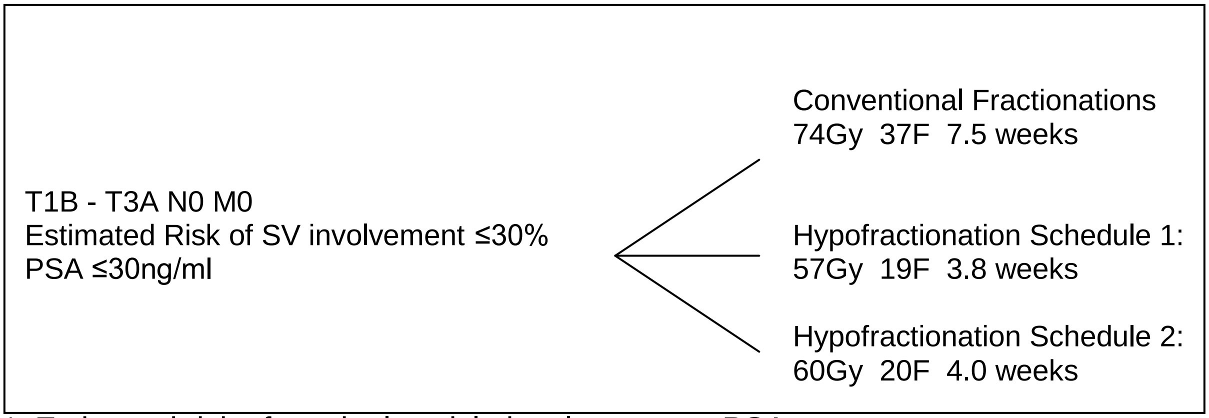

* Estimated risk of seminal vesicle involvement = PSA + ([Gleason score -6] x10) [52]

4. Patient Selection and Eligibility Criteria

Inclusion:

- Histologically confirmed, previously untreated locally confined adenocarcinoma of the prostate
- Clinical stage T1b – T3a, N0, M0. (1997 TNM system)
- PSA ≤ 30 ng/ml
- Estimated risk of seminal vesicle involvement* ≤ 30%
- WHO performance status 0 or 1
- Normal blood count (Hb >11g/dl, WBC > 4000/mm^3, platelets >100,000/mm^3)
- Written informed consent

Exclusion:

- Patients with T3 cancers with Gleason Sum ≥8 cancers are ineligible
- Prior pelvic radiotherapy or radical prostatectomy
- Previous androgen deprivation
- Life expectancy <10 years
- Previous active malignancy within the last five years other than basal cell carcinoma
- Co-morbid conditions likely to impact on the advisability of radical radiotherapy (e.g. previously inflammatory bowel disease, previous colorectal surgery, significant bladder instability or urinary incontinence)
- Bilateral Hip prostheses or fixation which would interfere with standard radiation beam configuration

* Estimated risk of seminal vesicle involvement[44]= PSA +([Gleason score-6] x10)(i.e. for the purposes of this study if Gleason score≤6 then PSA must be ≤30ng/ml: if Gleason score= 7 then PSA must be≤20ng/ml : if Gleason score=8 then PSA must be≤10ng/ml: if Gleason score= 9 or 10, patient is ineligible).

5. Study Endpoints

5.1 Primary*:*
Freedom from biochemical (PSA) failure or prostate cancer recurrence.
Biochemical failure defined according to Phoenix consensus guidelines[75] as Nadir + 2ng/ml.
The Nadir PSA level is the lowest value recorded at any time after commencement of hormone and/or radiotherapy treatment.

5.2 Secondary*:*

- Acute and late radiation induced side effects
- Development of metastases
- Recommencement of hormonal treatment for disease occurrence
- Cause specific and overall survival
- Aspects of quality of life and health economics
- Models of normal tissue and tumour control

6. Randomisation, registration and treatment allocation

6.1 Registration & Randomisation

Patients are initially registered to the trial after obtaining informed consent. Ideally, patients should be registered prior to commencing hormone therapy. To register a patient complete Registration forms 1 and 2 and fax to the CHHIP Trial Team on **020 8722 4368**. The patient will be allocated a unique registration number.

Patients should be randomised as close to the start of radiotherapy as possible. Patients are randomised by calling the ICR-CTSU. The patient's registration number will be required at the time of randomisation.

Patients are randomised by telephone through the ICR-CTSU

Tel: 020 8643 7150 (09.00 – 17.00 Monday to Friday)

The caller will be given the patient's unique trial identification number (Trial ID) and treatment allocation.

6.2 Allocation of treatment

Treatment allocation will be 1:1:1 and will use computer generated random permuted blocks. Randomisation will be stratified by treating centre and risk group (low, intermediate or high).
A letter confirming randomisation will be sent to the centre to confirm treatment allocation.

7. Investigations and Assessment Procedures

7.1 Initial Assessment

All patients are required to undergo the following pre-randomisation investigations:

- clinical history and physical examination
- histological evaluation of prostate biopsy to be assessed using the Gleason scoring system
- staging procedure:-
  - * local tumour staging - record clinical results of rectal exam and findings of TRUS or MRI (body or endorectal coil)
  - * lymph node staging - record findings of CT or MRI (body coil)
  - * metastases staging - bone scan (maybe omitted if PSA ≤10ng/ml <u>and</u> Gleason Score ≤7)
- full blood count and biochemistry to include creatinine, alkaline phosphatase, PSA, testosterone**, FSH, LH.
- symptoms** - bowel, urinary symptoms and potency will be recorded using RTOG, LENT-SOM, and Quality of Life instruments

** Patients who have already commenced hormonal therapy remain eligible but pre-hormone symptom scores and hormone measurement will be omitted

For Part III, patients will be stratified by risk group as defined by NCCN Practice Guidelines in oncology. [74]

Three groups of patients will be defined as:

- Low risk prostate cancer (Group L)
  ▭clinical stages T1b/c or T2a, with PSA ≤10 and Gleason score ≤6

- Intermediate risk prostate cancer (Group I)
  ▭ presence of any of the following*: PSA 10-20, Gleason Score 7, clinical stage T2b

- High risk prostate cancer (Group H)
  ▭presence of any of the following: PSA >20, Gleason Score 8-10, clinical stage T3a

▭ TNM 1997 Classification

* Excluding patients with any high-risk feature

7.2 During Hormone Therapy

- PSA to be measured at 6 weeks and 12 weeks (prior to commencement of radiotherapy)
- Bowel, urinary symptoms and potency will be assessed using RTOG and LENT-SOM
- Rectal examination* prior to radiotherapy

7.3 During Radiotherapy

• Acute toxicity assessments (RTOG)**	Weeks 1-8, 10, 12 and 18.
• PSA	Weeks 10 and 18
• Rectal examination*	Week 18

7.4 Post-radiotherapy treatment Follow-Up

- PSA - 6 months (26 weeks after commencement of RT), 12 months, 18 months, 24 months, Years 2-5: 6 monthly intervals; Years 6-15: annual intervals
- Hormones - 12 months after commencement of RT
- Late side effect assessment (using RTOG and LENT-SOM) - 6 months (26 weeks after commencement of RT), 12 months, 18 months, 24 months; Years 2-5: annual intervals
- Rectal examination* - 6 months (26 weeks after commencement of RT), 12 months, 18 months, 24 months; Years 2-5: annual intervals
- Health Resource questionnaire - 6 months (26 weeks after commencement of RT), 12 months, 18 months, 24 months; Years 2-5: annual intervals

*Rectal examination may be omitted if the previous examination was normal <u>and</u> PSA≤1.5ng/ml <u>and</u> no symptoms suggesting recurrence. [1]

**For IGRT patients only.

7.5 Assessment of Disease Recurrence

Full assessment of disease will be undertaken if there is significant clinical or biochemical evidence of disease recurrence that will include CT or MR of the pelvis and bone scan. There should be clinical evidence of recurrence or PSA levels should be: (a) at least 10ngs/ml and (b) >50% of presenting PSA level to trigger re-evaluation [53] unless (c) the PSA doubling time is ≤6 months or high grade (Gleason 8-10) disease was initially present and PSA >5ngs/ml.

Alternatively, reassessment should be undertaken with lower PSA levels if the decision is made to recommence hormone therapy.

7.6 Quality of Life Instruments

This sub-study has now closed to recruitment.

The instruments used will be the FACT-P (Prostate) and UCLA/RAND Prostate Cancer Index. However, the UCLA/RAND Prostate Cancer Index will be replaced with the updated version. The other instruments to be used will be the combined versions of the Expanded Prostate Cancer Index Composite (EPIC) plus SF-12 questionnaires. The Quality of Life sub-study will be discussed further in section 16.

It is essential to explain to the patient that the QL questionnaire is an important part of their assessment in the trial, and that all sections and questions should be answered even if the patient feels them to be irrelevant.

8. Treatment

8.1 Hormone Therapy

Androgen deprivation will be achieved using LHRH agonists in conjunction with initial cyproterone acetate* (CPA) to prevent ‘flare’ phenomenon. CPA may commence on or during the week before the day of the first LHRH agonist injection and should be given for at least two weeks after the LHRHa injection. Monthly depot injections of LHRH analogues should be used as 3 monthly depot preparations have a prolonged median duration of action. The duration of androgen deprivation should be at least three months (maximum six months) prior to commencement of radiotherapy and should continue until the end of radiotherapy treatment. The last monthly depot injection should be given within 1 week of the start or during radiotherapy. Alternatively bicalutamide 150mg daily may be given and should continue for 12 weeks[+] after the start of radiotherapy. Hormone treatment may be omitted for patients with good risk disease (T1c/T2a and Gleason score ≤6 and PSA ≤10ng/ml).

*Equivalent alternatives are permissible.

[+]This aims to mimic the duration of action of monthly LHRHa depot preparations. If troublesome gynaecomastia or breast pain develops, Tamoxifen 10mgs once or twice weekly may be given.

8.2 Radiotherapy Planning and Treatment

Following randomisation patients will be allocated to one of the three treatment groups: planning methods and treatment delivery and verification will be specified by each participating centre and will be the same for each group. Radiotherapy treatment should start after a minimum of 3 months and maximum of 6 months of hormonal treatment. Patients with a single prosthetic hip may be included in the trial. Beam angles for such patients should be chosen carefully to avoid having treatment fields entering through the prosthesis. Any significant image artifacts (“streaking” and/or “shadowing”) should have their densities over-ridden to that of water.

8.3 CT Planning for Radiotherapy

Patients will have planning CT scans after at least two months hormone therapy prior to commencement of radiotherapy. Prostate and planning target volumes will be defined on CT

scans which will be taken at ≤ 5 mm intervals (≤ 5mm slice thickness). The bl adder will be comfortably full, (patients to drink approximately 350 ml during the hour pre scan) and the rectum should ideally be empty of both faeces and flatus; the routine use of micro enemas (e.g. relaxit) is permissible. Positioning/immobilisation will be using approved departmental methods as specified in 7.8 for treatment delivery. Scans will be taken from the bottom of the sacro-iliac joints to the penile urethra (usually 1 cm below ischial tuberosities will be adequate).

8.4 Target Volumes and Dose Assessment Points

Volumes will be defined according to 1993 ICRU report 50 and supplement report ICRN 62: Prescribing, Recording and Reporting Photon Beam Therapy.

Two groups of patients will be defined:

1) Group 1 Low risk of seminal vesicle involvement
   a) clinical stages T1b/c or T2a/b and with PSA + ((Gleasons score -6) x10) <15

2) Group 2 Moderate or high risk of seminal vesicle involvement
   a) clinical stages T1b/c or T2a/b, and with PSA + ((Gleasons score -6) x10) >15
   b) T2c or T3a

GTV is prostate only for both Groups 1 and 2.
CTV1 is prostate and base of seminal vesicles (proximal 2cm) with 5mm margin for Group 1
CTV1 is prostate and seminal vesicles with 5mm margin for Group 2
CTV2 is prostate only for Groups 1 and 2 with 5mm margin
CTV3 is prostate only for Groups 1 and 2

The PTV 1-3 adds a 5mm margin to the relevant CTV, except that for PTV 2/3 there will be 0mm margin posteriorly or posterior inferiorly (i.e. towards the rectum).

<u>Outlining of Target Volumes</u>

In practice, PTV1 is constructed by growing a 1cm isotropic margin around the outlined prostate and all or part of the seminal vesicles. Clinical judgement should be used to ensure that inappropriately large volumes of rectum or bowel are not included in the target volume if

the seminal vesicles wrap around the rectum or small bowel or sigmoid colon are present within the target volume. For PTV2 a uniform margin of 1cm is added to the prostate alone except towards the rectum where a 5mm margin is used. Exceptionally, if there is a suspicion but not certainty - in this case the patient would not be eligible for the trial - of seminal vesicle involvement on MR scan the base of seminal vesicles can be included in PTV2. Target volumes, outlining and target isodoses are summarised in Tables 2 and 3 for low and moderate risk groups. The dose distribution to be obtained can be regarded as a core high dose region (PTV3) and two surrounding shells PTV2 - PTV3 and PTV1 - PTV2 (Figure 3). Target isodoses have been designed to achieve the following aims (Table 2 + 3):

1. Minimum (defined as to 99% of the target volume) to PTV1 will be the equivalent of 54Gy in 2Gy fractions prescribed to the isocentre and achieving target coverage by 95% isodose. This will be achieved having a 76% minimum isodose coverage. (To achieve the equivalent of 54Gy in 2Gy fractions for the conventional fractionation group, and assuming an alpha-beta ratio equal to 3, implies treating to 59.2Gy in 1.6Gy fractions. Minimum isodose coverage is therefore 100 x 59.2/74 x 0.95 = 76%).
2. Median dose to the outer shell (PTV1 - PTV2) will be the equivalent of 100% of 54Gy equivalent and equates to the 80% isodose.
3. Minimum dose to PTV2 will be the equivalent of 70Gy in 2Gy fractions prescribed to the isocentre . This will be achieved by having a 91% minimum isodose coverage. Median dose to the inner shell (PTV2 - PTV3) will be the equivalent of 70Gy and equates to the 96% isodose.
4. Median dose to PTV3 will be the equivalent to 74Gy (i.e. 100% ± 1%) with a minimum 95% isodose coverage.

Figure 2

Grade of distension	Illustration	Description
None	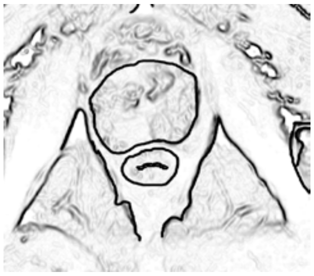	• Rectal walls opposed
Minimal	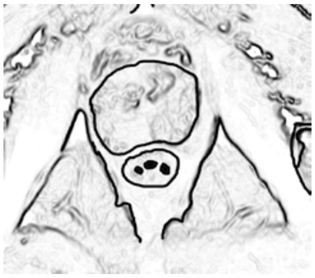	• Minimal rectal air
Small	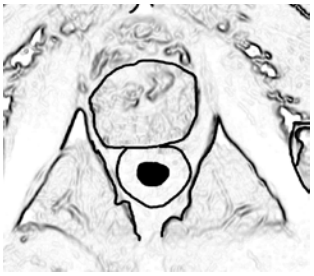	• No prostate distortion.<br>• Convex posterior prostatic border<br>• < 30% contact of rectum on posterior prostate border
Moderate	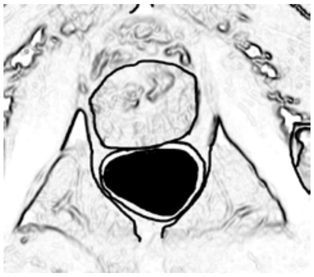	• Posterior prostatic border flattened<br>• 30-80% contact of rectum on posterior prostate border
Large	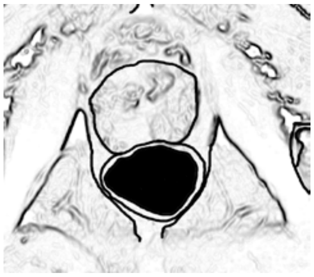	• Concave posterior prostatic border<br>• > 80% contact of rectum on posterior prostate border

Subjective grading of rectal distension

Padhani 1999 IJROBP [54]

Table 2 Summary of GTV, CTV and PTV Definitions and Dose Levels in Different Treatment Groups

Please use table 3 for outlining instructions

Low Risk Group		Moderate Risk Group		Dose				Minimum Isodose Coverage
				74 Gy Group	60 Gy Group	57 Gy Group	2 Gy equivalent+++	
GTV1	P	GTV1	P					
CTV1	P+base of SV+5mm	CTV1	P+SV+5mm	59.2	48	45.6	54 Gy	76%
PTV1	CTV +5mm	PTV1	CTV +5mm					
GTV2	P	GTV2	P					
CTV2	P+5mm	CTV2	P± base of SV++ +5mm	71	57.6	54.7	70 Gy	91%
PTV2	CTV +5mm/0mm*	PTV2	CTV + 5mm/0mm*					
GTV3	P	GTV3	P					
CTV3	P+0mm	CTV3	P+0mm	74	60	57	74 Gy	95%
PTV3	CTV +5mm/0mm+	PTV3	CTV + 5mm/0mm+					

* 0mm posteriorly toward rectum unless moderate to large rectum (see diagram) then 5mm posteriorly towards rectum to be individualised for each CT image

\+ 0mm posteriorly towards rectum all patients

++ Include base if T3B on MRI

+++ Calculated for α/β = 3.0 for 74Gy Group

Table 3 Outlining and Target Isodoses

	Low Risk	Moderate Risk	Target Isodose
PTV1	Outline: Prostate + base (proximal 2cm) of SV<br>Add: 10mm margin all directions	Outline: Prostate + SV<br>Add: 10mm margin all directions	76% minimum<br>≥ 80% median to PTV1 - PTV2
PTV2	Outline: Prostate only<br>Add:* 10mm margin except 5mm towards rectum unless moderate/large degree of rectal distension when 10mm towards rectum (only use 10mm on individual CT slices showing moderate/large rectal distension)		91% minimum<br>≥ 96% median to PV2 - PTV3
PTV3	Outline: Prostate only<br>Add: 5mm margin except 0mm towards rectum (all cases)		100%(±1%) median<br>95% minimum

* Note: PTV2 can be generated by adding a uniform margin to PTV3

Figure 3

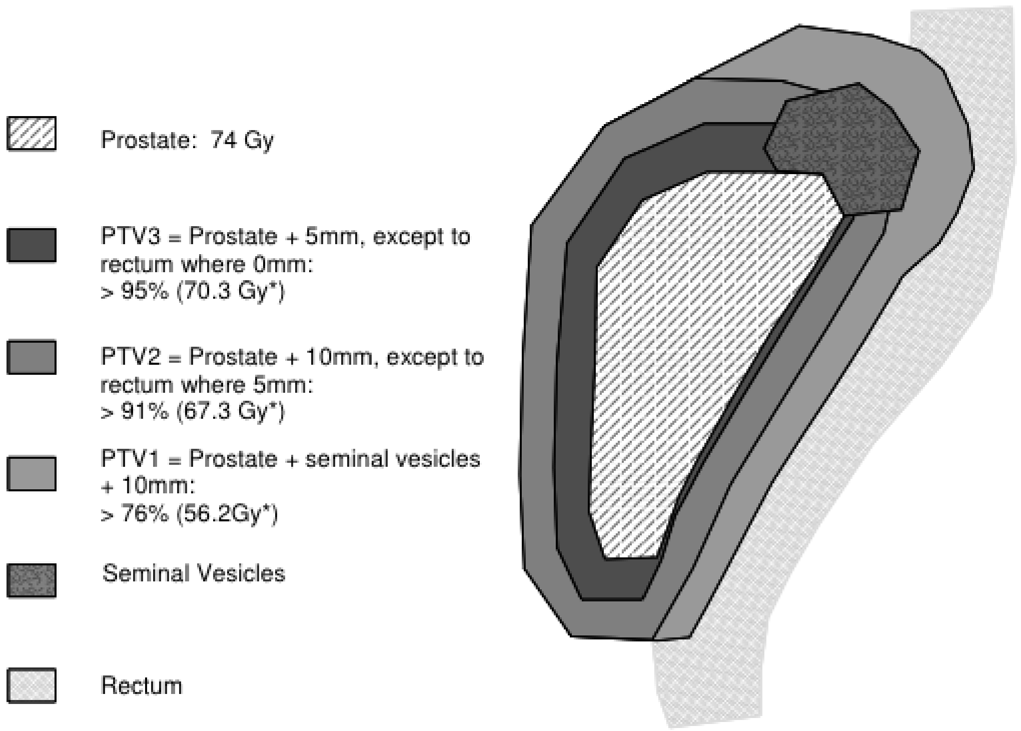

* Dose for 2Gy fractionation schedule

8.5 Normal Tissue Contouring and Dose Volume Histograms

Normal tissues outlined will include bladder, rectum, femoral heads and skin. The normal tissues will be outlined as solid organs by defining the outer wall of rectum, bladder and bowel. Bladder should be outlined from base to dome. The rectum should be outlined from the anus (usually at the level of the ischial tuberosities or 1cm below the lower margin of the PTV whichever is more inferior) to the recto-sigmoid junction. The recto-sigmoid junction can usually be identified on the CT slice where the bowel turns anteriorly and to the left. This will give a length of 10-12cm in most cases. Any additional bowel in the volume should be outlined separately.

Whenever possible dose volume histogram data evaluating dose to the GTV, PTV and organs at risk (rectum, bladder, femoral heads, bowel, urethral bulb) will be collected.

A synopsis of dose volume histogram data will be collected prospectively in all patients (see Table 4) and whenever possible dose cube data for the entire distribution will be stored for subsequent more detailed analysis.

Table 4 Normal Tissue Dose Constraints

	Dose for 2Gy/# Prescribed Dose	Dose (%)	Max Vol (% or cc)
Rectum	30	41	80%
	40	54	70%
	50	68	60%
	60	81	50%
	65	88	30%
	70	95	15%
	74	100	3%
Bladder	50	68	50%
	60	81	25%
	74	100	5%
Femoral Heads	50	68	50%

Bowel	50	68	17cc
Urethral Bulb	50<br>60	68<br>81	50%<br>10%

8.6 Radiotherapy Treatment Planning

Forward or inverse 3D planning will be undertaken using standard beam arrangements to achieve the required dose distributions in a single treatment 'phase'. These may include 3 or 4 fields (anterior/lateral/posterior) or 5 fields or more if inverse planning is utilised. The beam arrangements used in any centre must be identical for the different treatment groups and must be approved by the Radiotherapy Quality Assurance Group. Dose conformation may be achieved either using static multiphase shielding using a multileaf collimator or alternatively “step and shoot” or “moving leaf” intensity modulated dose distributions may be generated. Tissue inhomogeneity corrections will be made for the femoral heads either on a pixel by pixel or using a standardised value of bone density.

Three dimensional dose distributions should be produced. The dose distribution should be assessed for coverage of the PTV and normal tissues using appropriate transverse sagital and coronal views.

The CHHIP physics plan assessment form must be completed and assessed against the dose volume constraints (Table 4), approved by clinician and sent immediately to the ICR-CTSU, either electronically (CHHIP-icrctsu@icr.ac.uk) or by fax on 020 8722 4368.

The plan including CT images, structures, plan and dose cube, should be exported and sent on CD to the CHHIP QA Physicist.

8.7 Dose Constraints

ICRU guidelines for IMRT treatments have not yet been designed. In this study median dose to target volumes will be described and minimum and maximum doses to target volumes will be defined by isodoses which include ≥99% and ≤1% of target volumes respectively. Minimum and maximum doses (to 99% and 1% of the volume respectively) within the PTV would normally be ≥95% and ≤105% respectively. Hot spot dose outside the PTV will not exceed 105%. Dose to organs at risk outside the PTV will not exceed the median prescribed dose to PTV3. The dose to 50% of the femoral heads should not exceed 68% of the prescribed dose and the maximum dose (to 1% of the volume) should not exceed 75% of this dose. Dose constraints for rectum [10, 55-62] bladder [58, 63], femoral heads [64] urethral bulb have been derived from the literature found on data from the MRC RT01 Trial and RTOG studies [65-66] to produce low and acceptable grade 1 to 3 complications. (Table 4). For the rectum dose constraints for 50Gy to 74Gy must be attained (see below), dose constraints at 30Gy and 40Gy are for guidance only (and found on some preliminary data from MRC RT01) as are the limits for the urethral bulb.

If individual plans fail to meet the constraints, target volumes and dose distributions will be reviewed to produce a clinically acceptable option. In general, median doses to PTV3, and surrounding target shells (PTV2 minus PTV3 and PTV1 minus PTV2) should be maintained when possible with some compromise to minimum target dose coverage. Inverse planning is encouraged if the initial dose distribution was produced using a 'forward planned' IMRT solution.

Rules to be followed if dose constraints are not met.

1) Rectum.

If more than one of the rectal dose constraints (excluding 30Gy and 40Gy ‘guidance levels) is "missed" the plan should be reviewed and the following steps taken to ensure that the plan comes back within tolerance (i.e. at least 4 or 5 of the constraints are met):

(a) review the target volume, ensure that PTV3 does not overlap the rectum at all and that, in the sagittal plane, PTV2 only overlaps the rectum by 5mm. Modify the target volume if the seminal vesicles wrap around the rectum (e.g. include only proximal 1-2cm)

(b) reduce the margin in the direction of the rectum. PTV1 may be reduced from 10mm to 7mm and PTV2 from 5mm to 2mm.

(c) If the rectal dose constraints are now not adequately achieved, dose should be reduced by up to 11% (i.e. a reduction of up to 4x2Gy fractions or 2x3Gy fractions). The dose constraints for all limiting organs should then be recalculated using the original intended dose as "100%". This will mean that the various prostate target volumes are recorded as being "under dosed".

If after these three manoeuvres the patient remains out of tolerance, the patient should be withdrawn from the trial and managed according to clinical judgement.

2) If bladder constraints are out of tolerance this is likely to be due to poor bladder filling in all cases and appropriate patient instruction should solve the problem, although ideally the patient should be re-scanned with a more completely filled bladder.

3) If femoral head tolerance is exceeded a new plan should be prepared

4) Bowel tolerance level should not be exceeded. This should be a very uncommon event and only occur if a loop of bowel has become fixed in the lower pelvis.

5) Urethral bulb constraints are for guidance only – the lower the dose the more likely potency will be retained but dose to the prostate area should not be compromised.

8.8 Treatment Delivery

All fields will be treated daily on a linear accelerator ≥ of 5MV. The planned overall treatment times will be 7.5 weeks for patients receiving 74Gy and 3.8 or 4 weeks for those receiving 57 and 60Gy respectively. A maximum delay of 5 treatment days may be permitted during therapy to allow for technical difficulties. If for technical reasons a

delay for longer than this period is likely, a maximum of 5 treatments may be given with unshaped fields to the patient group receiving 74Gy and 3 such treatments in patients receiving hypofractionated schedules. If patients, particularly in the hypofractionated groups, develop significant (Grade ≥2) acute toxicities, treatment 'gaps' may be introduced to allow side effects to settle before continuing therapy.

For the hypofractionated treatment schedules overall time of treatment should be at least 27 days (for the 20 fraction schedule) and 26 days for the 19 fraction schedule. This is to avoid undue shortening of overall treatment time and, in practice, means that these patients should start treatment on a Wednesday to Friday.

Patient immobilisation and treatment accuracy will be achieved by the placing of anterior and lateral tattoos at the level of the symphysis pubis, laser alignment during treatment set-up and positioning of the legs and feet using footstocks. The Radiotherapy Quality Assurance Group will approve and monitor each centre's procedures.

8.9 Verification and Accuracy

Appropriate dose verification will be performed before treatment if IMRT inverse planning is utilised. Beam calibrations should be performed according to a specified protocol preferably that described in the IPSM report [67]. Beam calibration will be assessed using methods defined by the Quality Assurance Group.

Suitable simulator films or digitally reconstructed radiographs (DRR) will be obtained to verify the orientation and alignment of the isocentre on the linear accelerator. Port films or images will be taken so that beam alignment and configuration can be confirmed. Orthogonal anterior and lateral images will be taken to assess the position of the isocentre in relationship to simulator films or DRR.

At least 3 portal images will be taken during week 1 and subsequently at weekly intervals or as additionally appropriate if patient positioning is problematic.

Port images will be compared to simulator images or digitally reconstructed images from CT. Treatment accuracy to within 3mm is to be obtained whenever possible and positioning errors ≥5mm are unacceptable. Corrections of patient positioning and

appropriate resimulation will be employed if errors greater than this magnitude are apparent before the next radiotherapy fraction is delivered. The Radiotherapy Quality Assurance Group will approve and monitor each centre's procedures.

9. Adverse Events (AE) / Serious Adverse Events (SAE)

9.1 Definition of an Adverse Event

An 'adverse event' is any untoward medical occurrence in a patient administered a research procedure; where the events do not necessarily have a causal relationship with the procedure.

For the purpose of this trial, any detrimental change in the patient's condition subsequent to the start of the trial (i.e. randomisation) and during the follow-up period, which is not unequivocally due to progression of disease (prostate cancer), should be considered as an AE.

Whenever one or more signs and/or symptoms correspond to a disease or well-defined syndrome only the main disease/syndrome should be reported. For each sign/symptom the highest grade observed since the last visit should be reported.

9.2 Definition of Serious Adverse Events

A serious adverse event is any untoward occurrence, that:

- results in death
- is life-threatening
- requires hospitalisation or prolongation of existing hospitalisation
- results in persistent or significant disability or incapacity or
- consists of a congenital anomaly or birth defect
- additionally RTOG Grade≥4 acute or late radiation side effects i.e. related to study treatment, will be regarded as an SAE

A related adverse event is one which has been assessed by the Principal Investigator and/or Chief Investigator (or nominated representative), as resulting from any of the trial treatments or procedures.

An unexpected adverse event is any type of event not listed in the protocol as an expected occurrence.

9.3 Reporting of Adverse Events

Adverse events will be collected from the time of randomisation to the end of the follow-up period. Adverse events should be recorded in the appropriate section of the CRF.

Due acknowledgement has to be given to likely co-morbidity and co-morbid events in an elderly and ageing male population, many of whom will die from diseases unrelated to prostate cancer and its treatment.

The following are possible anticipated treatment related AEs/SAE's (i.e. expected occurrences) which are not subject to expedited reporting but all such serious events should be reported in the appropriate section of the CRF.

Bone fractures
Bowel strictures
Second Malignancies
Ureteric obstruction

9.4 Expedited reporting of SAEs

All SAEs occurring within 30 days of study procedures being administered and not listed above, are subject to expedited reporting. In addition RTOG grades ≥ 4 acute or late radiation side effects occurring within 5 years of radiotherapy treatment are subject to expedited reporting.

All SAEs must be reported within 24 hours using the CHHiP SAE form. The form must be sent by FAX to The Institute of Cancer Research – Clinical Trials and Statistics Unit (ICR-CTSU) on **020 8722 4368**. It must be completed, signed and dated by the Principal Investigator or nominated representative.

ICR-CTSU will send the SAE to the Chief Investigator (or nominated representative) for review of causality and expectedness.

9.5 Reporting related and unexpected SAEs

If an SAE is assessed as related and unexpected ICR-CTSU will report this to the main REC within 15 days from the date the ICR-CTSU became aware of the event.

9.6 SAE follow up

For each SAE, the subject must be followed-up until clinical recovery is complete and laboratory tests have returned to normal, or until the condition has stabilised. Information on final diagnosis and outcome of SAEs which may not be available at the time the SAE is initially reported should be forwarded to the ICR-CTSU in the timeframe requested.

10 Statistical Considerations

10.1 Part I: Two Centres

Design:

Standard arm	74 Gy in 37 fractions
Experimental arm A	60 Gy in 20 fractions
Experimental arm B	57 Gy in 19 fractions

Randomised on a 1:1:1 basis.

Sample size:

The sample size for this study will be determined by the rate of ≥ grade 2 symptoms reported at 2 years. Previous studies have found that the rate of ≥ grade 2 long term complications is around 15% with an upper limit of the confidence interval as high as 25%.

For each of the arms in this randomised phase II study we would like to rule out this upper limit of 25% (p_0 = 75%). We expect the rate of ≥ grade 2 complications to be

better than the previous studies, around 10% (p_1 = 90%), for the standard arm because of the IMRT radiotherapy technique used.

Using the Simon single stage design (using exact p-values [68]) with power of 87.8% and a one sided alpha of 0.045 we will recruit 50 patients per arm. In each arm if 8 or more patients develop ≥ grade 2 complications at 2 years the study arm will be rejected. This ensures that the 25% upper limit of the complication rate at 2 years is ruled out. This design allows for patient drop out during the course of the study. Each study arm will be allowed 5 patients to drop out and still have 84.1% power to detect these effects. If only 45 patients are evaluable at 2 years then 7 or more patients with ≥ grade 2 complications would result in rejection of the treatment.

There will therefore be a total of 150 patients recruited to this study.

10.2 Part II: Multicentre

The aim of the second stage of the study is to rule out a maximum toxicity in the experimental arms twice that in the standard arm.

Therefore, for each of the experimental hypofractionated arms in this randomised phase II study we would like to rule out this upper limit of 20% (p_0 = 80%). We expect the rate of ≥ grade 2 complications to be better than the previous studies, around 10% (p_1 = 90%).

Using the Simon single stage design (using exact p-values [68]) with power of 95.6% and a one sided alpha of 0.037 we will recruit 150 patients per arm. In each arm if 22 or more patients develop ≥ grade 2 complications at 2 years the study arm will be rejected. This ensures that the 20% upper limit of the complication rate at 2 years is ruled out.

This design allows for patient drop out during the course of the study. Each study arm will be allowed 15 patients to drop out and still have 95.2% power to detect these effects. If only 135 patients are evaluable at 2 years then 20 or more patients with ≥grade 2 complications would result in rejection of the treatment.

There will therefore be a total of 450 patients recruited to this part of the study.

It may also be possible to detect differences in the biochemical control rate in this part of the study. With 150 patients per arm we could detect a 16% difference in biochemical control (70% vs 54%) for control vs experimental arm respectively with 81% power using a 2 sided alpha of 0.05. This will allow us to rule out treatments that are clearly inferior to the standard arm. Depending on the alpha/beta ratios we would only expect this in the 57 Gy arm if the alpha/beta ratio for prostate cancer is >5Gy (see Table 5).

10.3 Part III: Multicentre

The third stage of this trial will be a multicentre national trial (721 patients per arm). The aim of Part III of the CHHIP trial is to demonstrate non-inferiority between the experimental arms and the control arm. The biochemical PSA control rate in the standard arm is assumed to be 70% at 5 years. Table 5 gives the number of patients required to demonstrate non-inferiority based on various minimum desirable differences to detect. These numbers are per treatment arm and have 1-sided alphas of 0.05. It can be seen that a 9% difference would be detectable in a trial with 444 men in each group (90% power) and a 6% difference with 721 men in each group (80% power). 721 men per arm would give approximately 90% power to detect differences of 7%.

Table 5

Number required per arm

Difference	80% power	90% power
**5%**	**1039**	**1439**
6%	721	**999**
**7%**	**530**	734
**8%**	**406**	**562**
**9%**	**321**	**444**
**10%**	**260**	**360**

It is also anticipated that there will be about a 6% difference between the experimental arms in ≥Grade 2 bowel toxicity. This is based on results from the MD Anderson Trial [10, 69] which showed a 14% increase (12% vs. 26%) for an 8Gy dose difference (70 vs. 78Gy) and the RMH dose escalation trial [9] which showed a 12% increase (11% vs. 23%) for a 10Gy dose difference (64 vs. 74Gy). Taken together these results suggest an approximate 1.5% increase in bowel side effects for each 1Gy increase in dose (coincidentally similar to the $\gamma(50)$ for PSA response - see below). If the standard (2Gy) group has a ≥2 RTOG toxicity level of 10% then using a 2-sided alpha of 0.05 we would require 492 patients per arm (80% power) and 659 patients per arm for 90% power to exclude ≥16% toxicity rate in either experimental arm.

Concerning the radiobiology of prostate cancer a relatively conservative estimate of the slope of the dose/control curve, $\gamma(50)=1.5$ [69, 70], appropriate for the heterogeneous population of patients which may be recruited to CHHIP, has been used to calculate expected changes in PSA control. Table 6 shows predicted changes in PSA control compared to the standard 74Gy arm for various values of the α/β ratio. There would be equivalence between 74Gy and 60Gy if $\alpha/\beta=2.5$, and 74Gy and 57Gy if $\alpha/\beta=1.5$. The difference between the two experimental arms is expected to be approximately 6%. A difference of 6% would also be seen between the standard arm and the 60Gy arm if $\alpha/\beta=1.5$ or the standard arm and the 57Gy arm if $\alpha/\beta=2.5$. For α/β outside this range of 1.5 to 2.5 the difference in PSA control would be larger and within this range, smaller. Confidence limits on the estimates of the alpha beta ratio will be calculated using Bentzen's method [45].

Table 6 Predicted % change in PSA control rate ($\gamma(50)=1.5$)

**Dose**	**α/β ratio**						
	**1.0**	1.5	**2.0**	2.5	**3.0**	**5.0**	**10.0**
**60Gy**	**+9**	+6	**+1.5**	**0**	**-3**	**-7.5**	**-12**
**57Gy**	**+3**	**0**	**-4.5**	-6	**-9**	**-13.5**	**-18**

10.4 Part IV: Multicentre

The CHHiP Trial Management Group (August 2009), the CHHiP Trial Steering Committee (August 2009) and the CHHiP Independent Data Monitoring Committee (October 2009) have recommended that recruitment should continue beyond the initial 2163 (721 men per arm) target to a revised target of 3163 (1054 men per arm). As pre-specified in Table 5 above, this would increase the power to rule out differences of 6% in PSA control from 80% to over 90% and allow a 5% difference (equivalent to a hazard ratio of 0.769) to be ruled out with over 80% power. A small allowance (1.5%) for drop out/losses to follow-up has been included. In addition to increasing the power to improve the non-inferiority margin, increasing the sample size increases the precision with which estimate event rates can be estimated and the power for pre-planned subgroup analyses (including by risk group, a stratification factor: for example, increasing the sample size from 2163 to 3163 will result in an improvement of the power in the intermediate risk subgroup from 71% to 84%).

11 Research Governance

11.1.1 Trial Administration and Logistics

The Institute of Cancer Research (ICR) is the Sponsor of this study in line with the Research Governance Framework for Health and Social Care and the principles of GCP.

The Chief Investigator is Professor David Dearnaley. ICR-CTSU has overall responsibility for facilitating and coordinating the conduct of the trial and is also responsible for collating data obtained, and undertaking and reporting interim and final analyses.

11.1.2 Participating centres responsibilities

Centres wishing to recruit to this study will be asked to provide evidence that they can deliver protocol treatment. This will include the successful completion of the CHHIP QA programme.

Responsibilities are defined in an agreement between an individual participating centre and The Institute of Cancer Research.

11.1.3 Quality Assurance of Radiotherapy Delivery

The following QA steps and exercises must be completed by new centres in order to progress:

Questionnaires, planning exercises successfully completed and process document drafted before 1st randomisation; case reviews performed before treatment starts for 1st 2/3 patients; audit visit within 6 months of commencement of patient recruitment – dose point & film measurements.

11.2 Investigator training

Prior to commencing trial recruitment, training will be provided to identify key individuals in each participating network by the Chief Investigator and Trial Management Group. Training will include discussion on the background to the study, evidence for potential benefits and drawbacks of hypofractionation and discussion on the issues of clinical equipoise. Experience developed from successfully recruiting centres and information from associated studies will be provided to participants at their initial training and subsequently on a regular basis. Participating centres will be expected to join regular (≤3 monthly) teleconferences with the Trial Management Group to discuss trial progress and identify any recruitment, treatment planning and delivery difficulties and maintain standards determined by the Quality Assurance Group.

11.3 Case Report Forms

Case Report Forms (CRFs) which are in the form of a booklet should be completed for all patients and should not be made available to third parties.

CRFs should be completed as indicated in the Investigator's brochure. CRFs are in duplicate. The completed top copy must be sent by the hospital to ICR-CTSU as soon

as they are due. The bottom copy must be retained in the booklet and held by the investigator. If information is not known it must be clearly stated.

The Trial Management Group reserves the right to amend or add to the CRFs as appropriate. Such changes do not constitute a protocol amendment, and revised or additional forms should be used by centres with immediate effect.

11.4 Protocol compliance/on site Monitoring

The CHHIP trial is being conducted in accordance with the professional and regulatory standards required for non-commercial research in the NHS under the Research Governance Framework for Health and Social Care and ICH GCP.

Participating centres may be monitored by ICR-CTSU and possibly by Health Authorities to carry out source data verification, and confirm compliance with the protocol and the protection of patients' rights as detailed in the Declaration of Helsinki adopted by the 18th World Medical assembly, Helsinki, Finland, 1964 and later revisions (last revised Edinburgh 2000)[35]. Copies of the Declaration may be obtained from ICR-CTSU on request. By participating in the CHHIP trial the Principal Investigators at each centre are confirming agreement with his/her local NHS Trust to ensure that:

- Sufficient data is recorded for all participating patients to enable accurate linkage between hospital records and CRFs;
- Source data and all trial related documentation are accurate, complete, maintained and accessible for monitoring and audit visits;
- All staff at their centre who are involved with the trial are trained appropriately;
- All original Consent Forms are dated and signed by both the patient and investigator, and are kept together in a central log together with a copy of the specific patient information sheet(s) they were given at the time of consent.
- Copies of CRFs are retained for 15 years to comply with international regulatory requirements;

- Staff will comply with the Standard Operating Procedures for CHHIP trial.

ICR-CTSU will monitor receipt of CRFs. They will also check incoming CRFs for compliance with the protocol, inconsistent and missing data.

ICR-CTSU will contact centres to discuss dates of any proposed monitoring visits. Once a date has been confirmed a list of names of patients whose notes will be monitored during the visit will be sent to the centre. This list will be sent out in advance to give sufficient time for the notes to be made available. Site monitoring will usually be conducted at participating centres at least once during the first year following entry of the first patient. It is likely that a random sample of notes will be selected for limited source document verification.

11.5 Trial Management

11.5.1 Trial Management Group

A Trial Management Group (TMG) will be set up and will include the Chief Investigator (Professor David Dearnaley), co-investigators and identified collaborators, the trial statistician and the trial co-ordinators. Principal investigators and key study personnel will be invited to join the TMG as appropriate to ensure representation from a range of centres and professional groups. Not withstanding the legal obligations of the Sponsor and Chief Investigator, the TMG has operational responsibility for the conduct of the trial.

11.5.2 Trial Steering Committee

A Trial Steering Committee (TSC) will monitor and supervise the progress of the trial. The role of the TSC is to provide overall supervision of the trial on behalf of the funding body. In particular, the TSC will concentrate on the progress of the trial, adherence to the protocol, patient safety and the consideration of new information. Day-to day management of the trial is the responsibility of the Chief Investigator and TMG.

Membership will be limited and include an independent Chairman (not involved directly in the trial other than as a member of the TSC), not less than two other independent members, the Chief Investigator and the trial statistician.

Where possible membership will include a lay/consumer representative. Trial co-ordinators and other key members of the TMG will attend meetings (as observers) as appropriate. Observers from the funding body and, if applicable, host institutions or sponsors will be invited to all meetings. The TSC will meet at least annually.

11.5.3 Data Monitoring Committee

An independent Data Monitoring and Ethics Committee (DMEC) will be established to oversee the safety and interim efficacy of the trial. This committee will be constituted according to MRC Good Clinical Practice (MRC GCP). The DMEC will meet on a regular basis as they see fit, but no less than annually. Following each meeting, the DMEC will report their findings and recommendations to the TSC and to the TMG.

11.6 End of Study

For the purposes of ethics approval, the study end date is deemed to be the date of the last data capture.

11.7 Archiving

Essential documents are documents that individually and collectively permit evaluation of the conduct of the trial and the quality of the data produced, for example CRFs, patient consent forms. These will be maintained at ICR-CTSU and at the Investigator Sites in a way that will facilitate the management of the trial, audit and inspection. They will be retained for a sufficient period (at least 15 years) for possible audit and inspection by the regulatory authority. The sponsor or trial organisers will notify the investigator sites of their responsibility for archiving essential documents. Documents will be securely stored and access will be restricted to authorised personnel. An archive log will be maintained to track archived documents

11.8 Publishing policy

All publications and presentations relating to the trial will be authorised by the TMG. A Writing Committee may be appointed. Authorship will be determined by the TMG and will include the Chief Investigator, co-investigators, and trial statisticians. Further authorship will be determined by centre accrual. All participating centres will be acknowledged in the manuscripts according to patient accrual.

12 Confidentiality and Liability

12.1 Liability/Indemnity/Insurance

This study is an investigator-led trial endorsed by the Clinical Trials Awards and Advisory Committee (CTAAC) of Cancer Research UK and the UK Medical Research Council. Indemnity for participating hospitals is provided by the usual NHS indemnity arrangements.

12.2 Patient Confidentiality

The patient’s full name, date of birth, hospital number and NHS number will be collected at randomisation to allow tracing through national records. The personal data recorded on all documents will be regarded as confidential, and to preserve each subject's anonymity, only their initials and date of birth will be recorded on subsequent Case Report Forms.

The investigator must keep a separate log of patients’ trial numbers, names, and hospital numbers. The investigator must maintain in strict confidence trial documents, which are to be held in the local hospital (e.g. patients' written consent forms). The investigator must ensure the patient's confidentiality is maintained.

ICR-CTSU will maintain the confidentiality of all subject data and will not reproduce or disclose any information by which subjects could be identified, other than reporting of serious adverse events. Representatives of the trial team will be required to have access to patient notes for quality assurance purposes but patients should be reassured

that their confidentiality will be respected at all times. In the case of special problems and/or competent authority queries, it is also necessary to have access to the complete study records, provided that patient confidentiality is protected.

13 Ethical Considerations

The trial will be performed in accordance with the recommendations guiding physicians in biomedical research involving human subjects adopted by the 18th World Medical assembly, Helsinki, Finland, 1964 and later revisions (last revised Edinburgh 2000) [73]

It is the responsibility of the Chief Investigator to obtain the required regulatory approval (Clinical Trial Authorisation) and a favourable ethical opinion (main REC approval).

It is the responsibility of the Principal Investigator at each participating Trust to obtain site-specific approval of the trial protocol and any subsequent amendments. All correspondence with the local REC should be filed by the Investigator.

It is the responsibility of the Investigator to give each patient, prior to inclusion in the trial, full and adequate verbal and written information regarding the objective and procedures of the trial and the possible risks involved. Patients must be informed about their right to withdraw from the trial and the possible risk involved. Written patient information must be given to each patient before enrolment. The written patient information is an approved patient information sheet according to national guidelines. This outlines the Quality of Life study (now closed to patient recruitment), and the collection of biological specimens. Patients will be encouraged to participate in these associated studies but if they decline, this will not exclude them from the main trial.

It is the responsibility of the Principal Investigator to obtain signed informed consent from all patients prior to inclusion in the trial.

14 Withdrawal of patients from study treatment

Patients who do not receive their allocated treatment for any reason should be treated at the discretion of their clinician. However, analyses of all outcome data will be on the

basis of intention to treat. Unless the patient requests otherwise, all CRFs, including long term follow up, should be completed, regardless of treatment actually received. A trial deviation form should be completed to record details of deviation from treatment allocation, and also for any patient who withdraws consent for further follow up. Patients are asked prior to randomisation to consent to follow up should they withdraw from the treatment allocation (see patient information sheet and consent form), and any patient unwilling to give that assurance prior to trial entry should not be randomised. Patients are; however, free to reverse that decision at any time without giving a reason.

15 Financial Matters

The trial is investigator designed and led, and has been approved by the Clinical Trials Awards and Advisory Committee (CTAAC). It is endorsed by Cancer Research UK and meets the criteria for R&D support as outlined in the Statement of Partnership on Non-Commercial R&D in the NHS in England.

Research costs (to ICR-CTSU) are being funded by Cancer Research UK. If additional financial support is received from any other source, this will be made apparent to the approving Main REC and CTAAC, but will not require a protocol amendment.

No individual per patient payment will be made to trusts or investigators, but NCRN (or regional equivalent) network resources should be made available as the trial is part of the NCRI portfolio by virtue of its approval by CTAAC.

16 Associated studies

16.1 Quality of Life (QOL)

This sub-study has now closed to recruitment.

Quality of Life is an important secondary endpoint of the trial and forms an integral part of the protocol. Patients will be informed in the patient information sheet that they will have their QOL assessed regularly while involved in the CHHiP trial.

Patients who are entered into the CHHiP Trial and are willing and able to complete the self-report QOL questionnaires are eligible to enter the QOL sub-study and will be asked to give written informed consent for their participation.

Questionnaire(s) will be given in clinic at the following times, when the patient visits for their clinic appointment.

- Initial Assessment
- Pre- Radiotherapy
- Week 10
- Post Radiotherapy (6 months)

To avoid bias, the questionnaires should be completed by the patient before they see their clinician.

The next questionnaire(s) will be posted to the patient at their home address from time to time by the Clinical Trials & Statistics Unit at the Institute of Cancer Research, and would like them completed as follows:

- at 1 year
- at 18 months
- at 2 year and then annually to 5 years.

Before the questionnaires are sent, the patient's GP or the centre will be contacted to confirm that they are fit and well to receive the questionnaire. In all cases, the Clinical Trials & Statistics Unit will send a stamped addressed envelope to the patient to return the questionnaire. If the patients agree to participate in the Quality of Life study they will need to complete a demographics form.

Patients will be asked to fill out the questionnaires themselves as completely and accurately as possible. The average time to complete the questionnaire is 10-15 minutes.

The instruments used will be the FACT-P (Prostate) and UCLA/RAND Prostate Cancer Index. However, the UCLA/RAND Prostate Cancer Index will be replaced with the

updated version. The other instruments to be used in new patients only will be the combined versions of the EPIC plus SF-12 questionnaires.

Patients already randomised to the study will receive the CHHiP questionnaire booklet version 2.1 (which includes FACT-P (Prostate), the only change will be that they will receive the updated version of the UCLA/RAND Prostate Cancer Index.

All newly randomised patients following approval of amendment no.5 will receive the CHHiP questionnaire booklet version 3.0 that will include the updated version of the UCLA/RAND Prostate Cancer Index, EPIC (- hormone section) and the SF-12 questionnaires.

16.2 Health Economics

Health economic data will be collected via patient and clinician completed health resource usage questionnaires administered at 6, 12, 24, 36, 48 and 60 months. Analysis will take place once data on the principal clinical endpoints has been analysed and published. The data will be collated in a similar way to MRC RT01 and analyses of that trial will inform evaluation.

16.3 Translational studies

Four translational projects will be progressed.

(i) Tissue microarray analysis of diagnostic biopsies and prognosis. Patients' original needle guided biopsies of the prostate will be collected and prepared for tissue microarray analysis using new methodologies developed at the Institute of Cancer Research for these samples. Assessment will include prognostic factors for the development of progressive disease and, more specifically, for response to radiotherapy - for example, hypoxia and proliferative markers.

(ii) Collection of germline DNA for contribution to UK RAPPER and EU GENEPI studies.

These projects explore the relationship between germline polymorphisms and the development of radiation related normal tissue side effects. Blood samples will be collected for inclusion in these programmes.

(iii) Collection of dose cube information for the modelling of normal tissue complication effects using conventional and, uniquely, the hypofractionated radiotherapy schedules. Dose cube data will be collected using software developed at the Institute of Cancer Research. Detailed dose volume and surface histogram analysis will be made and correlated with the development of radiation side effects and quality of life questionnaires.

(iv) To link the databases in (ii) and (iii) which will give a unique opportunity to explore detailed physical and biological parameters which may predict the development of radiation sequelae.

17. References

1. Doneux, A., et al., *The utility of digital rectal examination after radical radiotherapy for prostate cancer.* Clin Oncol (R Coll Radiol), 2005. **17**(3): p. 172-3.
2. *Consensus conference. The management of clinically localized prostate cancer.* JAMA, 1987. **258**(19): p. 2727-30.
3. *COIN Guidelines for the Management of Prostate Cancer.* Clin Oncol (R Coll Radiol), 1999. **11**(4): p. 135-72.
4. Fuks, Z. and A. Horwich, *Clinical and technical aspects of conformal therapy.* Radiotherapy and Oncology, 1993. **29**(2): p. 219-220.
5. Hanks, G.E., et al., *Dose escalation with 3D conformal treatment: five year outcomes, treatment optimization, and future directions.* Int J Radiat Oncol Biol Phys, 1998. **41**(3): p. 501-10.
6. Sandler, H.M., et al., *Dose escalation for stage C (T3) prostate cancer: minimal rectal toxicity observed using conformal therapy.* Radiotherapy and Oncology, 1992. **23**(1): p. 53-54.
7. Zelefsky, M.J., et al., *Dose escalation with three-dimensional conformal radiation therapy affects the outcome in prostate cancer.* Int J Radiat Oncol Biol Phys, 1998. **41**(3): p. 491-500.
8. Dearnaley, D.P., et al., *Comparison of radiation side-effects of conformal and conventional radiotherapy in prostate cancer: a randomised trial.* The Lancet, 1999. **353**: p. 267- 272.
9. Dearnaley, D.P., et al., *Phase III pilot study of dose escalation using conformal radiotherapy in prostate cancer: PSA control and side effects.* Br J Cancer, 2005. **92**(3): p. 488-98.
10. Pollack, A., et al., *Prostate cancer radiation dose response: results of the M. D. Anderson phase III randomized trial.* Int J Radiat Oncol Biol Phys, 2002. **53**(5): p. 1097-105.
11. *Zeitman, JAMA, 2005, 294 (10) 1233-9.*
12. Fiveash, J.B., et al., *3D conformal radiation therapy (3DCRT) for high grade prostate cancer: a multi-institutional review.* Int J Radiat Oncol Biol Phys, 2000. **47**(2): p. 335-42.
13. Kupelian, P.A., et al., *Higher than standard radiation doses (> or =72 Gy) with or without androgen deprivation in the treatment of localized prostate cancer.* Int J Radiat Oncol Biol Phys, 2000. **46**(3): p. 567-74.
14. Zelefsky, M.J., et al., *High dose radiation delivered by intensity modulated conformal radiotherapy improves the outcome of localized prostate cancer.* J Urol, 2001. **166**(3): p. 876-81.
15. Valicenti, R., et al., *Survival advantage from higher-dose radiation therapy for clinically localized prostate cancer treated on the Radiation Therapy Oncology Group trials.* J Clin Oncol, 2000. **18**(14): p. 2740-6.
16. Laverdiere, J., et al., *The efficacy and sequencing of a short course of androgen suppression on freedom from biochemical failure when administered with radiation therapy for T2-T3 prostate cancer.* J Urol, 2004. **171**(3): p. 1137-40.
17. Pilepich, M.V., et al., *Phase III radiation therapy oncology group (RTOG) trial 86-10 of androgen deprivation adjuvant to definitive radiotherapy in locally advanced carcinoma of the prostate.* Int J Radiat Oncol Biol Phys, 2001. **50**(5): p. 1243-52.
18. D'Amico, A.V., et al., *6-month androgen suppression plus radiation therapy vs radiation therapy alone for patients with clinically localized prostate cancer: a randomized controlled trial.* JAMA, 2004. **292**(7): p. 821-7.

19. Denham, J.W., et al., *Short-term androgen deprivation and radiotherapy for locally advanced prostate cancer: results from the Trans-Tasman Radiation Oncology Group 96.01 randomised controlled trial.* Lancet Oncol, 2005. **6**(11): p. 841-50.
20. Bolla, M., et al., *Long-term results with immediate androgen suppression and external irradiation in patients with locally advanced prostate cancer (an EORTC study): a phase III randomised trial.* Lancet, 2002. **360**(9327): p. 103-6.
21. Granfors, T., et al., *Combined orchiectomy and external radiotherapy versus radiotherapy alone for nonmetastatic prostate cancer with or without pelvic lymph node involvement: a prospective randomized study.* J-Urol, 1998. **159**(6): p. 2030-4 issn: 0022-5347.
22. Pilepich, M.V., et al., *Androgen suppression adjuvant to radiotherapy in carcinoma of the prostate. long-term results of phase III RTOG study 85-31.* Int J Radiat Oncol Biol Phys, 2003. **57**(2 Suppl): p. S172-3.
23. Hanks, G.E., et al., *Phase III trial of long-term adjuvant androgen deprivation after neoadjuvant hormonal cytoreduction and radiotherapy in locally advanced carcinoma of the prostate: the Radiation Therapy Oncology Group Protocol 92-02.* J Clin Oncol, 2003. **21**(21): p. 3972-8.
24. Horwitz, E.M., et al., *Subset analysis of RTOG 85-31 and 86-10 indicates an advantage for long-term vs. short-term adjuvant hormones for patients with locally advanced nonmetastatic prostate cancer treated with radiation therapy.* Int J Radiat Oncol Biol Phys, 2001. **49**(4): p. 947-56.
25. Dearnaley, D.P., *Combined modality treatment with radiotherapy and hormonal treatment in localized prostate cancer*, in *New Perspectives in Prostate Cancer*, A. Belldegrun, R.S. Kirby, and D.W.W. Newling, Editors. 2000, Isis Medical Media Ltd: Oxford. p. 169-180.
26. Forman, J.D., et al., *Neoadjuvant hormonal downsizing of localized carcinoma of the prostate: effects on the volume of normal tissue irradiation.* Cancer Invest, 1995. **13**(1): p. 8-15.
27. Zelefsky, M.J., et al., *Neoadjuvant hormonal therapy improves the therapeutic ratio in patients with bulky prostatic cancer treated with three-dimensional conformal radiation therapy.* International Journal of Radiation Oncology, Biology, Physics, 1994. **755-761**.
28. D'Souza, W.D. and H.D. Thames, *Is the alpha/beta ratio for prostate cancer low?* Int J Radiat Oncol Biol Phys, 2001. **51**(1): p. 1-3.
29. Fowler, J., R. Chappell, and M. Ritter, *Is alpha/beta for prostate tumors really low?* Int J Radiat Oncol Biol Phys, 2001. **50**(4): p. 1021-31.
30. Fowler, J.F., R.J. Chappell, and M.A. Ritter, *The prospects for new treatments for prostate cancer.* Int J Radiat Oncol Biol Phys, 2002. **52**(1): p. 3-5.
31. King, C.R. and J.F. Fowler, *A simple analytic derivation suggests that prostate cancer alpha/beta ratio is low.* Int J Radiat Oncol Biol Phys, 2001. **51**(1): p. 213-4.
32. Brenner, D.J., *Toward optimal external-beam fractionation for prostate cancer.* Int J Radiat Oncol Biol Phys, 2000. **48**(2): p. 315-6.
33. Duchesne, G.M. and L.J. Peters, *What is the alpha/beta ratio for prostate cancer? Rationale for hypofractionated high-dose-rate brachytherapy.* Int J Radiat Oncol Biol Phys, 1999. **44**(4): p. 747-8.
34. Brenner, D.J., et al., *Direct evidence that prostate tumors show high sensitivity to fractionation (low alpha/beta ratio), similar to late-responding normal tissue.* Int J Radiat Oncol Biol Phys, 2002. **52**(1): p. 6-13.
35. Nahum, A.E., et al., *Incorporating clinical measurements of hypoxia into tumor local control modeling of prostate cancer: implications for the alpha/beta ratio.* Int J Radiat Oncol Biol Phys, 2003. **57**(2): p. 391-401.

36. Brenner, D.J., *Hypofractionation for prostate cancer radiotherapy--what are the issues?* Int J Radiat Oncol Biol Phys, 2003. **57**(4): p. 912-4.
37. Thames, H.D., Jr., et al., *Changes in early and late radiation responses with altered dose fractionation: implications for dose-survival relationships.* Int J Radiat Oncol Biol Phys, 1982. **8**(2): p. 219-26.
38. Brenner, D.J. and E.J. Hall, *Fractionation and protraction for radiotherapy of prostate carcinoma.* Int J Radiat Oncol Biol Phys, 1999. **43**(5): p. 1095-101.
39. Duncan, W., et al., *Carcinoma of the prostate: results of radical radiotherapy (1970-1985).* International Journal of Radiation Oncology, Biology, Physics, 1993. **26**(2): p. 203-210.
40. Read, G. and R.C.S. Pointon, *Retrospective study of radiotherapy in early carcinoma of the prostate.* British Journal of Urology, 1989. **63**(2): p. 191-195.
41. Collins, C.D., R.W. Lloyd-Davies, and A.V. Swan, *Radical external beam radiotherapy for localised carcinoma of the prostate using a hypofractionation technique.* Clinical Oncology, 1991. **3**(3): p. 127-132.
42. Lukka, H., et al., *A randomized trial comparing two fractionation schedules for patients with localized prostate cancer.* Int J Radiat Oncol Biol Phys, 2003. **57**(2 Suppl): p. S126.
43. Yeoh, E.E., et al., *Evidence for efficacy without increased toxicity of hypofractionated radiotherapy for prostate carcinoma: early results of a Phase III randomized trial.* Int J Radiat Oncol Biol Phys, 2003. **55**(4): p. 943-55.
44. Livsey, J.E., et al., *Hypofractionated conformal radiotherapy in carcinoma of the prostate: five-year outcome analysis.* Int J Radiat Oncol Biol Phys, 2003. **57**(5): p. 1254-9.
45. Bentzen, S.M. and M.A. Ritter, *The alpha/beta ratio for prostate cancer: what is it, really?* Radiother Oncol, 2005. **76**(1): p. 1-3.
46. Kupelian, P.A., et al., *Preliminary observations on biochemical relapse-free survival rates after short-course intensity-modulated radiotherapy (70 Gy at 2.5 Gy/fraction) for localized prostate cancer.* Int J Radiat Oncol Biol Phys, 2002. **53**(4): p. 904-12.
47. Mott, J.H., J.E. Livsey, and J.P. Logue, *Development of a simultaneous boost IMRT class solution for a hypofractionated prostate cancer protocol.* Br J Radiol, 2004. **77**(917): p. 377-86.
48. Akimoto, T., et al., *Rectal bleeding after hypofractionated radiotherapy for prostate cancer: correlation between clinical and dosimetric parameters and the incidence of grade 2 or worse rectal bleeding.* Int J Radiat Oncol Biol Phys, 2004. **60**(4): p. 1033-9.
49. Thames, H.D., et al., *Time-dose factors in radiotherapy: a review of the human data.* Radiother Oncol, 1990. **19**(3): p. 219-35.
50. Brenner, D.J., *Fractionation and late rectal toxicity.* Int J Radiat Oncol Biol Phys, 2004. **60**(4): p. 1013-5.
51. Sydes, M.R., et al., *Implementing the UK Medical Research Council (MRC) RT01 trial (ISRCTN 47772397): methods and practicalities of a randomised controlled trial of conformal radiotherapy in men with localised prostate cancer.* Radiother Oncol, 2004. 72(2): p. 199-211.
52. Diaz, A., et al., *Indications for and the significance of seminal vesicle irradiation during 3D conformal radiotherapy for localized prostate cancer.* International Journal of Radiation Oncology, Biology, Physics, 1994. **30**(2): p. 323-329.
53. Parker, C.C. and D.P. Dearnaley, *The management of PSA failure after radical radiotherapy for localised prostate cancer.* Radiotherapy and Oncology, 1998. **49**(2): p. 103-110.
54. Padhani, A.R., et al., *Evaluating the effect of rectal distension and rectal movement on prostate gland position using cine MRI.* Int. J. Radiation Biol. Phys., 1999. **44**(3): p. 525-533.

55. Fiorino, C., et al., *Relationships between DVHs and late rectal bleeding after radiotherapy for prostate cancer: analysis of a large group of patients pooled from three institutions.* Radiother Oncol, 2002. **64**(1): p. 1-12.
56. Fiorino, C., et al., *Rectal dose-volume constraints in high-dose radiotherapy of localized prostate cancer.* Int J Radiat Oncol Biol Phys, 2003. **57**(4): p. 953-62.
57. Lu, Y., et al., *A method of analyzing rectal surface area irradiated and rectal complications in prostate conformal radiotherapy.* Int J Radiat Oncol Biol Phys, 1995. **33**(5): p. 1121-5.
58. Storey, M.R., et al., *Complications from radiotherapy dose escalation in prostate cancer: preliminary results of a randomized trial.* Int J Radiat Oncol Biol Phys, 2000. **48**(3): p. 635-42.
59. Benk, V.A., et al., *Late rectal bleeding following combined x-ray and proton high dose irradiation for patients with stage T3-T4 prostate carcinoma.* International Journal of Radiation Oncology, Biology, Physics, 1993. **26**(3): p. 551-557.
60. Boersma, L.J., et al., *Estimation of the incidence of late bladder and rectum complications after high-dose (70-78 GY) conformal radiotherapy for prostate cancer, using dose-volume histograms.* Int-J-Radiat-Oncol-Biol-Phys, 1998. **41**(1): p. 83-92 issn: 0360-3016.
61. Jackson, A., et al., *Late rectal bleeding after conformal radiotherapy of prostate cancer. II. Volume effects and dose-volume histograms.* Int J Radiat Oncol Biol Phys, 2001. **49**(3): p. 685-98.
62. Wachter, S., et al., *Rectal sequelae after conformal radiotherapy of prostate cancer: dose-volume histograms as predictive factors.* Radiother Oncol, 2001. **59**(1): p. 65-70.
63. Marks, L.B., et al., *The response of the urinary bladder, urethra, and ureter to radiation and chemotherapy.* Int J Radiat Oncol Biol Phys, 1995. **31**(5): p. 1257-80.
64. Emami, B., et al., *Tolerance of normal tissue to therapeutic irradiation.* International Journal of Radiation Oncology, Biology, Physics, 1991. **21**(1): p. 109-22.
65. *Mangar, S et al., Evaluating the relationship between erectile dysfunction and dose received by the penile bulb. Radiother Oncology, 2006 in Press*
66. *Roach et al., Penile bulb dose and impotence after three-dimensional conformal radiotherapy for prostate cancer on RTOG 9407. Int J Radiat Oncol Biol Phys 2004;60:1351-6.*
67. *IPSM Report, Physics Med Biol 35 (10) ppl 355-60*. 1990,.
68. A'Hern, R.P., *Sample size tables for exact single-stage phase II designs.* Stat Med, 2001. **20**(6): p. 859-66.
69. Cheung, R., et al., *Dose-response for biochemical control among high-risk prostate cancer patients after external beam radiotherapy.* Int J Radiat Oncol Biol Phys, 2003. **56**(5): p. 1234-40.
70. Levegrun, S., et al., *Risk group dependence of dose-response for biopsy outcome after three-dimensional conformal radiation therapy of prostate cancer.* Radiother Oncol, 2002. **63**(1): p. 11-26.
71. Pollack, A., et al., *Preliminary results of a randomized radiotherapy dose-escalation study comparing 70 Gy with 78 Gy for prostate cancer.* J Clin Oncol, 2000. **18**(23): p. 3904-11.
72. Peeters, S. T., W. D. Heemsbergen, et al. (2006). *"Dose-response in radiotherapy for localized prostate cancer: results of the Dutch multicenter randomized phase III trial comparing 68 Gy of radiotherapy with 78 Gy."* J Clin Oncol **24**(13): 1990-6.
73. http://www.wma.net/e/ethicsunit/helsinki.htm
74. http://www.nccn.org (10th November 2006)

75. Roach M, Hanks G, Thames H Jr et al. *Defining biochemical failure following radiotherapy with or without hormonal therapy in men with clinically localized prostate cancer: recommendations of the RTOG-ASTRO Phoenix Concensus Conference.* Int J Radiat Oncol Biol Phys 2006; **66**: 965-74

APPENDIX 1 – Image Guided Radiotherapy (IGRT) Sub-study

SUB-STUDY SUMMARY

Development of complex, new technologies has lead to the introduction of daily on-line image guided radiotherapy (IGRT) as a form of treatment verification in some centres in the UK. Image guided techniques can track a mobile organ like the prostate throughout a seven week course of radiotherapy treatment. With better localisation, IGRT may improve tumour control probability and if the margins around the prostate are reduced this may reduce normal tissue toxicity. IGRT requires more resources (equipment, treatment time, skills) and the potential benefits have not been tested in a randomised trial. This sub-study will explore the feasibility of a phase III randomised trial of IGRT; i.e. how many study centres will be able to perform IMRT with IGRT, what proportion of patients will accept randomisation and the accrual rate for the sub-study. Acute and late toxicity rates will be estimated.

Background

Rationale for IGRT sub-study

The success of radical prostate radiotherapy depends on an accurate delivery of high dose radiotherapy to a defined tissue volume with a high degree of positional accuracy. Standard verification protocols are based on retrospective assessments of bony landmarks imaged with portal megavoltage imaging systems and require generous margins around the target volume to ensure sufficient cumulative delivered dose. Two randomised studies [3, 4] have shown that a large rectal volume at planning is linked to reduced tumour control, implying, that larger rectums produce more prostate shifts and a reduced target volume coverage. In a large cohort study patients with large rectums were given appropriate measures to reduce rectal volume (laxatives, suppositories and diet) and rescanned. These patients had similar prostate motion during treatment compared to patients with small rectums throughout [5].

Throughout a course of radiotherapy, the prostate position changes mainly in the anterior/posterior and superior/inferior direction in relation to bony markers due to changes in rectal distension; this mobility is not predictable [6-8]. With a standard bony-

landmark-based imaging protocol, 10mm margins around the target volume are required to ensure sufficient coverage of the tumour for the majority of treatments [4, 10].

Daily IGRT will increase the accuracy of dose delivery and may be expected to improve tumour control without any other changes in treatment technique [11-31], but IGRT requires either an additional invasive procedure (insertion of fiducial markers into the prostate) or purchase of expensive capital equipment (helical tomotherapy, CT on rails). IGRT may allow a reduction in applied margins without losing target coverage, which should translate into reduced normal tissue toxicity [14]. Reduced toxicity may allow further safe dose escalation and improved tumour control [15]. Our earlier dose-escalation trial, using conformal methods, clearly showed that a margin reduction of 1.5cm to 1.0cm was associated with a statistically significant reduction in both acute and late side effects [1].

IGRT technique

There has been a large number of single centre studies published developing and exploring the technical details of different IGRT approaches. Fiducial markers are most commonly used [16-18] and seem to be fairly stable once in position [19, 20]. They are easily visible on most megavoltage or kilo-voltage imaging systems; the matching accuracy is better than bone matching [18, 19], but a separate procedure is required to insert them [22]. Infra-fraction motion and outlining uncertainties still will require a small margin [21-25]. Other options are the localisation of the prostate with tomotherapy, cone beam CT scans, CT on rails or ultrasound [26-31]. These techniques require different (often very expensive) equipment; the experience with tomotherapy, CT on rails and cone beam CT is considered to be reproducible and accurate compared with fiducial markers [29-31]. All modalities are non-invasive, but increase radiation doses to the pelvis for imaging purposes. It is possible to model the advantages of an IGRT approach [9,10]. However, no evaluation of clinical benefits in form of reduced toxicity or improved tumour control has been performed. Within the UK, clinicians with access to and experience of cone beam CT have not felt that the image quality is presently

sufficient in some units to consider this suitable for daily online IGRT. Clinicians with access to and experience of tomotherapy and CT on rails have felt that the image quality is presently sufficient to consider this suitable for daily online IGRT.

GTV to PTV margins required for daily IMRT

Pilot studies of the accuracy of IGRT using fiducial markers have been undertaken at two of the initial CHHiP centres. The Royal Marsden Hospital (RMH) studied 30 patients in detail [21] and found that required treatment margins using fiducial markers and daily on-line imaging were (with associated systematic (Σ) and random errors (σ)): in the left/right (LR) direction: 1.3mm (Σ=0.3mm, σ=0.8mm); in the anterior posterior (AP) direction: 1.2mm (Σ=0.3mm, σ=0.7mm); and in the superior inferior (SI) direction: 1.2mm (Σ=0.3mm, σ=0.7mm). When intra-fraction motion was taken into account the required margins were estimated at LR: 3.3mm (Σ=0.9mm, σ=1.2mm); AP: 3.6mm (Σ=0.9mm, σ=2.7mm); SI: 3.4mm (Σ=0.9mm, σ=1.6mm).

At the Clatterbridge Centre for Oncology (CCO) [33], a randomised study assessing prostate mobility during radiotherapy has been carried out to compare the accuracy of on-line versus off-line correction strategies using implanted marker seeds; this study was completed in September 2009. 150 prostate patients had fiducial gold markers implanted prior to planning. All received radical radiotherapy and were imaged either daily with corrections prior to treatment or according to the standard imaging protocol. Weekly cone beam CT scans were performed to compare fiducial markers with cone beam localization. Systematic (Σ) and random errors (σ) were calculated and coverage of the planning target computed for both groups. Resource requirements were assessed for the daily imaging technique by analyzing the daily in-room timings performed on 10 patients. In a preliminary analysis of the first 60 patients of the daily imaging group, daily imaging was assessed as being potentially beneficial for the majority of patients. With the standard imaging protocol the following shifts would have occurred: LR: mean = 0.1mm (Σ=2.6mm, σ=1.8mm), AP: mean = 2.2mm (Σ=3.2mm, σ=2.5mm) and SI): mean = -1.0mm (Σ=1.2mm, σ=2.2mm). Taking intra-fraction motion into account, on-line corrections reduced the mean error LR to -0.1± 0.7mm, AP to 0.6±1.9mm and SI to 0.2 ± 3.5mm. Margins required for the standard correction

protocol were RL = 4mm, SI = 5mm and AP=7mm; for daily correction protocol the required margin were smaller: 1mm, 2mm and 4mm. Timings showed that the on-line imaging can be performed with a moderate increase in the standard treatment slot [33].
Both these studies clearly demonstrate the potential to very substantially reduce the treatment margins using IGRT. The results from the two centres are broadly similar but indicate that reduction of margins below 2-3mm would be inappropriate due to intra-fraction movement.

IGRT as sub-study for CHHiP

During 2009, five CHHiP recruitment centres became able to offer IGRT to increasing numbers of patients. Rather than introducing this technology in a piecemeal fashion this sub-study takes the opportunity to embed a more formal study of IGRT within the existing CHHiP protocol. The CHHiP trial offers one of the very few opportunities to more formally assess IGRT in a subgroup of patients.

Present access to prostate cancer IGRT in the UK is limited: just over half of 50 UK centres have IGRT equipment although over 80% are expected to have some by 2010. The majority of centres with IGRT capabilities are using it for prostate cancer and about half of these using some sort of daily, online IGRT [Mayles, personal communication]. Several centres are aiming to introduce fiducial markers and tomotherapy is being introduced within the NHS to Middlesbrough and Newcastle (CT on rails) in addition to Addenbrookes. A study performed now would help to define a UK-wide prostate IGRT protocol and act as a conduit to increased uptake of this technology (similar to the role CHHiP has already had in introducing IMRT across the country – see CHHiP publications list, Appendix C).

Objectives for the IGRT sub-study

- To assess the acute and late toxicity associated with IGRT
- To determine feasibility of a phase III randomised trial of IGRT in the treatment of localised prostate cancer.

Sub-study design

IGRT Sub-study: The aim is to assess the feasibility of phase III randomised trial of IGRT in prostate cancer and to collect randomised phase II data on toxicity in order to inform the design of a subsequent phase III trial. The IGRT randomisation would be available to all patients entered into CHHiP at centres participating in the IGRT sub-study. The IGRT randomisation will be stratified by dose/fractionation schedule i.e. by allocated CHHiP treatment group.

At randomisation into the main CHHiP trial, patients are also (optionally) randomised to:

(A) no IGRT
standard CHHiP planning margins

(B) IGRT
standard CHHiP planning margin

(C) IGRT
reduced margins

Patients in (A) will be planned and treated as in the main protocol.

Patients in (B) and (C) will be treated using online daily IGRT with either 2-4 implanted fiducial markers, helical tomotherapy or CT on rails.

Inclusion of a no IGRT arm (A) allows a direct assessment of the use of IGRT (B vs. A); the clinical value of IGRT is likely to be through the reduction of planning margins leading to less normal tissue effects (arm C). Centres with no experience of IGRT may choose to randomise between arms (A) and (B) (keeping planning margins the same for all patients) whilst they gain confidence in IGRT delivery. Centres that are currently treating all CHHiP patients with IGRT would be permitted to drop arm (A). The three way randomisation will appeal to centres that have initial experience using IGRT but are not offering this routinely to all patients. To maximize the statistical efficiency of the trial, centres will be encouraged to adopt the 3-way randomisation. The decision to drop either arm (C) or arm (A) must be notified to ICR-CTSU prior to randomisation of patients into the sub-study.

IGRT sub-study – additional exclusion criteria

In addition to the inclusion/exclusion criteria for the main trial patients with any of the following are ineligible for the sub-study.

- Increased risk of toxicity from implanted fiducial markers e.g. severe toxicity (infection/bleeding) from diagnostic TRUS biopsy, anticoagulated patients
- Single hip prosthesis or fixation which would interfere with positional imaging

Primary and Secondary Sub-study Endpoints

Primary:

- Late radiation induced side effects.

Secondary*:*

- Feasibility of a phase III randomised trial i.e. no. of centres with IGRT capability, proportion of patients accepting randomisation and accrual rate.

Informed consent, Randomisation and treatment allocation

The investigator is responsible for obtaining each patient's signed informed consent prior to the administration of the IGRT sub-study. After registration into the main CHHiP trial, the patients should be randomised for both the main study and the sub-study as close to the start of radiotherapy as possible. Patients are randomised into the main study and the sub-study by a single telephone call to the ICR-CTSU. The patient's registration number will be required at the time of randomisation.

**Patients are randomised by telephone through the ICR-CTSU**
**Tel**: 020 8643 7150 (09.00 – 17.00 Monday to Friday)

The caller will be given the patient's unique trial identification number (Trial ID) and treatment allocation. Treatment allocation will be 1:1:1 and will use computer generated random permuted blocks. The sub-study randomisation will be stratified by treating centre and radiotherapy dose group.

A letter confirming randomisation will be sent to the centre to confirm treatment allocation.

Investigations, Assessment Procedures and Data Collection

The initial assessment and the assessment during treatment and follow up are identical to the main study. Patients in the sub-study require acute toxicity assessment. Data will be collected as part of the main CHHiP trial. The sub-study would be adopted by a number of CHHiP centres immediately. Over the next 6-12 months it is likely that a number of other centres would have the technological capability and resources to contribute.

Radiotherapy planning and treatment

Rectal preparation (all arms)

The staging MRI/ CT scan of all patients in the study is reviewed prior to planning. If the rectum is large and/or the patient is constipated, the patient receives regular laxatives and a diet sheet to start a week prior to the planning scan. The choice of drugs and diet sheet is the centre's decision.

If the rectum is larger than 4 cm in anterior/posterior direction at any level of the prostate give laxatives/mini-enema and rescan. It is expected to rescan about 20% of patients [5]. If the rectum remains large despite the above measures, it is recommended to plan on the second scan.

Implanted fiducial marker protocol (arm B or C)

Fiducial markers inserted using trans-rectal ultrasound guidance and antibiotic cover approximately 2 weeks prior to the radiotherapy planning scan. Image acquisition, target volume definition and planning are performed as in main protocol. No adjustments of margins posterior are made for patients with large (> 4cm rectal diameter). During treatment, patients are aligned using the standard laser alignment technique according to the individual patient's treatment plan. A pre-treatment image of the anterior and lateral direction is taken; any observed set-up error greater than or

equal to 2mm in any direction is corrected prior to treatment. No post-correction imaging is required. Each centre will keep a record of corrections applied for each trial patient.

Tomotherapy or CT on rails protocol (arm B or C)

No markers are inserted. Image acquisition, target volume delineation and planning as in main protocol. No adjustments of margins posterior are made for patients with large (> 4cm rectal diameter). During treatment, patients are aligned using the standard laser alignment technique according to the individual patient's treatment plan. A pre-treatment CT is taken; any observed set-up error greater than or equal to 2mm is corrected prior to treatment. No post-correction imaging is required. Each centre will keep a record of corrections applied for each trial patient.

Reduced planning margins (arm C)

A literature review [4,15-21,33], and work from RMH, CCO have been used to calculate margins to incorporate residual set up errors and infra fraction motion, using the van Herk formula [4]: RL 1.4 to 3.3 mm; SI 2.3 to 3.4 mm and AP 3.6 to 3.9 mm [McNair, Syndikus personal communication]. The margins include possible microscopic spread of tumour outside the prostate capsule (CTV), which is estimated to be small in good risk prostate cancer.

Modelling works suggests that this level of margin reduction can lead to a 30% to 40% reduction in PTV volume and 30 to 50% reduction in volume of rectum irradiated to critical dose levels (dependent on planning technique and dose level assessed), which we believe is sufficient to expect a clinically significant reduction in toxicity.

Direction	Arm A, B (Standard margin)		Arm C (Reduced margin)	
	Anterior, right, left, superior, inferior	Posterior	Anterior, right, left, superior, inferior	Posterior
GTV1 to PTV1	10 mm	10 mm	6 mm	6 mm
GTV2 to PTV2	10 mm	5 mm	6 mm	3 mm
GTV3 to PTV3	5 mm	0 mm	3 mm	0 mm

Quality Assurance (QA)

The QA program for the IGRT sub-study will complement pre-trial QA for the main CHHiP trial; it will comprise a process document to collect information on the centre's IGRT technique and processes. Additionally, measurements to be performed by each centre to assess the accuracy of the imaging and corrections applied. The imaging results for each patient's treatment in each arm of the IGRT sub-study will be documented following guidelines defined by the American Society of Radiation Oncology (ASTRO) [32].

Sample Size and Statistical Considerations

Each sub-study treatment group is powered independently (using the Simon single stage design with exact p-values, as for CHHiP parts 1 & 2) though data would contribute to a comparative analysis in any future phase III or expanded phase II study.

It is anticipated that the control group (A: No IGRT) will have a 2-year toxicity-free rate of 80% and that we are interested in detecting a 10% to 15% difference in the experimental IGRT groups.

If 91 patients are entered into each group, the toxicity-free rate will be considered high enough to warrant undertaking a phase III trial if 79 or more patients remain toxicity-free in the experimental group. If the true toxicity-free rate is 80% there is a 3.4% chance that 79 or more patients will remain toxicity-free (alpha error rate). If the true toxicity-free rate in this group is 90% there is an 80.3% chance (power) that 79 or more will remain toxicity-free.

If 50 patients are entered into each group, the toxicity-free rate will be considered high enough to warrant undertaking a phase III trial if 45 or more patients remain toxicity-free in the experimental group. If the true toxicity-free rate is 80% there is a 1.8% chance that 45 or more patients will remain toxicity-free (alpha error rate). If the true toxicity-free rate in this group is 95% there is an 89.6% chance (power) that 45 or more will remain toxicity-free.

Given that monthly accrual into CHHiP is increasing, we propose to enter a minimum of 150 patients (50 per group) with a target sample size of 273 patients (91 per group) to the IGRT sub-study.

The intention is to compare groups A vs. B and B vs. C with regard to the two year grade ≥2 RTOG toxicity (bowel or bladder) according to the rules described above (IGRT-sub-study). Absolute numbers and proportions of patients toxicity-free at two years together with exact binomial confidence intervals will be presented. The IDMC will review a first interim report to assess recruitment and compliance to IGRT technique and margin requirements 6 months after the first randomisation or when half the required number of patients will be accrued which ever occurs first. In a second interim analysis when the median follow-up will reach one year, the IDMC will look at the first two years toxicity data. The final IGRT analysis will be conducted with recommendation from the IDMC when the minimum follow-up of the sub-study is two years.

Appendix 1 – Sub-study References

1. *Dearnaley DP, Hall E, Lawrence D, Huddart RA, Eeles R, Nutting CM, et al. Phase III pilot study of dose escalation using conformal radiotherapy in prostate cancer: PSA control and side effects. Br J Cancer 2005; 92(3), 488-98.*
2. *DeCrevoisieur R, Tucker S, Dong L, et al. Increased risk of biochemical and local failure in patients with distended rectum on the CT planning for prostate radiotherapy. Int J Radiat Oncol Biol Phys 2005; 62, 965-973.*
3. *Heemsbergen WD, Hoogeman MS, Witte MG, et al. Increased risk of biochemical and clinical failure for prostate patients with a large rectum at radiotherapy planning: results from the Dutch trial of 68Gy versus 78Gy. Int J Radiat Oncol Biol Phys 2007; 67, 1418-1423.*
4. *Van Herk M, Remeijer P, Rasch C, Lebesque JV. The probability of correct target dosage: dose-population histograms for deriving treatment margins in radiotherapy. Int J Radiat Oncol Biol Phys 2000; 47, 1121-35.*

5. *Stillie AL, Kron T, Fox C et al. Rectal filling at planning does not predict stability of the prostate gland during a course of radical radiotherapy if patients with a large rectum are re-imaged. Clin Oncol 2009; 21, 760-767.*
6. *Aubry JF, Beaulieu L, Girouard L.-M., Aubin S, Tremblay D, Laverdière J, Vigneault E. Measurements of intrafraction motion and interfraction and intrafraction rotation of prostate by three-dimensional analysis of daily portal imaging with radiopaque markers. Int J Radiat Oncol Biol Phys 2004; 60(1), 30-39.*
7. *Jergin Chen, R. Jeffrey Lee, Diana Handrahan, William T. Sause. Intensity-Modulated Radiotherapy Using Implanted Fiducial Markers With Daily Portal Imaging: Assessment of Prostate Organ Motion . Int J Radiat Oncol Biol Phys 2007; 68(3), 912-919.*
8. *Alexis N.T.J. Kotte, Pieter Hofman, Jan J.W. Lagendijk, Marco van Vulpen, Uulke A. van der Heide. Intrafraction Motion of the Prostate During External-Beam Radiation Therapy: Analysis of 427 Patients with Implanted Fiducial Marker. Int J Radiat Oncol Biol Phys 2007; 69(2), 419-425.*
9. *Ghilezan M, Yan D, Liang J, et al. Online image-guided intensity-modulated radiotherapy for prostate cancer: How much improvement can we expect? A theoretical assessment of clinical benefits and potential dose escalation by improving precision and accuracy of radiation delivery. Int J Radiat Oncol Biol Phys 2004; 60:, 1602-1610.*
10. *Kupelian PA, Langen, KM, Zeidan, OA et al. Daily variations in delivered doses in patients treated with radiotherapy for localized prostate. Int J Radia Oncol Biol Phys. 2006; 66, 876-882.*
11. *Van den Heuval F, Fugazzi J, 55.Soete G, Verellen D, Storme G. Image guided radiotherapy for prostate cancer. Bulletin du Cancer 2008; 95, 374-380.*
12. *Seppi E, Forman JD. Clinical application of a repositioning scheme, using gold markers and electronic portal imaging. Radiother Oncol 2006; 79, 94-100.*
13. *Wu Q, Ivaldi G, Liang J et al. Geometric and dosimetric evaluations of an online image-guided strategy for 3DCRT of prostate cancer. Int J Radiat Oncol Biol Phys 2006; 64, 1596-1609.*

14 *Schulz D, Liang J, Yan D, Zhang T. Comparison of various online IGRT strategies: The benefits of online treatment plan re-optimization**.** Radiother Oncol 2009; 90, 367-376.*

15 *Craig T, Satkusagingham J, Chan K, Brock K, Moseley J, Chung P, Bayley A, Crook J, Jaffray D, Menard C. Advanced Image Guidance Allows Margin Reduction in Radiation Therapy of Prostate Cancer. Int J Radiat Oncol Biol Phys 2008; 72(1), S551.*

16 *Van der Heide, Kotte NTJ, Dehnad H, Hofman P, Lagenijk JJW, van Vulpen M. Analysis of fiducial marker-based position verification in the external beam radiotherapy of patients with prostate cancer. Radiother Oncol 2007; 82, 38-45.*

17 *Chung PW, Haycocks T, Brown T, et al. On-line aSi portal imaging of implanted fiducial markers during conformal radiotherapy of the prostate carcinoma. Int J Radiat Oncol Biol Phys 2004; 60, 329-334.*

18. *Van der Vight L P, van Lin EMJT, Spiiters-Post I, Visser AG, Louwe RJW. Off- line setup corrections only marginally reduce the number of on-line corrections for prostate radiotherapy using implanted gold markers. Radiother Oncol 2009; 90, 359-366.*
19. *Kupelian PA, Willoughby TR, Meeks SL, et al. Intraprostatic fiducials for localization of the prostate gland: monitoring inter-marker distances during radiation therapy to test for marker stability. Int J Radiat Oncol Biol Phys 2005; 62, 1291-1296.*
20. *Pouliot J, Aubin M, Langen KM, et al. (Non)-migration of radio-opaque markers used for on-line localization of the prostate with an electronic portal imaging device. Int J Radiat Oncol Biol Phys 2003; 56, 862-866.*

21. *McNair HA, Hansen VN, Parker CR, et al. Comparison of the use of bony anatomy and internal markers for offline verification and an evaluation of the potential benefit of online and offline verification protocols for prostate radiotherapy. Int J Radiat Oncol Biol Phys 2008; 71, 41-50.*
22. *Shinohara K, Roach M 3rd. Technique for implantation of fiducial markers in the prostate. Urology. 2008; 71(2):196-200.*
23. *Huang E, Dong L, Chandra A et al. Intrafraction prostate motion during IMRT for prostate cancer, Int J Radiat Oncol Biol Phys 2002; 53, 261–268.*
24. *Litzenberg, D.W., Balter, J.M., Hadley, S.W., Sandler, H.M., Willoughby, T.R., Kupelian, P.A., Levine, L. Influence of intrafraction motion on margins for prostate radiotherapy. Int J Radiat Oncol Biol Phys 2006; 65(2), 548-553.*
25. *Cheung P, Sixel K, Morton, G et al. Individualized planning target volumes for intrafraction motion during hypofractionated intensity-modulated radiotherapy boost for prostate cancer. Int. J. Radia. Oncol. Bio. Phys. 2005; 62, 418-425.*
26. *McNair HA, Mangar SA, Coffey J et al. Comparison of CT and ultrasound-based imaging to localize the prostate for external beam radiotherapy. Int J Radiat Oncol Biol Phys 2006; 65, 678-687.*
27. *Moseley DJ, White EA, Wiltshire KL et al. Comparison of localization performance with implanted fiducial markers and cone-beam computed tomography for on-line image guided radiotherapy to the prostate. Int J Radiat Oncol Biol Phys 2007; 67, 942-953.*
28. *McParland NA. kV-cone beam CT as an IGRT tool in the treatment of early stage prostate cancer: A review of the literature. J Medical Imaging Radiation Science 2009; 40, 9-14.*
29. *Langen KM, Lu W, Willoughby TR et al. Dosimetric effect of prostate motion during helical tomotherapy. Int J Radiat Oncol Biol Phys 2009; 74, 1134-1142.*
30. *DiMuzio N, Fiorino C, Cozzarini C et al. Phase I-II study of hypofractionated simultaneous integrated boost with tomotherapy for prostate cancer. Int J Radiat Oncol Biol Phys 2009; 74, 392-398.*
31. *Fiorini C, Alongi F, Broggi S et al. Physics aspects of prostate tomotherapy: Planning optimization and image guidance issues. Acta Oncol 2008;47;1309-1316.*
32. *IMRT Documentation Working Group, Holmes T, Das R, Low D, Yin FF, Balter J, Palta J, Eifel P; ASTRO. American Society of Radiation Oncology recommendations for documenting intensity-modulated radiation therapy treatments. Int J Radiat Oncol Biol Phys. 2009; 74(5):1311-1318.*
33. *Heaton A, Fenwick JD, Mayles WP, Syndikus I, Wong H. Development of individualised imaging schedules for prostate radiotherapy including on-line tracking techniques. Radiother Oncol 2010 in press.*
